# Lipidomic Profiling Identifies a Novel Lipid Signature Associated with Ethnicity-Specific Disparity of Bladder Cancer

**DOI:** 10.3390/metabo12060544

**Published:** 2022-06-14

**Authors:** Karthik Reddy Kami Reddy, Danthasinghe Waduge Badrajee Piyarathna, Abu Hena Mostafa Kamal, Vasanta Putluri, Shiva Shankar Ravi, Roni J. Bollag, Martha K. Terris, Yair Lotan, Nagireddy Putluri

**Affiliations:** 1 Department of Molecular and Cellular Biology, Baylor College of Medicine, One Baylor Plaza, Houston, TX 77030, USA; karthikr@bcm.edu (K.R.K.R.); piyarath@bcm.edu (D.W.B.P.); shankar.rshiva@gmail.com (S.S.R.); 2 Advanced Technology Cores, Dan L Duncan Comprehensive Cancer Center, Baylor College of Medicine, One Baylor Plaza, Houston, TX 77030, USA; abuhenamostafa.kamal@bcm.edu (A.H.M.K.); vputluri@bcm.edu (V.P.); 3 Georgia Cancer Center, Augusta University, Augusta, GA 30912, USA; rbollag@augusta.edu (R.J.B.); mterris@augusta.edu (M.K.T.); 4 Department of Urology, University of Texas Southwestern, Dallas, TX 75390, USA; yair.lotan@utsouthwestern.edu

**Keywords:** bladder cancer, ethnicity-specific disparity, lipidomics

## Abstract

Bladder Cancer (BLCA) is the ninth most frequently diagnosed cancer globally and the sixth most common cancer in the US. African Americans (AA) exhibit half the BLCA incidence compared to European Americans (EA), but they have a 70% higher risk of cancer-related death; unfortunately, this disparity in BLCA mortality remains poorly understood. In this study, we have used an ethnicity-balanced cohort for unbiased lipidomics profiling to study the changes in the lipid fingerprint for AA and EA BLCA tissues collected from similar geographical regions to determine a signature of ethnic-specific alterations. We identified 86 lipids significantly altered between self-reported AA and EA BLCA patients from Augusta University (AU) cohort. The majority of altered lipids belong to phosphatidylcholines (PCs), phosphatidylethanolamines (PEs), ly sophosphatidylcholines (lysoPCs), phosphatidylserines (PSs), and diglycerides (DGs). Interestingly, levels of four lysoPCs (lyso PCs 20:3, lyso PCs 22:1, lyso PCs 22:2, and lyso PCs 26:1) were elevated while, in contrast, the majority of the PCs were reduced in AA BLCA. Significant alterations in long-chain monounsaturated (MonoUN) and polyunsaturated (PolyUN) lipids were also observed between AA and EA BLCA tumor tissues. These first-in-field results implicate ethnic-specific lipid alterations in BLCA.

## 1. Introduction

Bladder Cancer (BLCA) is the second-most common cancer of the urothelial tract and the fourth most common cancer in men [1,2,3]. While environmental exposures and smoking are known to be critical risk factors, which increase individual risk nearly four times, exposure to different carcinogens is estimated to account for ~50% of BLCA cases [4]. African American (AA) BLCA patients demonstrate inferior oncologic outcomes compared to their European American (EA) counterparts [5,6,7,8,9,10]. Emerging evidence suggests that genetic and other biological factors play a key role in ethnicity-specific disparities associated with BLCA; however, the poor survival of AA patients with BLCA is also due to late diagnoses and more aggressive tumors [11]. For example, five-year cancer-specific survival rates are 67.1% for AAs vs. 78.5% for EAs, [12] and AAs have a 70% higher risk of cancer-related death compared to EAs [13]. They also have almost twice the mortality rate (4.2%) following cystectomy than EAs (2.8%) [14,15]. Apart from environmental and socioeconomic factors, food habits and access to proper healthcare may be significant causes of the increased rates of mortality with AA in various cancers [16]. The identification of key ethnic-specific molecular targets will help us stratify the therapeutic targets that may substantially improve outcomes for AA BLCA patients. Metabolic reprogramming, identified as a key hallmark of cancer [17], is essential for the growth and survival of cancer cells. Earlier, we and others have shown the association of fatty acids with progression in multiple cancers [18,19,20,21,22], making it clearly evident that fatty acid synthesis plays a vital role in cancer development. Recent studies identified that alterations of lipids may impact disease progression in multiple cancers [23]. The inferior survival of AA patients is driven, at least in part, by differential intrinsic tumor biology and lipid metabolism [10], more specifically an altered lipidome. Several studies indicated that different lipids and lipid class has been altered in various cancers [16]. Therefore, lipidomics is proposed as a feasible method to monitor the prognosis and diagnosis specifically for AA BLCA. For BLCA in particular, a few studies have attempted to identify a lipid marker [18,24]. However, alterations of lipids in the context of BLCA health disparities are unknown and remain to be elucidated.

To address this, we utilized a liquid chromatography-high-resolution mass spectrometry (LC-HRMS) platform to conduct a global unbiased lipidomic analysis to identify critical alterations that may contribute to BLCA in AA patients. These findings should improve our understanding of the role of lipid markers in health disparities related to BLCA pathology.

This study contributes novel insights to our understanding of AA BLCA lipid metabolism and may also provide potential lipid markers for prognosis and therapeutic targets for BLCA in AA patients.

## 2. Results

We examined the global lipidomics profile using high-throughput liquid chromatography–high-resolution mass spectrometry (LC-HRMS) in bladder cancer tumors from Augusta University (AU) that included 12 AA BLCA and 14 EA BLCA. Detailed information for the detected lipids from the above samples are given in Supplementary Table S1, and the clinical information (stage, grade, smoke, self-reported ethnicity, etc.) for the samples are given in Supplementary Table S2. The measured lipids with their mass-to-charge ratio (*m*/*z*) and chromatographic retention times are described in earlier publications [16,18,25,26]. The chromatographic reproducibility of the quality controls (sample pool; Supplementary Figure S1) and lipid distribution across all the samples are given in Supplementary Figure S2. A total of 1665 lipids were detected across all the tissues (Supplementary Figure S3A and Supplementary Table S1). These lipids belong to different classes that include cholesteryl esters (CEs), ceramides (Cers), diacylglycerols (DGs), lysophosphatidyl cholines (LysoPCs), lysophosphatidyl ethanolamines (Lyso-PEs), monogalactosyldiacylglycerols (MGDGs), phosphatidic acids (PAs), phosphatidyl cholines (PCs), phosphatidyl ethanolamines (PEs), phosphatidyl glycerols (PGs), phosphatidyl inositols (PIs), plasmenyl-phosphatidyl ethanolamines (plasmenyl -PEs), phosphatidyl serines (PSs), sphingomyelins (SMs), triglycerides (TGs), and an unknown class (Supplementary Figure S3B).

**Figure 1:**
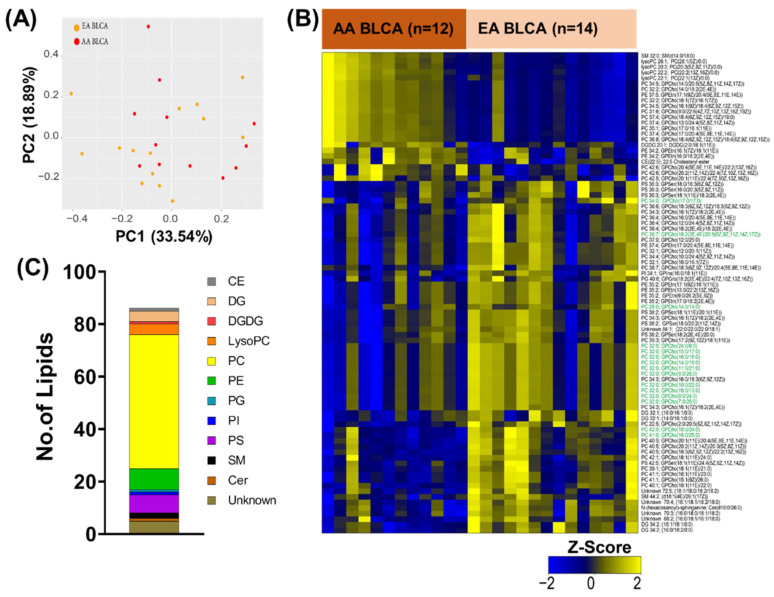
Altered lipidome in ethnicity balanced AA BLCA vs. EA BLCA from AU cohort. (**A**) PCA plot using lipid profiles in AA BLCA (*n* = 12), EA BLCA (*n* = 14) tissues. (**B**) Heat map showing significantly (FDR < 0.25) altered lipids in AA BLCA (*n* = 12) vs. EA BLCA (*n* = 14) tissues. Shades of yellow and blue represent up- and down-regulated lipids, respectively (z-score) (Note: Saturated PCs are highlighted in green). (**C**) Altered lipids from panel B are arranged by class, Cholesterol esters (CEs), Diglycerides (DGs), Lysophosphatidylcholine (Lyso PCs), Phosphatidylcholines (PCs), Phosphatidyl ethanolamines (PEs), Phosphatidylglycerols (PGs), Phosphatidylinositols (PIs), Phosphatidyl Serines (PSs), Sphingomyelins (SMs), Ceramides (Cers), and Unknown lipids.

**Figure 2:**
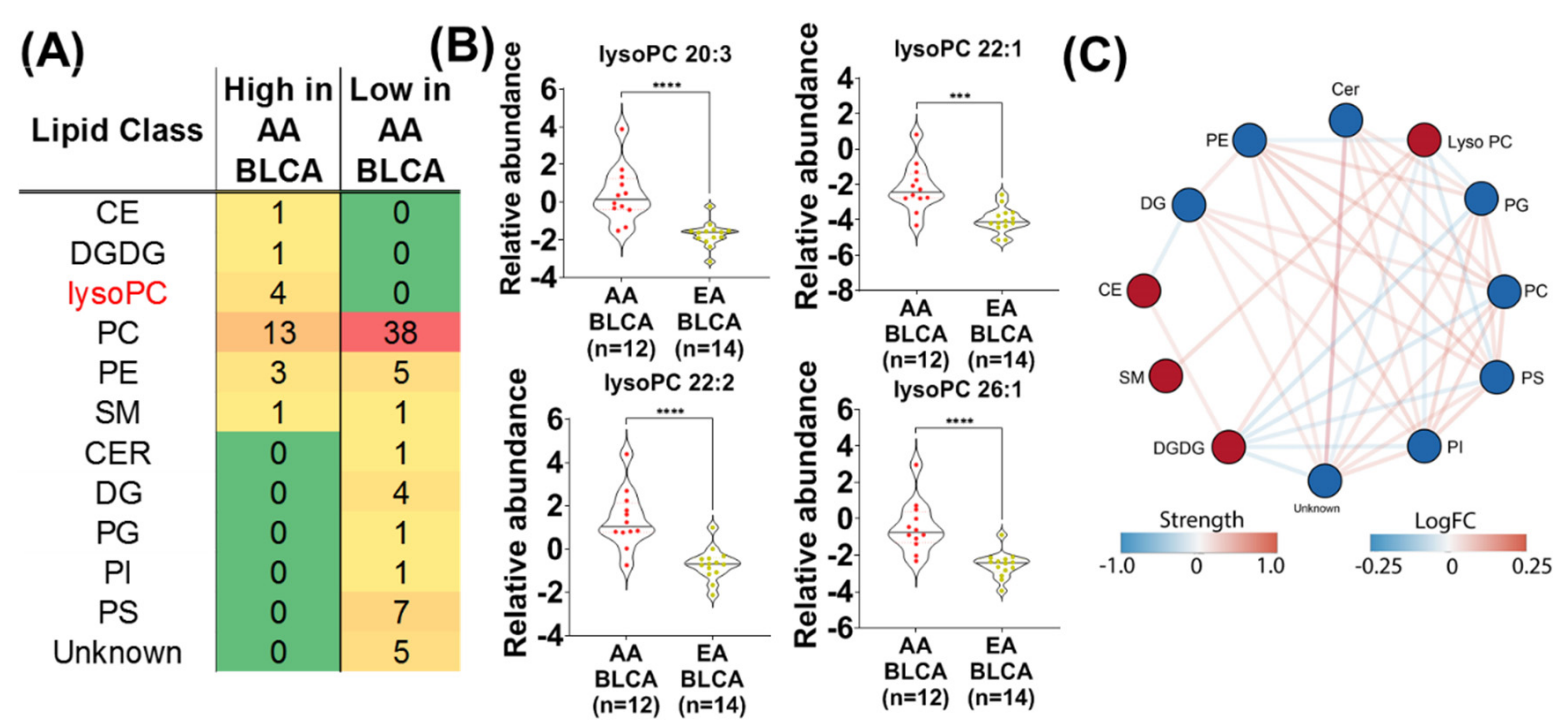
(**A**) Table represents the number of each class of lipids that are high and low in AA BLCA (*n* = 12) compared to EA BLCA (*n* = 14). (**B**) Violin plots represent the expression of four lyso PCs (lyso PCs 20:3, lyso PCs 22:1, lyso PCs 22:2, and lyso PCs 26:1) in AA BLCA (*n* = 12) compared to EA BLCA (*n* = 14) (data from panel A; same lipids represented in panel B) (*t*-test; **** indicating *p* < 0.0001; *** indicating *p* < 0.001). (**C**) A correlation network was inferred for differential lipid classes between AA and EA BLCA. Node color represents the significantly altered lipid species highlighting their decreased (blue) or increased (red) expression. Red edge represents positive correlation and blue edge represents negative correlation.

We performed an analysis of the self-reported ethnicity-balanced AU cohort containing AA (*n* = 12) and EA (*n* = 14) BLCA patients who live in similar geographical regions. As shown in Figure 1A, PCA analysis showed a distribution of samples from the AU cohort. To identify an ethnic-specific signature, we compared global lipid profiles in the ethnicity-balanced AU cohort using a two-sample t-test coupled to Benjamini Hochberg correction for the false discovery rate (FDR). Our data showed that a total of 86 lipids were differently expressed in AA compared to EA BLCA (Figure 1B) at an FDR < 0.25. Particularly, these include CEs, DGs, DGDGs, LysoPCs, PCs, PEs, PGs, PIs, PSs, SMs, Cers, and unknown (Figure 1C). Among the 86 altered lipids, the levels of 23 lipids were elevated and the levels of 63 lipids were reduced (Figure 2A). Interestingly, four unsaturated lyso PCs (lyso PCs 20:3, lyso PCs 22:1, lyso PCs 22:2, and lyso PCs 26:1) were elevated in AA BLCA compared to EA BLCA (Figure 2B) while levels of multiple PCs were reduced (Figure 2A) in AA BLCA compared to EA BLCA. Among them, saturated PC lipids were downregulated in AA BLCA compared to EA BLCA (Figure 1B).

To capture the interaction between the altered lipids belonging to the different lipid classes, we performed a network analysis. Towards this, we first averaged the expression of lipids belonging to each lipid class. This resulted in a class-specific expression value for each lipid class. The class-specific expression values were then used in an enrichment analysis to determine the interaction between different classes of lipids that were altered in AA vs. EA BLCA. Such an analysis revealed a positive correlation between lyso PCs and SMs, and DGDGs. In contrast, lyso PCs were negatively correlated with PIs, PEs, PSs, and PGs (Figure 2C).

**Figure 3:**
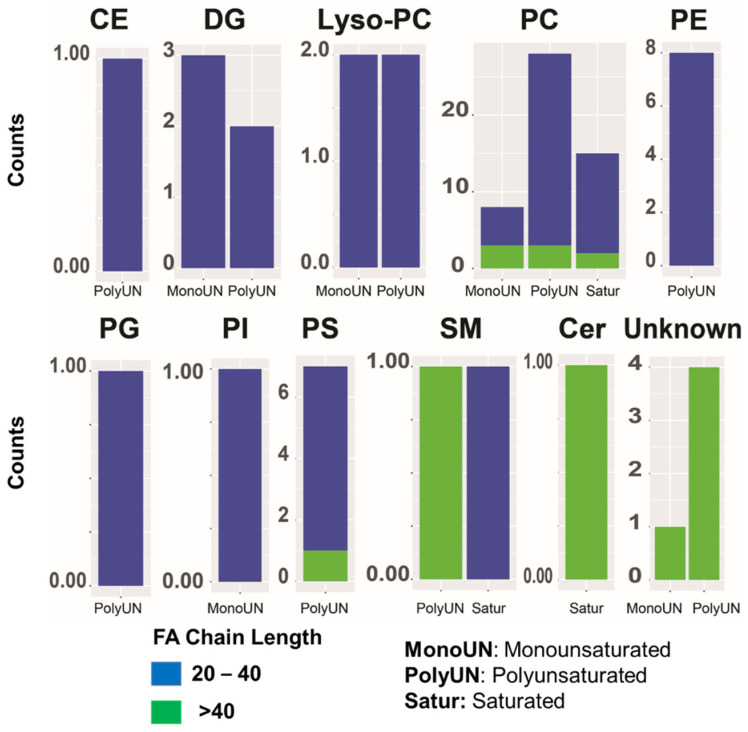
Ethnic-specific lipid-based constituents of major lipid classes and visualization of fatty acid chain composition of differential lipids in BLCA. Bars indicate the number of monounsaturated (MonoUN), polyunsaturated (PolyUN), and saturated (Satur) lipid classes altered in AA BLCA (*n* = 12) vs. EA BLCA (*n* = 14). Differentially expressed lipid classes in AA vs. EA BLCA with 20–40 carbon chain length with blue color and >40 carbon chain length with green color. Note: FA indicates fatty acids.

In addition, we categorized differentially expressed lipid species based on the carbon length and degree of saturation/unsaturation. The former was dichotomized into lipids with fatty acid chains with <40 and >40 carbons. The latter was determined using bond distributions and categorized as saturated (Satur), monounsaturated (MonoUN), and polyunsaturated (PolyUN) lipids. We observed most altered lipids had an average chain length containing 20–40 carbons (Figure 3; Supplementary Table S3 for a list of altered lipids). In addition, the majority of the altered lipids across all classes were polyunsaturated (>2 double bonds) (Figure 3).

## 3. Discussion

The incidence rate of BLCA in AA is nearly half that of EA but AAs exhibit a 70% higher risk of BLCA-related mortality compared to EA even after controlling for comorbid factors. It has been reported that BLCA-specific survival rates in AAs are 67.1% compared to EAs who have 78.5% [13]. Identifying the metabolic pathways that contribute to these ethnic-specific lipid alterations will help to better understand the striking disparities in BLCA mortality and lead to novel therapeutic targets specific for AA BLCA.

Alterations of lipid metabolism, which can modulate different intracellular and intercellular signaling, have been found in many tumor types [27,28]. BLCA lipid metabolism has not been well studied, and we have demonstrated that lipid alterations in BLCA occur in a stage-specific manner [18]. We have also shown altered metabolites in serum from BLCA in the context of ethnic health disparity [11]. However, the association of alterations of lipids in BLCA disparities has been largely unknown. Hence, a better understanding of lipid alterations could lead to the discovery of novel therapeutic targets for AA BLCA. In a first attempt to understand ethnic-specific lipid alterations, we conducted a comprehensive, global, unbiased lipidomics analysis to gain more insight into the lipids associated with BLCA ethnic disparity.

To address this, we used AA and EA BLCA samples obtained from AU cohort. The AU cohort was a balanced collection containing 12 AA and 14 EA samples. The current study has limitations regarding obtaining AA BLCA tissues due to the low incidence rate in the AA population [13]. Our initial analysis comparing the global lipidome yielded 86 significantly altered lipids between AA and EA BLCA.

Nevertheless, our first-in-field results using a cohort of clinically annotated AA BLCA tissues demonstrate that the majority of altered lipids are PEs, LysoPCs, and PCs when comparing AA and EA BLCA. Several studies reported that increased *de novo* lipogenesis is the most common event in early cancer development [29,30], which requires more lipid production in cancer cells [31]. PEs are highly abundant phospholipids in cellular membranes and are found in the plasma membrane and mitochondria in mammalian cells. PEs are involved in many pathological cellular mechanisms such as cell division, death, and the anticoagulant mechanism [32]. Increased expression of the phosphatidyl ethanolamine N-methyltransferase (PEMT) gene, responsible for maintaining PE-PC turnover in cells, correlates with poor patient survival in lung cancer [33]. Interestingly, our study found some of the PE lipid species were increased in AA BLCA patients compared to EA BLCA patients, whereas PCs and LysoPCs are increased in AA BLCA and maintain the conversion reaction between PEs to PCs in cancer cells [33,34]. Previously, high levels of PCs have been seen in multiple cancer types [35]. Our lipidomics landscape also demonstrates high levels of polyunsaturated (PolyUN) lipids in AA BLCA compared to EA BLCA. In line with this, a role for PolyUN in increasing cancer risk and progression, and an association between dietary intake and altered levels of PolyUN lipids, has been reported [36]. Earlier, our group has shown similar associations between dietary data and metabolic alterations in the context of health disparities in prostate cancer [37]. Lipids are vital components of the cell membrane. In particular, lysoPCs and PCs have essential roles in the membrane scaffold and tumor proliferation in AA and disparate gene function, which might lead to altered lipid abundance [38], and we see those high levels of unsaturated lysoPCs in AA BLCA tumors. LysoPC is a key element of phospholipid metabolism, which is converted to PC by the enzymatic activity of phospholipase A2 (PLA2) or lecithin-cholesterol acyltransferase (LCAT) in terms of phospholipid degradation [39]. LysoPCs regulate signal functions and thus may have a tumor-promoting function in AA BLCA. Cholesterol, a key part of the plasma membrane, is also inferred to be associated with cancer metastasis [40]. The excessive free cholesterol is esterified and converted as CEs in lipid droplets by acyl-CoA cholesterol acyltransferase. Earlier, various cancers reported increased CE levels, such as breast cancer, leukemia, glioma, and prostate cancer [41], as increased in AA BLCA.

Collectively, our identifying lipids classes, especially lysoPCs and PCs, resulted in decisive changes in the BLCA lipidome in AA compared to EA patients. Future studies will emphasize the validation of these lipid markers in a larger number of patients since the mortality rate is higher in AA BLCA [5,8].

## 4. Materials and Methods

### 4.1. Patient Sample Information

For this study, human bladder cancer tissues were collected from the state of Georgia (Augusta University (AU)) tumor bank with approved IRB protocol and stored at −140 °C until further analysis. Samples were distributed based on gender [male (*n* = 15) female (*n* = 11)], smoking status (including never, former, current smoker, and unknown), age (42–84 years), different stage of bladder cancer (pTa/Tis, pT1, pT2, pT3, pT4), ethnicity [African American (*n* = 12), European American (*n* = 14)].

### 4.2. Extraction of Lipids

Lipid extraction, mass spectrometry (MS) data acquisition, MS raw data processing, and analysis were described previously in detail [18,20,42,43]. Briefly, the lipids were extracted from the tissues using various solvents (water: methanol: dichloromethane (2:2:2)) at room temperature after spiking a mixture of internal standards (15:0–18:1(d7) DAG, 15:0–18:1(d7) PC, 15:0–18:1(d7) PI (NH4 Salt), 15:0–18:1(d7) PS (Na Salt), and 18:1(d7) Lyso PC) (Avanti polar lipids, Alabaster, AL, USA). Pooled samples were used as quality controls. Lipids were separated by reverse-phase chromatography (Acquity HSS UPLC T3 column (1.8 μm particle 50 × 2.1 mm, Waters, Milford, MA, USA) on a Shimadzu LC system as described previously [18,20]. LC mobile phase A was acetonitrile/water (40:60, *v/v*) with 10 mM ammonium acetate and mobile phase B was acetonitrile/water/isopropanol (10:5:85 *v/v*) with 10 mM ammonium acetate, and the flow rate was 0.4 mL/min.

The data acquisition for each sample was performed in both electrospray ionization (ESI) positive and negative ionization modes using a Turbo DuoSpray^TM^ source on a 5600 TripleTOF (AB Sciex, Concord, ON, Canada), and detailed LC-MS methods were also described previously [18,20,42]. The raw data were converted to the mgf format using proteoWizard software and converted data search through the NIST MS PepSearch Program using LipidBlast libraries; the detailed method was described previously [18,20,42]. The MS/MS identification results from all the files were combined using an in-house software tool to create a library for lipid quantification. For lipid species with several adducts, the sum of spectral peaks from various adducts was used for the subsequent lipids. The identified peaks and retention time were carefully evaluated using MultiQuant software (ver. 1.1.0.26, AB Sciex, Concord, ON, Canada). Quality control samples were monitored for the overall quality of the lipid extraction and mass spectrometry analyses.

### 4.3. Statistical Analysis for Unbiased Lipidomics

The peak area was normalized with an isotopically labeled internal standard followed by log2 transformation of the data for each method prior to analysis. The differentially expressed lipid analysis was evaluated by Student’s t-test followed by the false discovery rate (FDR < 0.25; Benjamini–Hochberg). All analyses were performed in the Python and R statistical environments.

For Figure 1A, the Principal Component Analysis (PCA) plot is generated using all 1665 lipids detected from unbiased lipidomics in AA and EA BLCA. For Figure 1B,C, Figure 2A, Figure 3 (comparing AA BLCA vs. EA BLCA patients), differential lipids were determined by an unpaired t-test (*p* < 0.05), followed by the Benjamini–Hochberg (BH) procedure for false discovery rate correction (FDR < 0.25). For Figure 2B, the FDR value was set at <0.25 (comparing AA BLCA vs. EA BLCA patients), and normalized data were used for lysoPCs. For Figure 2C, the correlation network of differential lipids (derived from Figure 2B with FDR < 0.25) was generated in a class-specific manner. For Figure 3, we plotted based on the number of lipids with their nature of carbon bonds (saturated, monosaturated, and polysaturated) and fatty acid chain lengths from differential lipids (derived from Figure 2B).

## 5. Conclusions

For the first time, our clinical analysis revealed key changes in the BLCA lipidome in AA compared to EA patients by leveraging high-resolution LC-MS-based unbiased lipidomics. Currently, there are no lipid markers for AA BLCA, and the dysregulated lipids that we identified may serve as potential markers that can predict AA BLCA in the future. However, additional studies are needed to address these key findings in the context of BLCA disparities, and to determine if there is a role of body mass index (BMI) in the prognostic significance for BLCA that disproportionately affects the AA population.

## 6. Limitation of Study

Our study has a limitation regarding sample size. Our future study will validate these lipid markers in an independent cohort with a larger number of samples in the context of ethnicity-specific disparity of Bladder Cancer.

## Data Availability

We have uploaded the lipidomics profiling raw data, which will be available in the NIH Metabolomics Workbench (National Metabolomic Data Repository) database with the project ID (PR001271).

## Supplementary Materials

The following supporting information can be downloaded at: https://www.mdpi.com/article/10.3390/metabo12060544/s1, Figure S1. Overlaid total ion chromatogram (TIC) for pool quality control (QC) pool samples (*n* = 6) ran entire sample acquisition (A) ESI-positive mode, (B) ESI-Negative mode. Figure S2. Distribution of lipids detected across all samples from both positive and negative mode (log transformed data). Figure S3. (**A**) Heatmap of total detected lipids in EA BLCA (*n* = 14) and AA BLCA (*n* = 12). Shades of Yellow and Blue represent increased and decreased lipids, respectively (z-score). (**B**) Total detected lipids from panel B are arranged by class wise. Table S1. List of detected lipids (name and mass/charge) by unbiased lipidomics in bladder cancer tissues. (Note: Internal standards were highlighted in red). Table S2. Patient characteristics of bladder cancer tissues used for unbiased lipidomics. Table S3. List of differentially altered lipids between in AU cohort compare between AA and EA (FDR < 0.25, with *p*-value).
