# Supplementary material for: Lipidomic Profiling Identifies a Novel Lipid Signature Associated with Ethnicity-Specific Disparity of Bladder Cancer
Source: Metabolites. 2022 Jun 14;12(6):544. doi: 10.3390/metabo12060544 (PMC9230655; doi:10.3390/metabo12060544)
Supplement: Supplementary file 1 [file metabolites-12-00544-s001.zip › metabolites-1692100-SM.pdf]

(A)

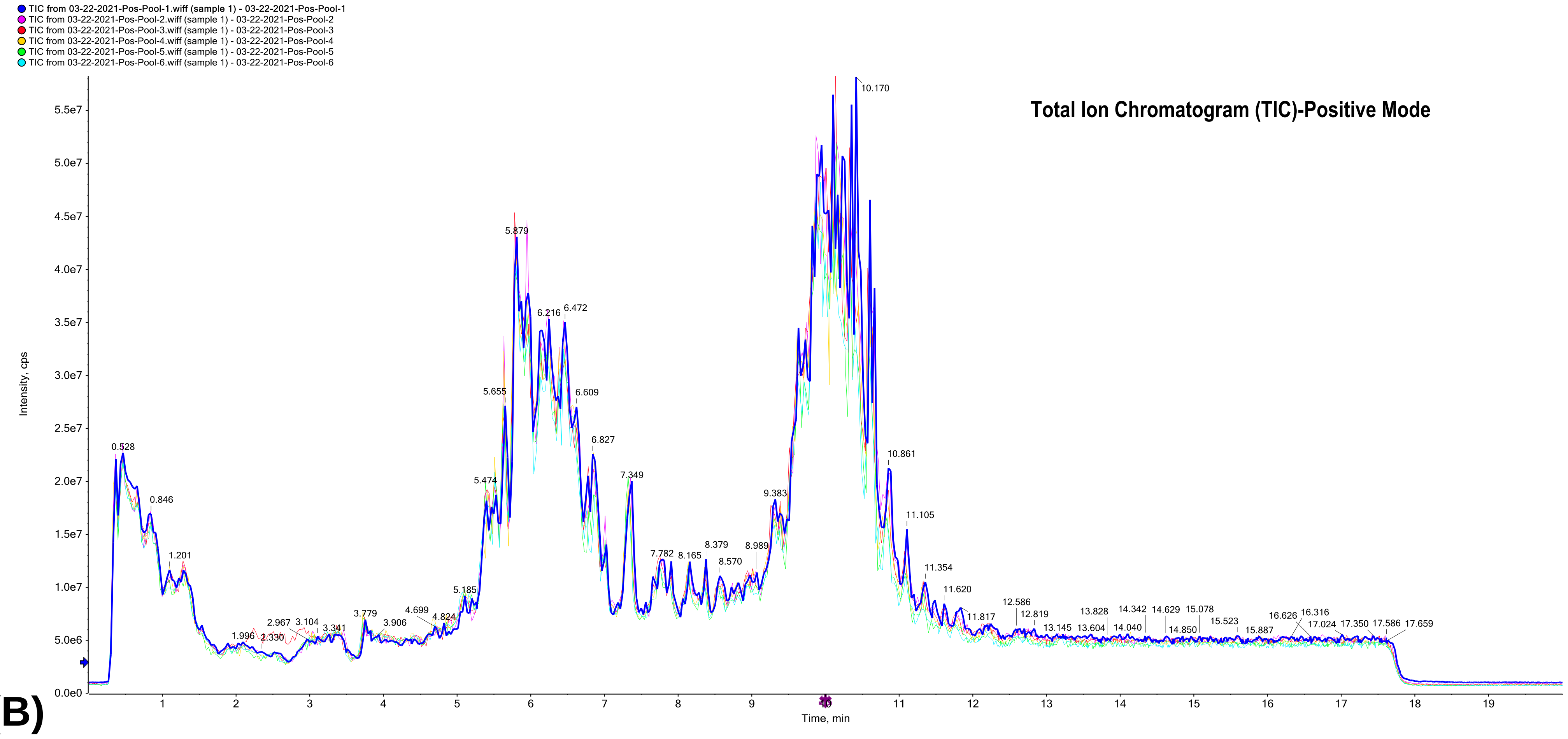

(B)

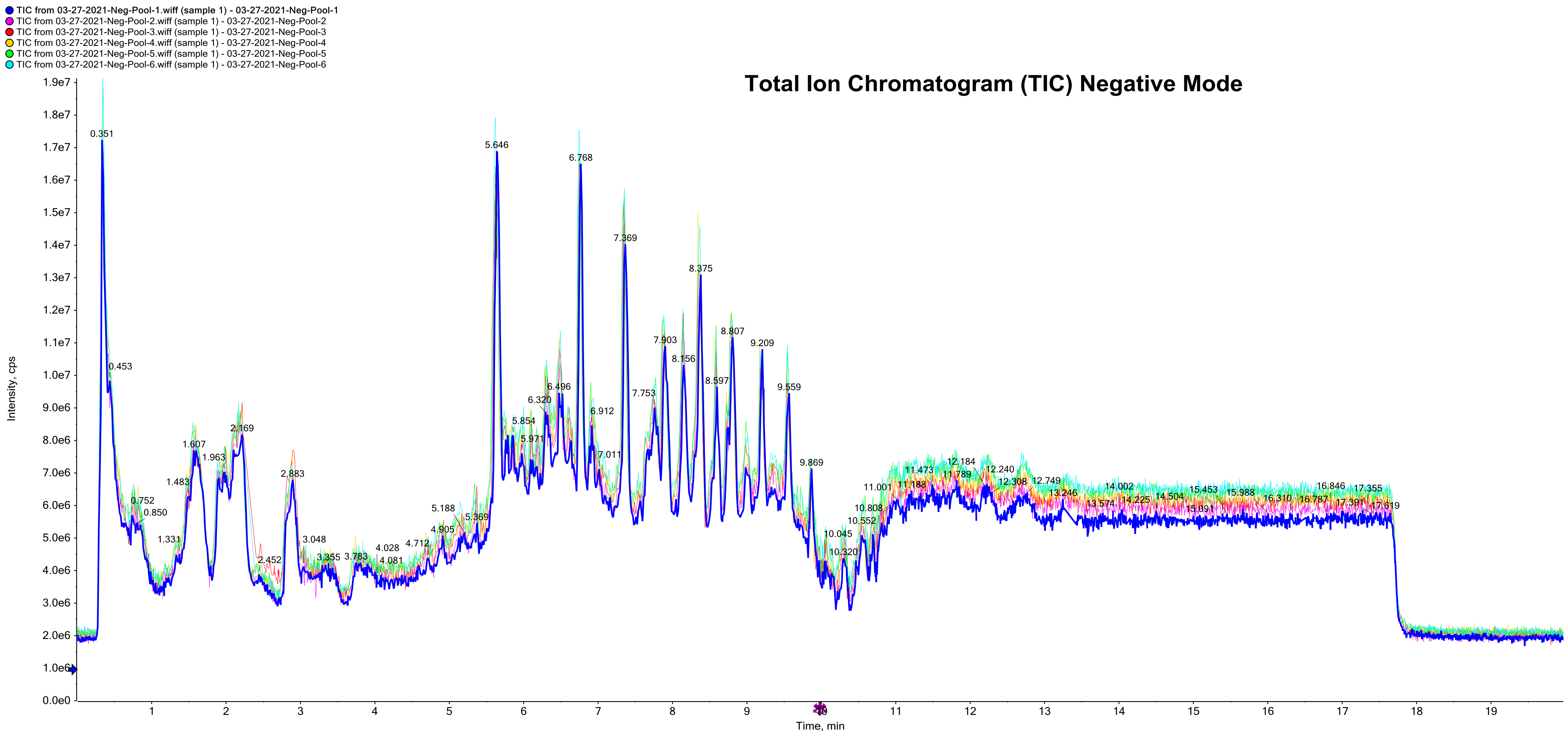

Supplementary Figure S1.

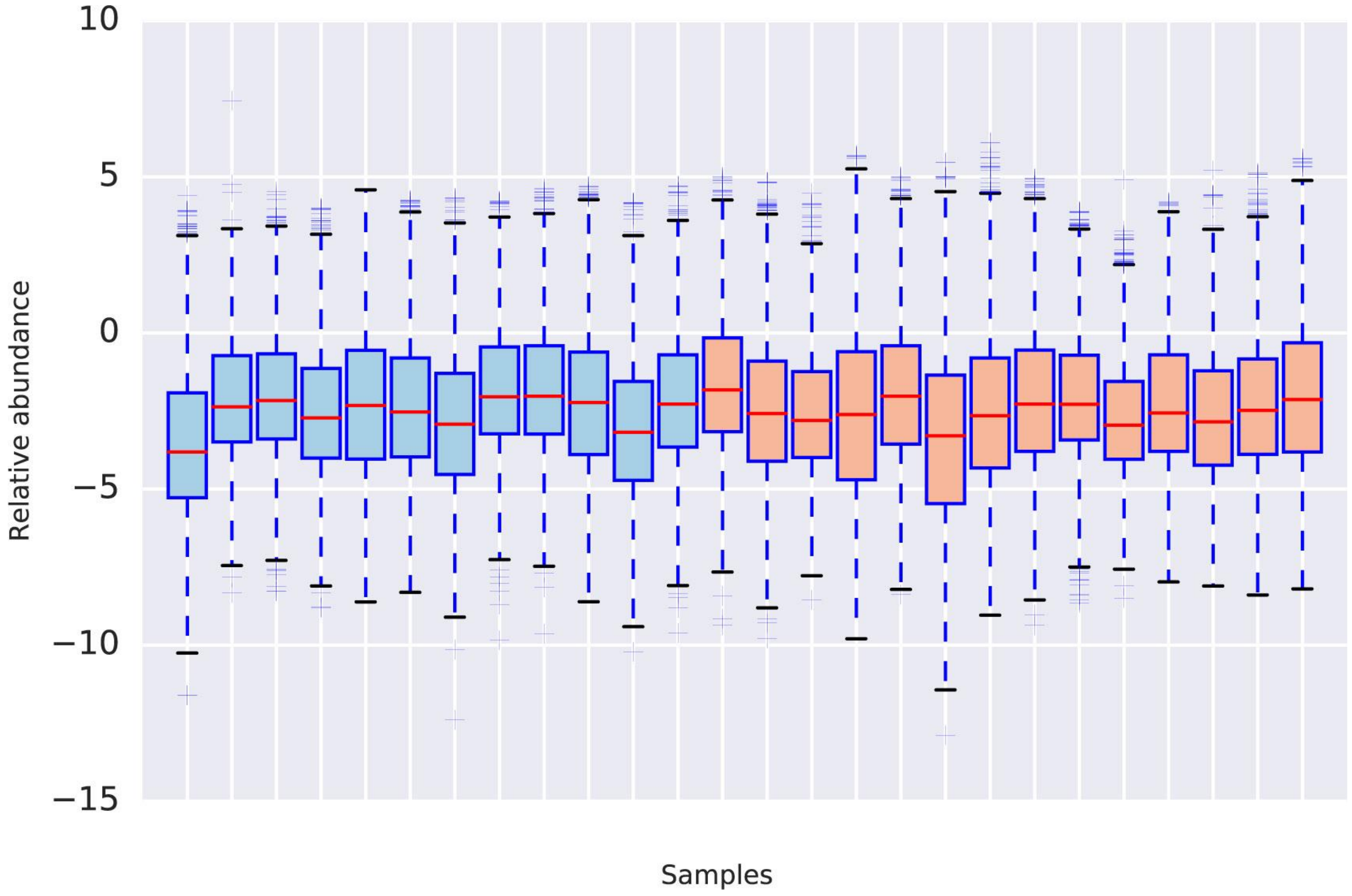

Supplementary Figure S2.

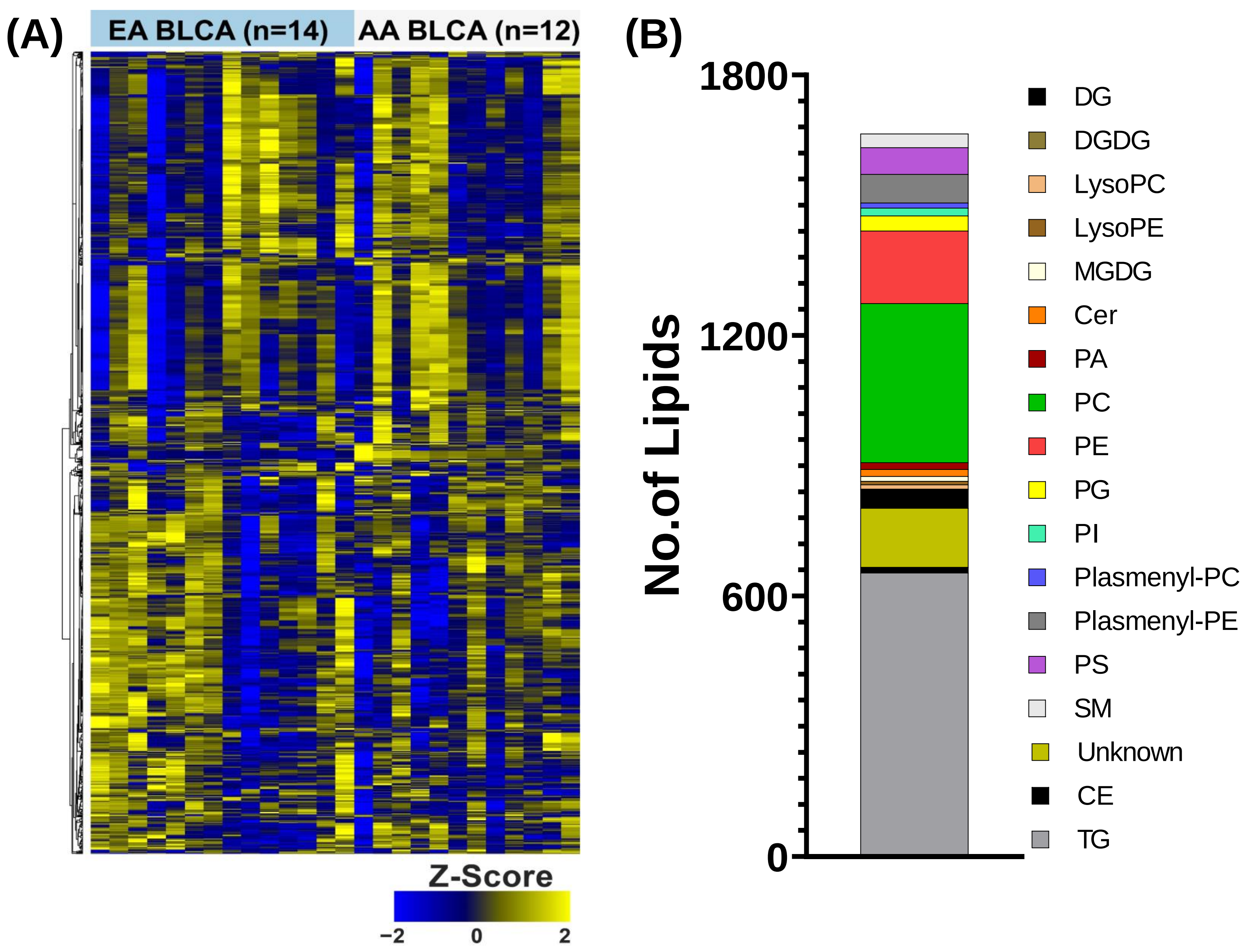

Supplementary Figure S3.

Supplementary Table S1.

ESI Mode	Name	m/z
Positive	CE(16:1); [M+NH4]+; 16:1 Cholesteryl ester	753.61337
Positive	CE(16:2); [M+NH4]+; 16:2 Cholesteryl ester	829.56922
Positive	CE(17:0); [M+NH4]+; 17:0 Cholesteryl ester	754.54842
Positive	CE(17:1); [M+NH4]+; 17:1 Cholesteryl ester	529.39935
Positive	CE(18:1); [M+NH4]+; 18:1 Cholesteryl ester	625.5908
Positive	CE(18:2); [M+NH4]+; 18:2 Cholesteryl ester	642.6148
Positive	CE(18:3); [M+NH4]+; 18:3 Cholesteryl ester	640.6023
Positive	CE(20:3); [M+NH4]+; 20:3 Cholesteryl ester	637.5545
Positive	CE(20:4); [M+NH4]+; 20:4 Cholesteryl ester	656.5998
Positive	CE(22:1); [M+NH4]+; 22:1 Cholesteryl ester	654.5794
Positive	CE(22:2); [M+NH4]+; 22:2 Cholesteryl ester	671.5723
Positive	CE(22:5); [M+NH4]+; 22:5 Cholesteryl ester	668.6332
Positive	CE(22:6); [M+NH4]+; 22:6 Cholesteryl ester	666.6168
Positive	DG 27:2; [M+NH4]+; DG(9:0/18:2/0:0)	664.6005
Positive	DG 28:0; [M+NH4]+; DG(14:0/14:0/0:0)	698.6058
Positive	DG 32:0; [M+NH4]+; DG(16:0/16:0/0:0)	696.6609
Positive	DG 32:1; [M+NH4]+; DG(14:0/18:1/0:0)	694.6457
Positive	DG 32:1; [M+NH4]+; DG(16:0/16:1/0:0)	692.6292
Positive	DG 32:2; [M+NH4]+; DG(14:0/18:2/0:0)	690.6145
Positive	DG 32:2; [M+NH4]+; DG(14:1/18:1/0:0)	688.6055
Positive	DG 32:2; [M+NH4]+; DG(16:1/16:1/0:0)	723.6004
Positive	DG 34:0; [M+NH4]+; DG(16:0/18:0/0:0)	722.5025
Positive	DG 34:1; [M+NH4]+; DG(16:0/18:1/0:0)	718.6499
Positive	DG 34:2; [M+NH4]+; DG(16:0/18:2/0:0)	716.6384
Positive	DG 34:2; [M+NH4]+; DG(16:1/18:1/0:0)	714.6177
Positive	DG 34:3; [M+NH4]+; DG(16:0/18:3/0:0)	751.729
Positive	DG 34:3; [M+NH4]+; DG(16:1/18:2/0:0)	463.1308
Positive	DG 36:1; [M+NH4]+; DG(18:0/18:1/0:0)	476.3814
Positive	DG 36:2; [M+NH4]+; DG(18:0/18:2/0:0)	502.4067
Positive	DG 36:2; [M+NH4]+; DG(18:1/18:1/0:0)	500.4328
Positive	DG 36:3; [M+NH4]+; DG(16:0/20:3/0:0)	498.4139
Positive	DG 36:3; [M+NH4]+; DG(18:1/18:2/0:0)	512.4274
Positive	DG 38:2; [M+NH4]+; DG(18:0/20:2/0:0)	530.4749
Positive	DG 38:2; [M+NH4]+; DG(18:1/20:1/0:0)	530.3963
Positive	DG 38:3; [M+NH4]+; DG(18:0/20:3/0:0)	528.4661
Positive	DG 38:3; [M+NH4]+; DG(18:1/20:2/0:0)	528.4599
Positive	DG 38:4; [M+NH4]+; DG(16:0/22:4/0:0)	526.4443
Positive	DG 38:4; [M+NH4]+; DG(18:0/20:4/0:0)	544.4936

Positive	DG 38:4; [M+NH4]+; DG(18:1/20:3/0:0)	558.5059
Positive	DG 38:4; [M+NH4]+; DG(18:2/20:2/0:0)	556.4949
Positive	DG 38:5; [M+NH4]+; DG(18:2/20:3/0:0)	556.4904
Positive	DG 40:4; [M+NH4]+; DG(16:0/24:4/0:0)	556.491
Positive	DG 40:4; [M+NH4]+; DG(18:0/22:4/0:0)	554.5015
Positive	DG 40:4; [M+NH4]+; DG(18:2/22:2/0:0)	554.4814
Positive	DG 40:4; [M+NH4]+; DG(20:1/20:3/0:0)	540.4161
Positive	DG 40:5; [M+NH4]+; DG(18:0/22:5/0:0)	572.5219
Positive	DG 40:5; [M+NH4]+; DG(18:1/22:4/0:0)	570.5104
Positive	DG 40:6; [M+NH4]+; DG(18:1/22:5/0:0)	568.4926
Positive	DG 40:6; [M+NH4]+; DG(18:2/22:4/0:0)	586.4647
Positive	DG 41:0; [M+NH4]+; DG(16:0/25:0/0:0)	584.5225
Positive	DG 42:10; [M+NH4]+; DG(20:4/22:6/0:0)	584.5237
Positive	DG 42:6; [M+NH4]+; DG(20:0/22:6/0:0)	582.5091
Positive	DG 42:7; [M+Li]+; DG(20:3/22:4/0:0)	582.5102
Positive	DG 42:9; [M+NH4]+; DG(20:3/22:6/0:0)	582.5096
Positive	DG 42:9; [M+NH4]+; DG(20:4/22:5/0:0)	580.4913
Positive	DG 44:9; [M+NH4]+; DG(22:4/22:5/0:0)	578.4814
Positive	lysoPC 15:1; [M+H]+; PC(15:1(9Z)/0:0)	600.5492
Positive	lysoPC 16:0; [M+H]+; PC(O-16:0/0:0)	600.5541
Positive	lysoPC 18:0; [M+H]+; PC(18:0/0:0)	598.5407
Positive	lysoPC 18:1; [M+H]+; PC(18:1(11E)/0:0)	598.5385
Positive	lysoPC 18:2; [M+H]+; PC(18:2(2E,4E)/0:0)	598.5397
Positive	lysoPC 20:3; [M+H]+; PC(20:3(5Z,8Z,11Z)/0:0)	596.5243
Positive	lysoPC 22:1; [M+H]+; PC(22:1(13Z)/0:0)	596.5256
Positive	lysoPC 22:2; [M+H]+; PC(22:2(13Z,16Z)/0:0)	594.509
Positive	lysoPC 23:0; [M+H]+; PC(23:0/0:0)	594.5095
Positive	lysoPC 26:1; [M+H]+; PC(26:1(5Z)/0:0)	614.5718
Positive	MGDG 36:3; [M+Na]+; MGDG(18:1(11E)/18:2(2E,4E))	612.5558
Positive	MGDG 38:5; [M+Na]+; MGDG(18:1(11E)/20:4(5E,8E,11E,14E))	610.5389
Positive	N-(15Z-tetracosenoyl)-sphing-4-enine; [M+H]+; Cer(d18:1(4E)/24:1(15Z))	610.5418
Positive	N-(17Z-hexacosenoyl)-sphing-4-enine; [M+H]+; Cer(d18:1(4E)/26:1(17Z))	608.5267
Positive	N-(17Z-hexacosenoyl)-sphinganine; [M+H]+; Cer(d18:0/26:1(17Z))	608.5225
Positive	N-(5Z,8Z,11Z,14Z-tetracosatetraenoyl)-sphing-4-enine; [M+H]+; Cer(d18:1(4E)/24:4(5Z,8Z,11Z,14Z))	606.5074
Positive	N-(9Z-octadecenoyl)-sphinganine; [M+H]+; Cer(d18:0/18:1(9Z))	628.5856
Positive	N-(docosanoyl)-sphing-4-enine; [M+H]+; Cer(d18:1(4E)/22:0)	628.5887
Positive	N-(docosanoyl)-sphinganine; [M+H]+; Cer(d18:0/22:0)	626.5721
Positive	N-(eicosanoyl)-sphing-4-enine; [M+H]+; Cer(d18:1(4E)/20:0)	626.574
Positive	N-(hexacosanoyl)-sphing-4-enine; [M+H]+; Cer(d18:1(4E)/26:0)	624.5534
Positive	N-(hexacosanoyl)-sphinganine; [M+H]+; Cer(d18:0/26:0)	624.5528
Positive	N-(hexadecanoyl)-sphing-4-enine; [M+H]+; Cer(d18:1(4E)/16:0)	622.5406

Positive	N-(hexadecanoyl)-sphinganine; [M+H]+; Cer(d18:0/16:0)	622.5392
Positive	N-(octadecanoyl)-sphing-4-enine; [M+H]+; Cer(d18:1(4E)/18:0)	620.5282
Positive	N-(tetracosanoyl)-sphing-4-enine; [M+H]+; Cer(d18:1(4E)/24:0)	620.5194
Positive	N-(tetracosanoyl)-sphinganine; [M+H]+; Cer(d18:0/24:0)	642.6009
Positive	PA 36:0; [M+Na2-H]+; GPA(17:0/19:0)	642.6012
Positive	PC 15:0; [M+H]+; GPCho(6:0/9:0)	640.6198
Positive	PC 26:0; [M+Na]+; GPCho(11:0/15:0)	640.5872
Positive	PC 26:0; [M+Na]+; GPCho(6:0/20:0)	639.5166
Positive	PC 27:3; [M+Na]+; GPCho(7:0/20:3(5Z,8Z,11Z))	638.569
Positive	PC 28:3; [M+H]+; GPCho(20:3(5Z,8Z,11Z)/8:0)	638.5716
Positive	PC 29:0; [M+Na]+; GPCho(12:0/17:0)	636.5575
Positive	PC 29:0; [M+Na]+; GPCho(14:0/15:0)	636.5551
Positive	PC 29:2; [M+H]+; GPCho(11:0/18:2(2E,4E))	634.5958
Positive	PC 30:0; [M+Na]+; GPCho(11:0/19:0)	632.5245
Positive	PC 30:0; [M+Na]+; GPCho(12:0/18:0)	656.6144
Positive	PC 31:0; [M+Na]+; GPCho(26:0/5:0)	656.6287
Positive	PC 31:3; [M+H]+; GPCho(13:0/18:3(6Z,9Z,12Z))	654.6081
Positive	PC 31:6; [M+H]+; GPCho(9:0/22:6(4Z,7Z,10Z,13Z,16Z,19Z))	654.6013
Positive	PC 32:0; [M+Na]+; GPCho(10:0/22:0)	652.5874
Positive	PC 32:0; [M+Na]+; GPCho(11:0/21:0)	652.5863
Positive	PC 32:0; [M+Na]+; GPCho(14:0/18:0)	650.571
Positive	PC 32:0; [M+Na]+; GPCho(15:0/17:0)	670.6297
Positive	PC 32:0; [M+Na]+; GPCho(16:0/16:0)	670.6341
Positive	PC 32:0; [M+Na]+; GPCho(19:0/13:0)	670.638
Positive	PC 32:0; [M+Na]+; GPCho(24:0/8:0)	668.6184
Positive	PC 32:0; [M+Na]+; GPCho(6:0/26:0)	668.6162
Positive	PC 32:0; [M+Na]+; GPCho(7:0/25:0)	666.6024
Positive	PC 32:0; [M+Na]+; GPCho(8:0/24:0)	666.6057
Positive	PC 32:1; [M+Na]+; GPCho(12:0/20:1(11Z))	664.5878
Positive	PC 32:1; [M+Na]+; GPCho(16:0/16:1(7Z))	664.5859
Positive	PC 32:2; [M+Na]+; GPCho(14:0/18:2(2E,4E))	664.6357
Positive	PC 32:2; [M+Na]+; GPCho(16:1(7Z)/16:1(7Z))	662.5709
Positive	PC 32:4; [M+H]+; GPCho(14:0/18:4(6Z,9Z,12Z,15Z))	662.5724
Positive	PC 32:4; [M+H]+; GPCho(8:0/24:4(5Z,8Z,11Z,14Z))	662.5725
Positive	PC 32:5; [M+H]+; GPCho(10:0/22:5(4Z,7Z,10Z,13Z,16Z))	662.57
Positive	PC 33:0; [M+Na]+; GPCho(10:0/23:0)	660.5074
Positive	PC 33:2; [M+H]+; GPCho(11:0/22:2(13Z,16Z))	660.5545
Positive	PC 33:2; [M+Na]+; GPCho(7:0/26:2(5E,9Z))	647.5624
Positive	PC 33:5; [M+H]+; GPCho(11:0/22:5(4Z,7Z,10Z,13Z,16Z))	658.5415
Positive	PC 34:0; [M+H]+; GPCho(17:0/17:0)	676.5843
Positive	PC 34:0; [M+Na]+; GPCho(10:0/24:0)	674.5696

Positive	PC 34:0; [M+Na]+; GPCho(12:0/22:0)	698.6641
Positive	PC 34:0; [M+Na]+; GPCho(16:0/18:0)	698.6665
Positive	PC 34:0; [M+Na]+; GPCho(24:0/10:0)	696.6462
Positive	PC 34:0; [M+Na]+; GPCho(9:0/25:0)	696.646
Positive	PC 34:1; [M+Na]+; GPCho(12:0/22:1(13Z))	696.6524
Positive	PC 34:1; [M+Na]+; GPCho(16:0/18:1(11E))	696.647
Positive	PC 34:2; [M+Na]+; GPCho(16:0/18:2(2E,4E))	694.6363
Positive	PC 34:3; [M+H]+; GPCho(16:0/18:3(6Z,9Z,12Z))	694.6326
Positive	PC 34:3; [M+H]+; GPCho(16:1(7Z)/18:2(2E,4E))	694.6365
Positive	PC 34:3; [M+Na]+; GPCho(16:1(7Z)/18:2(2E,4E))	692.619
Positive	PC 34:4; [M+H]+; GPCho(10:0/24:4(5Z,8Z,11Z,14Z))	692.6193
Positive	PC 34:4; [M+H]+; GPCho(22:4(7Z,10Z,13Z,16Z)/12:0)	692.6216
Positive	PC 34:5; [M+H]+; GPCho(14:0/20:5(5Z,8Z,11Z,14Z,17Z))	692.6185
Positive	PC 34:5; [M+H]+; GPCho(16:1(9Z)/18:4(6Z,9Z,12Z,15Z))	690.6005
Positive	PC 34:6; [M+H]+; GPCho(12:0/22:6(4Z,7Z,10Z,13Z,16Z,19Z))	690.6049
Positive	PC 34:6; [M+H]+; GPCho(14:1(9Z)/20:5(5Z,8Z,11Z,14Z,17Z))	690.5985
Positive	PC 35:1; [M+Na]+; GPCho(17:0/18:1(11E))	690.6034
Positive	PC 35:4; [M+H]+; GPCho(11:0/24:4(5Z,8Z,11Z,14Z))	688.5833
Positive	PC 35:5; [M+Na]+; GPCho(13:0/22:5(4Z,7Z,10Z,13Z,16Z))	688.5854
Positive	PC 35:5; [M+Na]+; GPCho(15:0/20:5(5Z,8Z,11Z,14Z,17Z))	686.571
Positive	PC 35:5; [M+Na]+; GPCho(15:1(9Z)/20:4(5E,8E,11E,14E))	686.5724
Positive	PC 35:5; [M+Na]+; GPCho(17:2(9Z,12Z)/18:3(6Z,9Z,12Z))	684.5561
Positive	PC 36:1; [M+H]+; GPCho(12:0/24:1(15Z))	684.5558
Positive	PC 36:1; [M+H]+; GPCho(18:0/18:1(11E))	712.6871
Positive	PC 36:1; [M+Na]+; GPCho(18:0/18:1(11E))	710.6664
Positive	PC 36:2; [M+H]+; GPCho(18:1(11E)/18:1(11E))	708.6466
Positive	PC 36:2; [M+Na]+; GPCho(18:0/18:2(2E,4E))	726.6995
Positive	PC 36:2; [M+Na]+; GPCho(18:1(11E)/18:1(11E))	724.6824
Positive	PC 36:3; [M+H]+; GPCho(16:0/20:3(5Z,8Z,11Z))	724.6843
Positive	PC 36:3; [M+H]+; GPCho(16:0/20:3(8Z,11Z,14Z))	706.5412
Positive	PC 36:3; [M+H]+; GPCho(18:0/18:3(6Z,9Z,12Z))	722.6657
Positive	PC 36:3; [M+H]+; GPCho(18:1(11E)/18:2(2E,4E))	722.6722
Positive	PC 36:4; [M+H]+; GPCho(12:0/24:4(5Z,8Z,11Z,14Z))	722.6618
Positive	PC 36:4; [M+H]+; GPCho(18:2(2E,4E)/18:2(2E,4E))	722.6699
Positive	PC 36:4; [M+Na]+; GPCho(12:0/24:4(5Z,8Z,11Z,14Z))	720.6484
Positive	PC 36:4; [M+Na]+; GPCho(16:0/20:4(5E,8E,11E,14E))	716.6183
Positive	PC 36:4; [M+Na]+; GPCho(18:2(2E,4E)/18:2(2E,4E))	716.6198
Positive	PC 36:6; [M+H]+; GPCho(18:3(6Z,9Z,12Z)/18:3(6Z,9Z,12Z))	714.6155
Positive	PC 36:8; [M+H]+; GPCho(18:4(6Z,9Z,12Z,15Z)/18:4(6Z,9Z,12Z,15Z))	714.6042
Positive	PC 36:8; [M+Na]+; GPCho(18:4(6Z,9Z,12Z,15Z)/18:4(6Z,9Z,12Z,15Z))	702.5427
Positive	PC 37:0; [M+H]+; GPCho(12:0/25:0)	712.5895

Positive	PC 37:4; [M+H]+; GPCho(13:0/24:4(5Z,8Z,11Z,14Z))	708.5584
Positive	PC 37:4; [M+H]+; GPCho(17:0/20:4(5E,8E,11E,14E))	708.5545
Positive	PC 37:4; [M+H]+; GPCho(18:4(6Z,9Z,12Z,15Z)/19:0)	738.6973
Positive	PC 37:7; [M+Na]+; GPCho(17:2(9Z,12Z)/20:5(5Z,8Z,11Z,14Z,17Z))	736.6833
Positive	PC 38:1; [M+H]+; GPCho(17:1(9Z)/21:0)	754.7283
Positive	PC 38:1; [M+Na]+; GPCho(14:1(9Z)/24:0)	752.7112
Positive	PC 38:1; [M+Na]+; GPCho(18:0/20:1(11E))	752.7122
Positive	PC 38:1; [M+Na]+; GPCho(18:1(11E)/20:0)	750.6951
Positive	PC 38:2; [M+H]+; GPCho(14:1(9Z)/24:1(15Z))	750.6935
Positive	PC 38:2; [M+Na]+; GPCho(18:0/20:2(11Z,14Z))	750.6904
Positive	PC 38:2; [M+Na]+; GPCho(18:2(2E,4E)/20:0)	748.6779
Positive	PC 38:3; [M+H]+; GPCho(16:1(7Z)/22:2(13Z,16Z))	748.6787
Positive	PC 38:3; [M+H]+; GPCho(18:0/20:3(5Z,8Z,11Z))	736.5942
Positive	PC 38:3; [M+H]+; GPCho(18:2(2E,4E)/20:1(11E))	772.6761
Positive	PC 38:3; [M+Na]+; GPCho(18:0/20:3(5Z,8Z,11Z))	468.3071
Positive	PC 38:4; [M+Na]+; GPCho(16:0/22:4(7Z,10Z,13Z,16Z))	480.3054
Positive	PC 38:4; [M+Na]+; GPCho(18:0/20:4(5E,8E,11E,14E))	496.3745
Positive	PC 38:5; [M+H]+; GPCho(18:1(11E)/20:4(5E,8E,11E,14E))	482.3594
Positive	PC 38:5; [M+Na]+; GPCho(18:1(11E)/20:4(5E,8E,11E,14E))	494.7734
Positive	PC 38:6; [M+H]+; GPCho(22:6(4Z,7Z,10Z,13Z,16Z,19Z)/16:0)	510.3886
Positive	PC 38:6; [M+Na]+; GPCho(18:4(6Z,9Z,12Z,15Z)/20:2(5Z,8Z))	508.3746
Positive	PC 38:7; [M+H]+; GPCho(18:2(2E,4E)/20:5(5Z,8Z,11Z,14Z,17Z))	506.3597
Positive	PC 38:7; [M+H]+; GPCho(18:3(6Z,9Z,12Z)/20:4(5E,8E,11E,14E))	524.3714
Positive	PC 39:0; [M+Na]+; GPCho(13:0/26:0)	522.355
Positive	PC 39:3; [M+H]+; GPCho(18:3(6Z,9Z,12Z)/21:0)	520.34
Positive	PC 39:6; [M+H]+; GPCho(17:1(9Z)/22:5(4Z,7Z,10Z,13Z,16Z))	538.4188
Positive	PC 39:8; [M+H]+; GPCho(17:2(9Z,12Z)/22:6(4Z,7Z,10Z,13Z,16Z,19Z))	552.4023
Positive	PC 40:0; [M+Na]+; GPCho(16:0/24:0)	536.4062
Positive	PC 40:0; [M+Na]+; GPCho(17:0/23:0)	550.3871
Positive	PC 40:0; [M+Na]+; GPCho(18:0/22:0)	548.3673
Positive	PC 40:1; [M+H]+; GPCho(18:0/22:1(13Z))	546.3993
Positive	PC 40:1; [M+H]+; GPCho(18:1(11E)/22:0)	542.3214
Positive	PC 40:2; [M+Na]+; GPCho(18:1(11E)/22:1(13Z))	566.4521
Positive	PC 40:3; [M+Na]+; GPCho(18:1(11E)/22:2(13Z,16Z))	580.4684
Positive	PC 40:3; [M+Na]+; GPCho(18:2(2E,4E)/22:1(13Z))	578.4112
Positive	PC 40:4; [M+H]+; GPCho(20:2(11Z,14Z)/20:2(11Z,14Z))	576.4035
Positive	PC 40:5; [M+H]+; GPCho(18:0/22:5(4Z,7Z,10Z,13Z,16Z))	572.3688
Positive	PC 40:5; [M+H]+; GPCho(20:0/20:5(5Z,8Z,11Z,14Z,17Z))	570.4638
Positive	PC 40:5; [M+H]+; GPCho(20:2(11Z,14Z)/20:3(5Z,8Z,11Z))	568.3351
Positive	PC 40:5; [M+Na]+; GPCho(16:1(7Z)/24:4(5Z,8Z,11Z,14Z))	594.4786
Positive	PC 40:5; [M+Na]+; GPCho(20:1(11E)/20:4(5E,8E,11E,14E))	608.4965

Positive	PC 40:6; [M+H]+; GPCho(20:3(5Z,8Z,11Z)/20:3(5Z,8Z,11Z))	606.294
Positive	PC 40:6; [M+Na]+; GPCho(18:0/22:6(4Z,7Z,10Z,13Z,16Z,19Z))	600.396
Positive	PC 40:7; [M+H]+; GPCho(20:2(11Z,14Z)/20:5(5Z,8Z,11Z,14Z,17Z))	622.4785
Positive	PC 40:7; [M+H]+; GPCho(20:3(5Z,8Z,11Z)/20:4(5E,8E,11E,14E))	636.4918
Positive	PC 40:7; [M+Na]+; GPCho(18:1(11E)/22:6(4Z,7Z,10Z,13Z,16Z,19Z))	634.4623
Positive	PC 40:7; [M+Na]+; GPCho(20:3(5Z,8Z,11Z)/20:4(5E,8E,11E,14E))	632.4592
Positive	PC 40:8; [M+H]+; GPCho(20:4(5E,8E,11E,14E)/20:4(5E,8E,11E,14E))	504.3021
Positive	PC 40:9; [M+H]+; GPCho(20:4(5E,8E,11E,14E)/20:5(5Z,8Z,11Z,14Z,17Z))	394.3509
Positive	PC 41:1; [M+H]+; GPCho(15:0/26:1(5Z))	435.3468
Positive	PC 41:1; [M+H]+; GPCho(19:0/22:1(13Z))	442.2978
Positive	PC 41:1; [M+H]+; GPCho(20:1(11E)/21:0)	537.5433
Positive	PC 41:1; [M+H]+; GPCho(25:0/16:1(7Z))	567.5472
Positive	PC 41:5; [M+H]+; GPCho(17:1(9Z)/24:4(5Z,8Z,11Z,14Z))	621.6254
Positive	PC 41:5; [M+H]+; GPCho(19:0/22:5(4Z,7Z,10Z,13Z,16Z))	803.7069
Positive	PC 41:5; [M+H]+; GPCho(20:5(5Z,8Z,11Z,14Z,17Z)/21:0)	820.1469
Positive	PC 41:5; [M+H]+; GPCho(24:4(5Z,8Z,11Z,14Z)/17:1(9Z))	818.1127
Positive	PC 41:6; [M+H]+; GPCho(19:0/22:6(4Z,7Z,10Z,13Z,16Z,19Z))	827.7077
Positive	PC 42:0; [M+Na]+; GPCho(16:0/26:0)	858.0448
Positive	PC 42:0; [M+Na]+; GPCho(20:0/22:0)	885.9679
Positive	PC 42:0; [M+Na]+; GPCho(21:0/21:0)	398.3522
Positive	PC 42:1; [M+Na]+; GPCho(16:0/26:1(5Z))	648.6291
Positive	PC 42:3; [M+H]+; GPCho(20:1(11E)/22:2(13Z,16Z))	650.6097
Positive	PC 42:4; [M+H]+; GPCho(18:0/24:4(5Z,8Z,11Z,14Z))	676.6583
Positive	PC 42:4; [M+H]+; GPCho(20:4(5E,8E,11E,14E)/22:0)	678.6738
Positive	PC 42:5; [M+H]+; GPCho(18:1(11E)/24:4(5Z,8Z,11Z,14Z))	642.5925
Positive	PC 42:5; [M+H]+; GPCho(20:1(11E)/22:4(7Z,10Z,13Z,16Z))	644.5943
Positive	PC 42:6; [M+H]+; GPCho(18:2(2E,4E)/24:4(5Z,8Z,11Z,14Z))	564.5323
Positive	PC 42:8; [M+H]+; GPCho(18:4(6Z,9Z,12Z,15Z)/24:4(5Z,8Z,11Z,14Z))	566.5493
Positive	PC 42:8; [M+H]+; GPCho(20:2(11Z,14Z)/22:6(4Z,7Z,10Z,13Z,16Z,19Z))	622.6137
Positive	PC 42:9; [M+H]+; GPCho(20:3(5Z,8Z,11Z)/22:6(4Z,7Z,10Z,13Z,16Z,19Z))	624.6319
Positive	PC 42:9; [M+H]+; GPCho(20:3(8Z,11Z,14Z)/22:6(4Z,7Z,10Z,13Z,16Z,19Z))	482.4544
Positive	PC 42:9; [M+H]+; GPCho(20:4(5E,8E,11E,14E)/22:5(4Z,7Z,10Z,13Z,16Z))	594.5787
Positive	PC 42:9; [M+H]+; GPCho(20:5(5Z,8Z,11Z,14Z,17Z)/22:4(7Z,10Z,13Z,16Z))	596.5925
Positive	PC 43:1; [M+Na]+; GPCho(18:1(11E)/25:0)	678.6748
Positive	PC 43:1; [M+Na]+; GPCho(19:0/24:1(15Z))	680.6904
Positive	PC 43:1; [M+Na]+; GPCho(26:1(5Z)/17:0)	538.5199
Positive	PC 43:4; [M+H]+; GPCho(17:2(9Z,12Z)/26:2(5E,9Z))	540.5307
Positive	PC 43:5; [M+H]+; GPCho(20:5(5Z,8Z,11Z,14Z,17Z)/23:0)	566.5524
Positive	PC 43:6; [M+H]+; GPCho(21:0/22:6(4Z,7Z,10Z,13Z,16Z,19Z))	568.571
Positive	PC 44:0; [M+Na]+; GPCho(26:0/18:0)	650.6413
Positive	PC 44:1; [M+H]+; GPCho(20:0/24:1(15Z))	652.659

Positive	PC 44:12; [M+H]+; GPCho(22:6(4Z,7Z,10Z,13Z,16Z,19Z)/22:6(4Z,7Z,10Z,13Z,16Z,19Z))	510.486
Positive	PC 44:2; [M+H]+; GPCho(22:1(13Z)/22:1(13Z))	512.5021
Positive	PC 44:3; [M+H]+; GPCho(18:3(6Z,9Z,12Z)/26:0)	508.5315
Positive	PC 44:4; [M+H]+; GPCho(18:4(6Z,9Z,12Z,15Z)/26:0)	565.3653
Positive	PC 44:4; [M+H]+; GPCho(20:4(5E,8E,11E,14E)/24:0)	646.647
Positive	PC 44:4; [M+H]+; GPCho(22:2(13Z,16Z)/22:2(13Z,16Z))	734.5267
Positive	PC 44:8; [M+Na]+; GPCho(22:4(7Z,10Z,13Z,16Z)/22:4(7Z,10Z,13Z,16Z))	750.6614
Positive	PC 45:4; [M+H]+; GPCho(20:4(5E,8E,11E,14E)/25:0)	874.0643
Positive	PC 45:4; [M+H]+; GPCho(22:4(7Z,10Z,13Z,16Z)/23:0)	496.3384
Positive	PE 15:0; [M+H]+; GPEtn(11:0/4:0)	530.3562
Positive	PE 15:0; [M+H]+; GPEtn(12:0/3:0)	550.3796
Positive	PE 15:0; [M+H]+; GPEtn(2:0/13:0)	566.3217
Positive	PE 15:0; [M+H]+; GPEtn(3:0/12:0)	566.3212
Positive	PE 15:0; [M+H]+; GPEtn(4:0/11:0)	566.3444
Positive	PE 15:0; [M+H]+; GPEtn(5:0/10:0)	594.3503
Positive	PE 15:0; [M+H]+; GPEtn(6:0/9:0)	658.4758
Positive	PE 16:0; [M+H]+; GPEtn(3:0/13:0)	658.2282
Positive	PE 17:0; [M+H]+; GPEtn(3:0/14:0)	672.4188
Positive	PE 17:0; [M+H]+; GPEtn(4:0/13:0)	672.5249
Positive	PE 17:0; [M+H]+; GPEtn(7:0/10:0)	672.4159
Positive	PE 17:1; [M+H]+; GPEtn(2:0/15:1(9Z))	672.5234
Positive	PE 17:1; [M+H]+; GPEtn(3:0/14:1(9Z))	668.3923
Positive	PE 19:0; [M+H]+; GPEtn(9:0/10:0)	662.4013
Positive	PE 20:0; [M+H]+; GPEtn(4:0/16:0)	684.4212
Positive	PE 31:0; [M+H]+; GPEtn(11:0/20:0)	659.2864
Positive	PE 31:0; [M+H]+; GPEtn(15:0/16:0)	658.401
Positive	PE 31:0; [M+H]+; GPEtn(9:0/22:0)	680.4597
Positive	PE 33:0; [M+H]+; GPEtn(11:0/22:0)	679.5415
Positive	PE 33:0; [M+H]+; GPEtn(16:0/17:0)	700.4889
Positive	PE 33:0; [M+H]+; GPEtn(9:0/24:0)	700.4909
Positive	PE 33:1; [M+H]+; GPEtn(15:0/18:1(11E))	700.4864
Positive	PE 33:1; [M+H]+; GPEtn(16:0/17:1(9Z))	700.4892
Positive	PE 34:0; [M+H]+; GPEtn(11:0/23:0)	672.4211
Positive	PE 34:0; [M+H]+; GPEtn(16:0/18:0)	672.524
Positive	PE 34:0; [M+H]+; GPEtn(9:0/25:0)	672.4208
Positive	PE 34:1; [M+H]+; GPEtn(16:0/18:1(11E))	714.5051
Positive	PE 34:2; [M+H]+; GPEtn(16:0/18:2(2E,4E))	714.5023
Positive	PE 34:2; [M+H]+; GPEtn(16:1(7Z)/18:1(11E))	689.5555
Positive	PE 34:2; [M+Na]+; GPEtn(16:0/18:2(2E,4E))	710.4336
Positive	PE 34:3; [M+H]+; GPEtn(16:0/18:3(6Z,9Z,12Z))	706.4791
Positive	PE 34:3; [M+H]+; GPEtn(17:1(9Z)/17:2(9Z,12Z))	706.483

Positive	PE 35:0; [M+H]+; GPEtn(10:0/25:0)	702.4719
Positive	PE 35:0; [M+H]+; GPEtn(16:0/19:0)	728.5902
Positive	PE 35:0; [M+H]+; GPEtn(9:0/26:0)	728.5202
Positive	PE 35:1; [M+H]+; GPEtn(17:0/18:1(11E))	728.516
Positive	PE 35:2; [M+H]+; GPEtn(13:0/22:2(13Z,16Z))	729.2721
Positive	PE 35:2; [M+H]+; GPEtn(17:0/18:2(2E,4E))	728.5205
Positive	PE 35:2; [M+H]+; GPEtn(17:1(9Z)/18:1(11E))	726.5974
Positive	PE 35:2; [M+H]+; GPEtn(22:2(13Z,16Z)/13:0)	726.5873
Positive	PE 35:2; [M+H]+; GPEtn(9:0/26:2(5E,9Z))	726.4978
Positive	PE 36:1; [M+H]+; GPEtn(18:0/18:1(11E))	726.5001
Positive	PE 36:1; [M+Na]+; GPEtn(18:0/18:1(11E))	726.5004
Positive	PE 36:2; [M+H]+; GPEtn(18:0/18:2(2E,4E))	726.0581
Positive	PE 36:2; [M+H]+; GPEtn(18:1(11E)/18:1(11E))	726.5085
Positive	PE 36:2; [M+Na]+; GPEtn(18:0/18:2(2E,4E))	726.589
Positive	PE 36:2; [M+Na]+; GPEtn(18:1(11E)/18:1(11E))	724.4856
Positive	PE 36:3; [M+H]+; GPEtn(16:0/20:3(5Z,8Z,11Z))	700.4753
Positive	PE 36:3; [M+H]+; GPEtn(18:1(11E)/18:2(2E,4E))	698.4319
Positive	PE 36:4; [M+H]+; GPEtn(16:0/20:4(5E,8E,11E,14E))	698.5061
Positive	PE 36:5; [M+H]+; GPEtn(16:1(7Z)/20:4(5E,8E,11E,14E))	742.2317
Positive	PE 37:2; [M+H]+; GPEtn(11:0/26:2(5E,9Z))	742.5709
Positive	PE 37:3; [M+H]+; GPEtn(18:3(6Z,9Z,12Z)/19:0)	742.2541
Positive	PE 37:4; [M+H]+; GPEtn(17:0/20:4(5E,8E,11E,14E))	742.204
Positive	PE 37:5; [M+H]+; GPEtn(17:1(9Z)/20:4(5E,8E,11E,14E))	714.5045
Positive	PE 37:5; [M+Na]+; GPEtn(17:1(9Z)/20:4(5E,8E,11E,14E))	735.7444
Positive	PE 38:4; [M+H]+; GPEtn(18:0/20:4(5E,8E,11E,14E))	732.4751
Positive	PE 38:4; [M+Na]+; GPEtn(18:0/20:4(5E,8E,11E,14E))	732.4665
Positive	PE 38:6; [M+H]+; GPEtn(16:0/22:6(4Z,7Z,10Z,13Z,16Z,19Z))	733.5139
Positive	PE 38:6; [M+H]+; GPEtn(18:2(2E,4E)/20:4(5E,8E,11E,14E))	708.4865
Positive	PE 39:2; [M+H]+; GPEtn(13:0/26:2(5E,9Z))	730.4794
Positive	PE 39:2; [M+Na]+; GPEtn(22:1(13Z)/17:1(9Z))	734.5008
Positive	PE 40:2; [M+H]+; GPEtn(18:1(11E)/22:1(13Z))	756.5501
Positive	PE 40:6; [M+H]+; GPEtn(18:0/22:6(4Z,7Z,10Z,13Z,16Z,19Z))	756.5521
Positive	PE 40:7; [M+H]+; GPEtn(18:1(11E)/22:6(4Z,7Z,10Z,13Z,16Z,19Z))	756.5517
Positive	PE 40:9; [M+H]+; GPEtn(18:4(6Z,9Z,12Z,15Z)/22:5(4Z,7Z,10Z,13Z,16Z))	756.5528
Positive	PE 41:2; [M+H]+; GPEtn(18:2(2E,4E)/23:0)	756.5536
Positive	PE 41:3; [M+Na]+; GPEtn(15:1(9Z)/26:2(5Z,9E))	756.5489
Positive	PE 41:3; [M+Na]+; GPEtn(17:2(9Z,12Z)/24:1(15Z))	756.553
Positive	PE 41:4; [M+Na]+; GPEtn(19:0/22:4(7Z,10Z,13Z,16Z))	756.5555
Positive	PE 41:4; [M+Na]+; GPEtn(20:4(5E,8E,11E,14E)/21:0)	756.5498
Positive	PE 43:6; [M+Na]+; GPEtn(21:0/22:6(4Z,7Z,10Z,13Z,16Z,19Z))	756.5517
Positive	PE 44:4; [M+H]+; GPEtn(18:2(2E,4E)/26:2(5E,9Z))	732.4944

Positive	PE 46:4; [M+H]+; GPEtn(20:2(11Z,14Z)/26:2(5E,9Z))	732.4944
Positive	plasmenyl-PC 18:0; [M+Na]+; PC(P-16:0/2:0)	753.6945
Positive	plasmenyl-PC 35:0; [M+Na]+; PC(P-16:0/19:0)	754.5413
Positive	plasmenyl-PC 36:0; [M+Na]+; PC(P-16:0/20:0)	754.5348
Positive	plasmenyl-PC 36:4; [M+Na]+; PC(P-16:0/20:4(5E,8E,11E,14E))	752.5226
Positive	plasmenyl-PC 38:0; [M+Na]+; PC(P-16:0/22:0)	752.5199
Positive	plasmenyl-PC 38:1; [M+Na]+; PC(P-16:0/22:1(13Z))	728.5228
Positive	plasmenyl-PC 38:4; [M+Na]+; PC(P-18:0/20:4(5E,8E,11E,14E))	727.5757
Positive	plasmenyl-PC 39:0; [M+Na]+; PC(P-20:0/19:0)	726.5351
Positive	plasmenyl-PC 40:0; [M+Na]+; PC(P-16:0/24:0)	726.5451
Positive	plasmenyl-PC 42:1; [M+Na]+; PC(P-18:0/24:1(15Z))	724.5346
Positive	plasmenyl-PC 42:2; [M+Na]+; PC(P-20:0/22:2(13Z,16Z))	722.4689
Positive	plasmenyl-PC 43:0; [M+Na]+; PC(P-20:0/23:0)	770.5365
Positive	plasmenyl-PE 34:0; [M+Na]+; PE(P-18:0/16:0)	770.5308
Positive	plasmenyl-PE 34:1; [M+H]+; PE(P-16:0/18:1(11E))	770.5219
Positive	plasmenyl-PE 34:1; [M+Na]+; PE(P-16:0/18:1(11E))	744.5582
Positive	plasmenyl-PE 36:1; [M+Na]+; PE(P-18:0/18:1(11E))	766.5699
Positive	plasmenyl-PE 36:3; [M+H]+; PE(P-18:0/18:3(6Z,9Z,12Z))	742.2295
Positive	plasmenyl-PE 36:4; [M+H]+; PE(P-16:0/20:4(5E,8E,11E,14E))	742.2217
Positive	plasmenyl-PE 36:5; [M+H]+; PE(P-16:0/20:5(5Z,8Z,11Z,14Z,17Z))	740.2456
Positive	plasmenyl-PE 38:1; [M+H]+; PE(P-18:0/20:1(11E))	737.5737
Positive	plasmenyl-PE 38:1; [M+Na]+; PE(P-16:0/22:1(13Z))	762.5263
Positive	plasmenyl-PE 38:1; [M+Na]+; PE(P-18:0/20:1(11E))	784.578
Positive	plasmenyl-PE 38:1; [M+Na]+; PE(P-20:0/18:1(11E))	784.5846
Positive	plasmenyl-PE 38:2; [M+Na]+; PE(P-20:0/18:2(2E,4E))	784.5823
Positive	plasmenyl-PE 38:3; [M+H]+; PE(P-18:0/20:3(5Z,8Z,11Z))	784.5819
Positive	plasmenyl-PE 38:3; [M+Na]+; PE(P-18:0/20:3(5Z,8Z,11Z))	785.1752
Positive	plasmenyl-PE 38:4; [M+H]+; PE(P-16:0/22:4(7Z,10Z,13Z,16Z))	784.5797
Positive	plasmenyl-PE 38:4; [M+H]+; PE(P-18:0/20:4(5E,8E,11E,14E))	783.5877
Positive	plasmenyl-PE 38:4; [M+Na]+; PE(P-18:0/20:4(5E,8E,11E,14E))	782.569
Positive	plasmenyl-PE 38:5; [M+H]+; PE(P-16:0/22:5(4Z,7Z,10Z,13Z,16Z))	780.392
Positive	plasmenyl-PE 38:5; [M+H]+; PE(P-18:0/20:5(5Z,8Z,11Z,14Z,17Z))	780.552
Positive	plasmenyl-PE 38:6; [M+H]+; PE(P-16:0/22:6(4Z,7Z,10Z,13Z,16Z,19Z))	781.0598
Positive	plasmenyl-PE 40:1; [M+Na]+; PE(P-18:0/22:1(13Z))	756.5506
Positive	plasmenyl-PE 40:1; [M+Na]+; PE(P-20:0/20:1(11E))	756.5502
Positive	plasmenyl-PE 40:2; [M+Na]+; PE(P-18:0/22:2(13Z,16Z))	778.5308
Positive	plasmenyl-PE 40:3; [M+H]+; PE(P-20:0/20:3(5Z,8Z,11Z))	754.5371
Positive	plasmenyl-PE 40:4; [M+H]+; PE(P-18:0/22:4(7Z,10Z,13Z,16Z))	754.1582
Positive	plasmenyl-PE 40:4; [M+H]+; PE(P-20:0/20:4(5E,8E,11E,14E))	755.6038
Positive	plasmenyl-PE 40:5; [M+H]+; PE(P-18:0/22:5(4Z,7Z,10Z,13Z,16Z))	776.5196
Positive	plasmenyl-PE 40:6; [M+H]+; PE(P-18:0/22:6(4Z,7Z,10Z,13Z,16Z,19Z))	776.519

Positive	plasmenyl-PE 42:1; [M+Na]+; PE(P-16:0/26:1(5Z))	776.5233
Positive	plasmenyl-PE 42:2; [M+Na]+; PE(P-16:0/26:2(5E,9Z))	752.3802
Positive	plasmenyl-PE 42:2; [M+Na]+; PE(P-20:0/22:2(13Z,16Z))	752.5216
Positive	plasmenyl-PE 42:4; [M+H]+; PE(P-18:0/24:4(5Z,8Z,11Z,14Z))	752.3683
Positive	PS 42:7; [M+H]+; GPSer(18:3(6Z,9Z,12Z)/24:4(5Z,8Z,11Z,14Z))	752.5172
Positive	SM 32:0; [M]+; SM(d14:0/18:0)	750.524
Positive	SM 35:0; [M+Na]+; SM(d15:0/20:0)	750.5116
Positive	SM 36:2; [M+Na]+; SM(d18:1(4E)/18:1(9Z))	776.5263
Positive	SM 38:0; [M+Na]+; SM(d14:0/24:0)	776.5216
Positive	SM 38:1; [M]+; SM(d14:0/24:1(15Z))	799.5251
Positive	SM 39:0; [M]+; SM(d15:0/24:0)	796.5412
Positive	SM 39:0; [M+Na]+; SM(d15:0/24:0)	773.6823
Positive	SM 39:1; [M+Na]+; SM(d15:0/24:1(15Z))	768.5478
Positive	SM 39:4; [M+Na]+; SM(d15:0/24:4(5Z,8Z,11Z,14Z))	766.1261
Positive	SM 40:0; [M]+; SM(d14:0/26:0)	766.1301
Positive	SM 40:0; [M+Na]+; SM(d14:0/26:0)	788.5575
Positive	SM 40:1; [M]+; SM(d14:0/26:1(17Z))	788.5167
Positive	SM 40:1; [M+Na]+; SM(d14:0/26:1(17Z))	788.5577
Positive	SM 41:0; [M+Na]+; SM(d15:0/26:0)	788.5568
Positive	SM 41:1; [M+Na]+; SM(d15:0/26:1(17Z))	764.1585
Positive	SM 41:2; [M+Na]+; SM(d15:1(4E)/26:1(17Z))	792.2082
Positive	SM 41:5; [M]+; SM(d17:1(4E)/24:4(5Z,8Z,11Z,14Z))	789.6591
Positive	SM 42:0; [M+Na]+; SM(d16:0/26:0)	788.1871
Positive	SM 42:1; [M]+; SM(d16:0/26:1(17Z))	789.5939
Positive	SM 42:1; [M+Na]+; SM(d16:0/26:1(17Z))	811.1008
Positive	SM 42:2; [M]+; SM(d16:1(4E)/26:1(17Z))	811.0844
Positive	SM 42:2; [M+Na]+; SM(d16:1(4E)/26:1(17Z))	811.107
Positive	SM 43:0; [M+Na]+; SM(d17:0/26:0)	810.6007
Positive	SM 43:1; [M+Na]+; SM(d17:0/26:1(17Z))	786.601
Positive	SM 43:2; [M]+; SM(d17:1(4E)/26:1(17Z))	808.5834
Positive	SM 43:2; [M+Na]+; SM(d17:1(4E)/26:1(17Z))	808.5883
Positive	SM 43:5; [M]+; SM(d19:1(4E)/24:4(5Z,8Z,11Z,14Z))	784.5816
Positive	SM 44:0; [M+Na]+; SM(d18:0/26:0)	784.5851
Positive	SM 44:2; [M+Na]+; SM(d18:1(4E)/26:1(17Z))	784.5809
Positive	SM 45:0; [M+Na]+; SM(d19:0/26:0)	784.5848
Positive	SM 45:1; [M+Na]+; SM(d19:0/26:1(17Z))	783.6346
Positive	SM 45:2; [M]+; SM(d19:1(4E)/26:1(17Z))	782.5997
Positive	TG 40:0; [M+Na]+; TG(12:0/12:0/16:0)	804.5508
Positive	TG 40:0; [M+NH4]+; TG(12:0/12:0/16:0)	804.5512
Positive	TG 40:1; [M+NH4]+; TG(12:0/12:0/16:1)	804.5516
Positive	TG 42:0; [M+NH4]+; TG(12:0/12:0/18:0)	780.0974

Positive	TG 42:0; [M+NH4]+; TG(12:0/14:0/16:0)	780.4064
Positive	TG 42:1; [M+NH4]+; TG(12:0/12:0/18:1)	781.0773
Positive	TG 42:2; [M+NH4]+; TG(12:0/14:1/16:1)	780.3938
Positive	TG 44:1; [M+Na]+; TG(12:0/16:1/16:0)	780.0932
Positive	TG 44:1; [M+Na]+; TG(14:0/16:1/14:0)	802.5289
Positive	TG 44:1; [M+NH4]+; TG(12:0/16:1/16:0)	802.5403
Positive	TG 44:2; [M+Na]+; TG(12:0/14:1/18:1)	802.5372
Positive	TG 44:2; [M+NH4]+; TG(12:0/14:1/18:1)	802.5461
Positive	TG 46:0; [M+Na]+; TG(12:0/16:0/18:0)	778.5356
Positive	TG 46:0; [M+Na]+; TG(14:0/16:0/16:0)	776.5185
Positive	TG 46:0; [M+NH4]+; TG(12:0/16:0/18:0)	776.5241
Positive	TG 46:0; [M+NH4]+; TG(14:0/16:0/16:0)	774.5698
Positive	TG 46:1; [M+Na]+; TG(14:0/16:0/16:1)	796.5358
Positive	TG 46:1; [M+NH4]+; TG(14:0/16:0/16:1)	805.5473
Positive	TG 46:2; [M+Na]+; TG(12:0/18:1/16:1)	824.6059
Positive	TG 46:2; [M+NH4]+; TG(12:0/18:1/16:1)	820.5393
Positive	TG 46:3; [M+NH4]+; TG(14:1/16:1/16:1)	796.5451
Positive	TG 48:0; [M+Na]+; TG(14:0/16:0/18:0)	796.5383
Positive	TG 48:0; [M+NH4]+; TG(14:0/16:0/18:0)	796.543
Positive	TG 48:1; [M+Li]+; TG(14:0/16:0/18:1)	794.531
Positive	TG 48:1; [M+Na]+; TG(14:0/16:0/18:1)	794.5362
Positive	TG 48:1; [M+Na]+; TG(14:0/16:1/18:0)	794.5213
Positive	TG 48:1; [M+Na]+; TG(16:0/16:0/16:1)	813.705
Positive	TG 48:1; [M+NH4]+; TG(14:0/16:0/18:1)	840.7337
Positive	TG 48:1; [M+NH4]+; TG(14:0/16:1/18:0)	840.734
Positive	TG 48:1; [M+NH4]+; TG(16:0/16:0/16:1)	816.5815
Positive	TG 48:2; [M+Na]+; TG(14:0/16:1/18:1)	818.1327
Positive	TG 48:2; [M+Na]+; TG(16:0/16:1/16:1)	838.1831
Positive	TG 48:2; [M+NH4]+; TG(14:0/16:1/18:1)	838.6284
Positive	TG 48:2; [M+NH4]+; TG(16:0/16:1/16:1)	838.7353
Positive	TG 48:3; [M+Na]+; TG(14:1/16:1/18:1)	838.7284
Positive	TG 48:3; [M+NH4]+; TG(14:1/16:1/18:1)	838.184
Positive	TG 49:0; [M+Na]+; TG(16:0/16:0/17:0)	838.6301
Positive	TG 49:1; [M+Na]+; TG(16:0/16:0/17:1)	838.6249
Positive	TG 49:1; [M+Na]+; TG(16:0/16:1/17:0)	838.7229
Positive	TG 49:1; [M+NH4]+; TG(16:0/16:0/17:1)	813.6957
Positive	TG 49:2; [M+NH4]+; TG(16:0/16:0/17:2)	836.7068
Positive	TG 49:2; [M+NH4]+; TG(16:0/16:1/17:1)	836.6169
Positive	TG 49:3; [M+Na]+; TG(16:1/16:1/17:1)	836.7199
Positive	TG 49:3; [M+NH4]+; TG(16:0/16:1/17:2)	836.6058
Positive	TG 49:3; [M+NH4]+; TG(16:1/16:1/17:1)	812.671

Positive	TG 50:0; [M+Na]+; TG(16:0/16:0/18:0)	813.6371
Positive	TG 50:0; [M+NH4]+; TG(16:0/16:0/18:0)	811.6541
Positive	TG 50:1; [M+Na]+; TG(16:0/16:0/18:1)	834.5977
Positive	TG 50:1; [M+Na]+; TG(16:0/16:1/18:0)	811.1169
Positive	TG 50:1; [M+NH4]+; TG(14:0/18:0/18:1)	811.1452
Positive	TG 50:1; [M+NH4]+; TG(16:0/17:0/17:1)	811.1106
Positive	TG 50:2; [M+Na]+; TG(16:0/16:0/18:2)	810.08
Positive	TG 50:2; [M+Na]+; TG(16:0/16:1/18:1)	811.1266
Positive	TG 50:2; [M+NH4]+; TG(16:0/16:0/18:2)	811.1013
Positive	TG 50:2; [M+NH4]+; TG(16:0/16:1/18:1)	811.1394
Positive	TG 50:2; [M+NH4]+; TG(16:0/17:1/17:1)	811.1182
Positive	TG 50:2; [M+NH4]+; TG(16:1/17:0/17:1)	810.5347
Positive	TG 50:3; [M+Na]+; TG(16:0/16:0/18:3)	832.5797
Positive	TG 50:3; [M+Na]+; TG(16:0/16:1/18:2)	832.5835
Positive	TG 50:3; [M+Na]+; TG(16:1/16:1/18:1)	809.079
Positive	TG 50:3; [M+NH4]+; TG(16:0/16:1/18:2)	808.0709
Positive	TG 50:3; [M+NH4]+; TG(16:1/16:1/18:1)	808.028
Positive	TG 50:3; [M+NH4]+; TG(16:1/17:0/17:2)	808.0682
Positive	TG 50:3; [M+NH4]+; TG(16:1/17:1/17:1)	808.5845
Positive	TG 50:4; [M+Na]+; TG(16:0/16:1/18:3)	809.0056
Positive	TG 50:4; [M+Na]+; TG(16:1/16:1/18:2)	808.0354
Positive	TG 50:4; [M+NH4]+; TG(16:0/16:1/18:3)	809.0645
Positive	TG 50:4; [M+NH4]+; TG(16:1/16:1/18:2)	830.5718
Positive	TG 50:5; [M+Na]+; TG(16:1/16:1/18:3)	806.0399
Positive	TG 50:5; [M+NH4]+; TG(16:1/16:1/18:3)	806.0081
Positive	TG 51:0; [M+Na]+; TG(16:0/16:0/19:0)	806.0795
Positive	TG 51:0; [M+Na]+; TG(16:0/17:0/18:0)	806.0669
Positive	TG 51:0; [M+NH4]+; TG(16:0/16:0/19:0)	806.5647
Positive	TG 51:1; [M+Na]+; TG(16:0/17:0/18:1)	828.5531
Positive	TG 51:1; [M+Na]+; TG(16:0/17:1/18:0)	828.5527
Positive	TG 51:1; [M+Na]+; TG(16:1/17:0/18:0)	828.5528
Positive	TG 51:1; [M+NH4]+; TG(16:0/16:1/19:0)	828.5561
Positive	TG 51:1; [M+NH4]+; TG(16:0/17:0/18:1)	828.546
Positive	TG 51:1; [M+NH4]+; TG(16:0/17:1/18:0)	828.5506
Positive	TG 51:1; [M+NH4]+; TG(16:1/17:0/18:0)	829.7657
Positive	TG 51:2; [M+Na]+; TG(17:0/17:1/17:1)	804.5481
Positive	TG 51:2; [M+NH4]+; TG(16:0/17:0/18:2)	804.5445
Positive	TG 51:2; [M+NH4]+; TG(16:0/17:1/18:1)	804.0146
Positive	TG 51:2; [M+NH4]+; TG(16:1/17:0/18:1)	802.5965
Positive	TG 51:2; [M+NH4]+; TG(17:0/17:1/17:1)	802.5697
Positive	TG 51:3; [M+Na]+; TG(17:0/17:1/17:2)	802.5364

Positive	TG 51:3; [M+NH4]+; TG(16:0/17:2/18:1)	800.5782
Positive	TG 51:3; [M+NH4]+; TG(16:1/17:1/18:1)	854.5829
Positive	TG 51:3; [M+NH4]+; TG(17:0/17:1/17:2)	854.5948
Positive	TG 51:4; [M+Li]+; TG(16:1/17:1/18:2)	829.051
Positive	TG 51:4; [M+NH4]+; TG(16:0/17:1/18:3)	850.6763
Positive	TG 51:4; [M+NH4]+; TG(16:0/17:2/18:2)	850.6773
Positive	TG 51:4; [M+NH4]+; TG(16:1/17:0/18:3)	826.634
Positive	TG 51:4; [M+NH4]+; TG(16:1/17:1/18:2)	826.7076
Positive	TG 51:5; [M+NH4]+; TG(16:1/17:1/18:3)	822.548
Positive	TG 51:5; [M+NH4]+; TG(16:1/17:2/18:2)	820.6012
Positive	TG 51:6; [M+NH4]+; TG(16:1/17:2/18:3)	820.5443
Positive	TG 52:0; [M+Na]+; TG(16:0/16:0/20:0)	816.5794
Positive	TG 52:0; [M+Na]+; TG(16:0/18:0/18:0)	838.1861
Positive	TG 52:0; [M+NH4]+; TG(16:0/16:0/20:0)	868.6768
Positive	TG 52:0; [M+NH4]+; TG(16:0/17:0/19:0)	868.6756
Positive	TG 52:0; [M+NH4]+; TG(16:0/18:0/18:0)	868.6776
Positive	TG 52:0; [M+NH4]+; TG(17:0/17:0/18:0)	844.6529
Positive	TG 52:1; [M+Na]+; TG(16:0/18:0/18:1)	844.679
Positive	TG 52:1; [M+NH4]+; TG(16:0/16:0/20:1)	866.668
Positive	TG 52:1; [M+NH4]+; TG(16:0/16:1/20:0)	866.6549
Positive	TG 52:1; [M+NH4]+; TG(16:0/17:1/19:0)	866.6586
Positive	TG 52:1; [M+NH4]+; TG(16:0/18:0/18:1)	866.6616
Positive	TG 52:1; [M+NH4]+; TG(17:0/17:0/18:1)	866.6662
Positive	TG 52:2; [M+Na]+; TG(16:0/18:0/18:2)	826.5373
Positive	TG 52:2; [M+Na]+; TG(16:0/18:1/18:1)	848.5569
Positive	TG 52:2; [M+Na]+; TG(16:1/18:0/18:1)	844.0732
Positive	TG 52:2; [M+NH4]+; TG(14:0/18:1/20:1)	864.5005
Positive	TG 52:2; [M+NH4]+; TG(16:0/16:1/20:1)	864.6469
Positive	TG 52:2; [M+NH4]+; TG(16:0/18:0/18:2)	864.4921
Positive	TG 52:2; [M+NH4]+; TG(16:0/18:1/18:1)	842.0626
Positive	TG 52:2; [M+NH4]+; TG(16:1/17:1/19:0)	862.6285
Positive	TG 52:2; [M+NH4]+; TG(16:1/18:0/18:1)	862.6236
Positive	TG 52:3; [M+Na]+; TG(16:0/18:0/18:3)	838.6425
Positive	TG 52:3; [M+Na]+; TG(16:0/18:1/18:2)	860.6119
Positive	TG 52:3; [M+Na]+; TG(16:1/18:1/18:1)	860.6079
Positive	TG 52:3; [M+NH4]+; TG(16:0/16:1/20:2)	860.6128
Positive	TG 52:3; [M+NH4]+; TG(16:0/18:1/18:2)	860.6148
Positive	TG 52:3; [M+NH4]+; TG(16:1/18:0/18:2)	860.6102
Positive	TG 52:3; [M+NH4]+; TG(16:1/18:1/18:1)	860.6144
Positive	TG 52:3; [M+NH4]+; TG(17:0/17:1/18:2)	860.6262
Positive	TG 52:3; [M+NH4]+; TG(17:1/17:1/18:1)	836.4669

Positive	TG 52:4; [M+Na]+; TG(16:0/18:1/18:3)	836.614
Positive	TG 52:4; [M+Na]+; TG(16:0/18:2/18:2)	836.4628
Positive	TG 52:4; [M+Na]+; TG(16:1/18:1/18:2)	836.6147
Positive	TG 52:4; [M+NH4]+; TG(16:0/16:0/20:4)	836.6055
Positive	TG 52:4; [M+NH4]+; TG(16:0/18:1/18:3)	858.5959
Positive	TG 52:4; [M+NH4]+; TG(16:0/18:2/18:2)	858.5977
Positive	TG 52:4; [M+NH4]+; TG(16:1/18:1/18:2)	834.6745
Positive	TG 52:4; [M+NH4]+; TG(17:1/17:2/18:1)	856.5814
Positive	TG 52:5; [M+Na]+; TG(16:0/16:1/20:4)	856.5894
Positive	TG 52:5; [M+Na]+; TG(16:0/18:2/18:3)	832.6523
Positive	TG 52:5; [M+Na]+; TG(16:1/18:1/18:3)	832.5854
Positive	TG 52:5; [M+Na]+; TG(16:1/18:2/18:2)	832.5842
Positive	TG 52:5; [M+NH4]+; TG(16:0/16:1/20:4)	854.5691
Positive	TG 52:5; [M+NH4]+; TG(16:0/18:2/18:3)	854.575
Positive	TG 52:5; [M+NH4]+; TG(16:1/18:1/18:3)	830.5999
Positive	TG 52:5; [M+NH4]+; TG(16:1/18:2/18:2)	830.4224
Positive	TG 52:5; [M+NH4]+; TG(17:1/17:2/18:2)	830.5663
Positive	TG 52:6; [M+Li]+; TG(16:0/18:3/18:3)	852.549
Positive	TG 52:6; [M+Na]+; TG(16:0/16:1/20:5)	852.5487
Positive	TG 52:6; [M+Na]+; TG(16:0/18:3/18:3)	828.6167
Positive	TG 52:6; [M+Na]+; TG(16:1/18:2/18:3)	828.6261
Positive	TG 52:6; [M+NH4]+; TG(16:0/16:1/20:5)	828.6109
Positive	TG 52:6; [M+NH4]+; TG(16:0/18:3/18:3)	828.5537
Positive	TG 52:6; [M+NH4]+; TG(16:1/16:1/20:4)	858.6061
Positive	TG 52:6; [M+NH4]+; TG(16:1/18:2/18:3)	858.5476
Positive	TG 52:7; [M+NH4]+; TG(16:1/16:1/20:5)	858.5998
Positive	TG 52:7; [M+NH4]+; TG(16:1/18:3/18:3)	858.5992
Positive	TG 53:0; [M+Na]+; TG(16:0/17:0/20:0)	858.6107
Positive	TG 53:0; [M+Na]+; TG(16:0/18:0/19:0)	856.6193
Positive	TG 53:0; [M+Na]+; TG(17:0/18:0/18:0)	854.6438
Positive	TG 53:0; [M+NH4]+; TG(16:0/16:0/21:0)	874.662
Positive	TG 53:0; [M+NH4]+; TG(16:0/17:0/20:0)	874.6649
Positive	TG 53:0; [M+NH4]+; TG(16:0/18:0/19:0)	874.6635
Positive	TG 53:0; [M+NH4]+; TG(17:0/18:0/18:0)	850.6735
Positive	TG 53:1; [M+Na]+; TG(16:0/17:0/20:1)	850.6693
Positive	TG 53:1; [M+Na]+; TG(16:0/18:1/19:0)	850.673
Positive	TG 53:1; [M+Na]+; TG(17:0/18:0/18:1)	850.6732
Positive	TG 53:2; [M+Na]+; TG(16:0/18:2/19:0)	848.6074
Positive	TG 53:2; [M+Na]+; TG(17:0/18:0/18:2)	848.6569
Positive	TG 53:2; [M+Na]+; TG(17:0/18:1/18:1)	896.7058
Positive	TG 53:2; [M+Na]+; TG(17:1/18:0/18:1)	896.7093

Positive	TG 53:2; [M+NH4]+; TG(16:0/17:0/20:2)	896.7091
Positive	TG 53:2; [M+NH4]+; TG(16:0/18:2/19:0)	894.7271
Positive	TG 53:2; [M+NH4]+; TG(16:1/18:1/19:0)	894.6906
Positive	TG 53:2; [M+NH4]+; TG(17:0/17:2/19:0)	894.6858
Positive	TG 53:2; [M+NH4]+; TG(17:0/18:0/18:2)	854.6085
Positive	TG 53:2; [M+NH4]+; TG(17:1/18:0/18:1)	854.6366
Positive	TG 53:3; [M+NH4]+; TG(16:0/17:0/20:3)	876.5394
Positive	TG 53:3; [M+NH4]+; TG(16:1/18:2/19:0)	876.5298
Positive	TG 53:3; [M+NH4]+; TG(17:0/18:1/18:2)	852.5301
Positive	TG 53:3; [M+NH4]+; TG(17:1/18:0/18:2)	892.6777
Positive	TG 53:4; [M+NH4]+; TG(16:0/17:0/20:4)	892.6761
Positive	TG 53:4; [M+NH4]+; TG(16:0/17:1/20:3)	892.7071
Positive	TG 53:4; [M+NH4]+; TG(17:0/18:1/18:3)	868.6727
Positive	TG 53:4; [M+NH4]+; TG(17:0/18:2/18:2)	868.696
Positive	TG 53:4; [M+NH4]+; TG(17:1/18:1/18:2)	868.6804
Positive	TG 53:5; [M+NH4]+; TG(16:0/17:1/20:4)	890.6581
Positive	TG 53:5; [M+NH4]+; TG(16:1/17:1/20:3)	866.7069
Positive	TG 53:5; [M+NH4]+; TG(17:0/18:2/18:3)	866.6529
Positive	TG 53:5; [M+NH4]+; TG(17:1/18:1/18:3)	866.7032
Positive	TG 53:5; [M+NH4]+; TG(17:1/18:2/18:2)	866.7077
Positive	TG 53:6; [M+NH4]+; TG(16:1/17:1/20:4)	888.64
Positive	TG 53:6; [M+NH4]+; TG(17:1/18:2/18:3)	888.6449
Positive	TG 53:6; [M+NH4]+; TG(17:2/18:2/18:2)	864.6468
Positive	TG 53:7; [M+NH4]+; TG(17:1/18:3/18:3)	864.6724
Positive	TG 53:7; [M+NH4]+; TG(17:2/18:2/18:3)	864.6526
Positive	TG 54:0; [M+Li]+; TG(16:0/18:0/20:0)	864.6306
Positive	TG 54:0; [M+Li]+; TG(17:0/18:0/19:0)	864.6542
Positive	TG 54:0; [M+Na]+; TG(16:0/16:0/22:0)	862.6154
Positive	TG 54:0; [M+Na]+; TG(16:0/18:0/20:0)	860.6042
Positive	TG 54:0; [M+Na]+; TG(17:0/18:0/19:0)	858.5888
Positive	TG 54:0; [M+NH4]+; TG(16:0/16:0/22:0)	858.5954
Positive	TG 54:0; [M+NH4]+; TG(16:0/18:0/20:0)	858.6699
Positive	TG 54:0; [M+NH4]+; TG(16:0/19:0/19:0)	856.5813
Positive	TG 54:1; [M+Na]+; TG(16:0/18:1/20:0)	856.5811
Positive	TG 54:1; [M+Na]+; TG(18:0/18:0/18:1)	856.5857
Positive	TG 54:1; [M+NH4]+; TG(16:0/16:1/22:0)	856.5727
Positive	TG 54:1; [M+NH4]+; TG(16:0/17:1/21:0)	856.5808
Positive	TG 54:1; [M+NH4]+; TG(16:0/18:0/20:1)	856.5902
Positive	TG 54:1; [M+NH4]+; TG(16:0/18:1/20:0)	908.7539
Positive	TG 54:1; [M+NH4]+; TG(17:0/18:1/19:0)	908.7553
Positive	TG 54:1; [M+NH4]+; TG(18:0/18:0/18:1)	908.7508

Positive	TG 54:2; [M+Na]+; TG(16:0/18:1/20:1)	880.7215
Positive	TG 54:2; [M+Na]+; TG(18:0/18:0/18:2)	879.5857
Positive	TG 54:2; [M+Na]+; TG(18:0/18:1/18:1)	902.705
Positive	TG 54:2; [M+NH4]+; TG(16:0/16:1/22:1)	902.7029
Positive	TG 54:2; [M+NH4]+; TG(16:0/18:0/20:2)	902.6972
Positive	TG 54:2; [M+NH4]+; TG(16:0/18:1/20:1)	878.693
Positive	TG 54:2; [M+NH4]+; TG(16:0/18:2/20:0)	900.6752
Positive	TG 54:2; [M+NH4]+; TG(16:1/18:0/20:1)	900.6845
Positive	TG 54:2; [M+NH4]+; TG(16:1/18:1/20:0)	876.6863
Positive	TG 54:2; [M+NH4]+; TG(18:0/18:0/18:2)	898.6657
Positive	TG 54:2; [M+NH4]+; TG(18:0/18:1/18:1)	924.7371
Positive	TG 54:3; [M+Na]+; TG(16:0/18:0/20:3)	924.7406
Positive	TG 54:3; [M+Na]+; TG(16:0/18:1/20:2)	924.8425
Positive	TG 54:3; [M+Na]+; TG(18:0/18:1/18:2)	899.6964
Positive	TG 54:3; [M+NH4]+; TG(16:0/18:2/20:1)	922.7264
Positive	TG 54:3; [M+NH4]+; TG(16:1/18:1/20:1)	878.7044
Positive	TG 54:3; [M+NH4]+; TG(18:0/18:1/18:2)	897.7501
Positive	TG 54:4; [M+Na]+; TG(16:0/18:0/20:4)	920.7147
Positive	TG 54:4; [M+Na]+; TG(16:0/18:1/20:3)	920.7039
Positive	TG 54:4; [M+Na]+; TG(16:0/18:2/20:2)	920.7088
Positive	TG 54:4; [M+Na]+; TG(18:0/18:1/18:3)	896.7225
Positive	TG 54:4; [M+Na]+; TG(18:0/18:2/18:2)	896.7368
Positive	TG 54:4; [M+Na]+; TG(18:1/18:1/18:2)	894.6909
Positive	TG 54:4; [M+NH4]+; TG(16:0/18:0/20:4)	894.6881
Positive	TG 54:4; [M+NH4]+; TG(16:0/18:1/20:3)	894.7357
Positive	TG 54:4; [M+NH4]+; TG(16:0/18:2/20:2)	894.7347
Positive	TG 54:4; [M+NH4]+; TG(16:1/18:1/20:2)	894.7377
Positive	TG 54:4; [M+NH4]+; TG(18:0/18:2/18:2)	916.6782
Positive	TG 54:4; [M+NH4]+; TG(18:1/18:1/18:2)	916.6626
Positive	TG 54:5; [M+Na]+; TG(16:0/18:1/20:4)	916.6758
Positive	TG 54:5; [M+Na]+; TG(16:0/18:2/20:3)	916.7441
Positive	TG 54:5; [M+Na]+; TG(16:1/18:1/20:3)	892.6828
Positive	TG 54:5; [M+Na]+; TG(18:1/18:1/18:3)	892.5266
Positive	TG 54:5; [M+Na]+; TG(18:1/18:2/18:2)	914.6615
Positive	TG 54:5; [M+NH4]+; TG(16:0/18:2/20:3)	890.7401
Positive	TG 54:5; [M+NH4]+; TG(16:1/18:1/20:3)	908.7495
Positive	TG 54:5; [M+NH4]+; TG(18:1/18:1/18:3)	884.6133
Positive	TG 54:5; [M+NH4]+; TG(18:1/18:2/18:2)	936.7715
Positive	TG 54:6; [M+Li]+; TG(18:0/18:3/18:3)	908.7534
Positive	TG 54:6; [M+Na]+; TG(16:0/18:1/20:5)	908.7668
Positive	TG 54:6; [M+Na]+; TG(16:0/18:2/20:4)	930.693

Positive	TG 54:6; [M+Na]+; TG(16:1/18:1/20:4)	926.6982
Positive	TG 54:6; [M+Na]+; TG(16:1/18:2/20:3)	928.71
Positive	TG 54:6; [M+Na]+; TG(18:1/18:2/18:3)	950.7522
Positive	TG 54:6; [M+NH4]+; TG(16:0/18:2/20:4)	948.7402
Positive	TG 54:6; [M+NH4]+; TG(16:1/18:1/20:4)	948.7501
Positive	TG 54:6; [M+NH4]+; TG(16:1/18:2/20:3)	946.7178
Positive	TG 54:6; [M+NH4]+; TG(18:1/18:2/18:3)	944.7032
Positive	TG 54:6; [M+NH4]+; TG(18:2/18:2/18:2)	920.5636
Positive	TG 54:7; [M+Na]+; TG(16:0/18:2/20:5)	918.6968
Positive	TG 54:7; [M+Na]+; TG(16:0/18:3/20:4)	918.6841
Positive	TG 54:7; [M+Na]+; TG(16:1/18:2/20:4)	918.6951
Positive	TG 54:7; [M+Na]+; TG(16:1/18:3/20:3)	978.7887
Positive	TG 54:7; [M+Na]+; TG(18:1/18:3/18:3)	1004.8006
Positive	TG 54:7; [M+Na]+; TG(18:2/18:2/18:3)	454.2919
Positive	TG 54:7; [M+NH4]+; TG(16:0/16:1/22:6)	454.2907
Positive	TG 54:7; [M+NH4]+; TG(16:0/18:2/20:5)	454.2919
Positive	TG 54:7; [M+NH4]+; TG(16:0/18:3/20:4)	454.2938
Positive	TG 54:7; [M+NH4]+; TG(16:1/18:2/20:4)	454.2898
Positive	TG 54:7; [M+NH4]+; TG(18:1/18:3/18:3)	454.2913
Positive	TG 54:8; [M+Na]+; TG(16:1/16:1/22:6)	454.2932
Positive	TG 54:8; [M+NH4]+; TG(16:1/16:1/22:6)	468.3056
Positive	TG 54:8; [M+NH4]+; TG(16:1/18:2/20:5)	468.3874
Positive	TG 54:8; [M+NH4]+; TG(16:1/18:3/20:4)	468.3067
Positive	TG 54:8; [M+NH4]+; TG(18:2/18:3/18:3)	468.3092
Positive	TG 55:0; [M+Li]+; TG(16:0/18:0/21:0)	482.3269
Positive	TG 55:0; [M+NH4]+; TG(17:0/18:0/20:0)	482.3229
Positive	TG 55:1; [M+Li]+; TG(18:0/18:1/19:0)	482.3234
Positive	TG 55:1; [M+Na]+; TG(16:0/18:1/21:0)	482.4045
Positive	TG 55:1; [M+Na]+; TG(18:0/18:1/19:0)	480.3416
Positive	TG 55:1; [M+NH4]+; TG(16:0/18:1/21:0)	480.3043
Positive	TG 55:1; [M+NH4]+; TG(16:0/19:0/20:1)	510.3527
Positive	TG 55:1; [M+NH4]+; TG(17:0/18:0/20:1)	508.3009
Positive	TG 55:1; [M+NH4]+; TG(17:0/18:1/20:0)	508.3739
Positive	TG 55:1; [M+NH4]+; TG(17:1/18:0/20:0)	508.3045
Positive	TG 55:2; [M+Na]+; TG(16:0/18:2/21:0)	508.302
Positive	TG 55:2; [M+Na]+; TG(16:1/18:1/21:0)	524.3621
Positive	TG 55:2; [M+Na]+; TG(17:0/18:1/20:1)	538.3867
Positive	TG 55:2; [M+Na]+; TG(18:0/18:2/19:0)	538.3819
Positive	TG 55:2; [M+Na]+; TG(18:1/18:1/19:0)	538.3841
Positive	TG 55:2; [M+NH4]+; TG(17:0/18:2/20:0)	538.3841
Positive	TG 55:2; [M+NH4]+; TG(17:1/18:1/20:0)	538.3864

Positive	TG 55:2; [M+NH4]+; TG(18:0/18:2/19:0)	552.4019
Positive	TG 55:2; [M+NH4]+; TG(18:1/18:1/19:0)	566.4123
Positive	TG 55:3; [M+Na]+; TG(18:1/18:2/19:0)	566.4168
Positive	TG 55:3; [M+NH4]+; TG(16:1/18:2/21:0)	566.4148
Positive	TG 55:3; [M+NH4]+; TG(17:0/18:0/20:3)	564.3938
Positive	TG 55:3; [M+NH4]+; TG(17:0/18:2/20:1)	564.4006
Positive	TG 55:3; [M+NH4]+; TG(17:1/18:1/20:1)	558.3174
Positive	TG 55:3; [M+NH4]+; TG(18:1/18:2/19:0)	580.3557
Positive	TG 55:4; [M+NH4]+; TG(17:0/18:1/20:3)	650.4712
Positive	TG 55:4; [M+NH4]+; TG(17:1/18:0/20:3)	664.5145
Positive	TG 55:4; [M+NH4]+; TG(17:1/18:1/20:2)	664.5008
Positive	TG 55:4; [M+NH4]+; TG(18:2/18:2/19:0)	664.4969
Positive	TG 55:5; [M+NH4]+; TG(16:0/17:1/22:4)	664.5079
Positive	TG 55:5; [M+NH4]+; TG(17:0/18:1/20:4)	662.4757
Positive	TG 55:5; [M+NH4]+; TG(17:0/18:2/20:3)	662.4756
Positive	TG 55:5; [M+NH4]+; TG(17:1/18:0/20:4)	678.5077
Positive	TG 55:5; [M+NH4]+; TG(17:1/18:1/20:3)	678.4971
Positive	TG 55:6; [M+Li]+; TG(17:0/18:3/20:3)	678.5082
Positive	TG 55:6; [M+NH4]+; TG(16:0/17:0/22:6)	676.4972
Positive	TG 55:6; [M+NH4]+; TG(17:0/18:2/20:4)	706.5391
Positive	TG 55:6; [M+NH4]+; TG(17:1/18:1/20:4)	706.5344
Positive	TG 55:6; [M+NH4]+; TG(17:1/18:2/20:3)	706.5296
Positive	TG 55:7; [M+NH4]+; TG(16:0/17:1/22:6)	704.5225
Positive	TG 55:7; [M+NH4]+; TG(17:1/18:2/20:4)	704.5235
Positive	TG 56:0; [M+Na]+; TG(16:0/18:0/22:0)	722.2027
Positive	TG 56:0; [M+Na]+; TG(17:0/19:0/20:0)	694.5429
Positive	TG 56:0; [M+NH4]+; TG(16:0/18:0/22:0)	720.5561
Positive	TG 56:0; [M+NH4]+; TG(16:0/20:0/20:0)	720.5534
Positive	TG 56:0; [M+NH4]+; TG(18:0/19:0/19:0)	720.5559
Positive	TG 56:1; [M+Li]+; TG(18:0/18:1/20:0)	718.5398
Positive	TG 56:1; [M+Na]+; TG(16:0/18:0/22:1)	716.2605
Positive	TG 56:1; [M+Na]+; TG(16:0/18:1/22:0)	715.6442
Positive	TG 56:1; [M+Na]+; TG(16:0/20:0/20:1)	715.6496
Positive	TG 56:1; [M+Na]+; TG(18:0/18:1/20:0)	738.5379
Positive	TG 56:1; [M+NH4]+; TG(16:0/18:0/22:1)	714.5136
Positive	TG 56:1; [M+NH4]+; TG(16:0/18:1/22:0)	714.5543
Positive	TG 56:1; [M+NH4]+; TG(16:1/18:0/22:0)	714.5087
Positive	TG 56:1; [M+NH4]+; TG(18:0/18:1/20:0)	712.4902
Positive	TG 56:10; [M+NH4]+; TG(16:1/18:3/22:6)	734.6023
Positive	TG 56:2; [M+Na]+; TG(16:0/18:0/22:2)	734.5647
Positive	TG 56:2; [M+Na]+; TG(16:0/18:1/22:1)	734.604

Positive	TG 56:2; [M+Na]+; TG(16:0/18:2/22:0)	732.6115
Positive	TG 56:2; [M+Na]+; TG(16:1/18:0/22:1)	730.5378
Positive	TG 56:2; [M+Na]+; TG(18:0/18:1/20:1)	730.5407
Positive	TG 56:2; [M+Na]+; TG(18:0/18:2/20:0)	730.538
Positive	TG 56:2; [M+Na]+; TG(18:1/18:1/20:0)	730.5631
Positive	TG 56:2; [M+NH4]+; TG(16:0/18:1/22:1)	730.5386
Positive	TG 56:2; [M+NH4]+; TG(16:0/18:2/22:0)	750.1495
Positive	TG 56:2; [M+NH4]+; TG(16:0/20:1/20:1)	750.1714
Positive	TG 56:2; [M+NH4]+; TG(16:1/18:1/22:0)	726.5036
Positive	TG 56:2; [M+NH4]+; TG(18:0/18:0/20:2)	726.5054
Positive	TG 56:2; [M+NH4]+; TG(18:0/18:1/20:1)	746.5109
Positive	TG 56:2; [M+NH4]+; TG(18:0/18:2/20:0)	746.5741
Positive	TG 56:2; [M+NH4]+; TG(18:1/18:1/20:0)	768.5522
Positive	TG 56:3; [M+Na]+; TG(16:0/18:0/22:3)	744.5596
Positive	TG 56:3; [M+Na]+; TG(18:0/18:0/20:3)	744.5559
Positive	TG 56:3; [M+Na]+; TG(18:0/18:1/20:2)	766.5417
Positive	TG 56:3; [M+Na]+; TG(18:0/18:3/20:0)	766.5348
Positive	TG 56:3; [M+Na]+; TG(18:1/18:1/20:1)	742.5359
Positive	TG 56:3; [M+Na]+; TG(18:1/18:2/20:0)	742.5396
Positive	TG 56:3; [M+NH4]+; TG(16:0/18:0/22:3)	764.1259
Positive	TG 56:3; [M+NH4]+; TG(16:0/18:2/22:1)	764.0992
Positive	TG 56:3; [M+NH4]+; TG(16:0/20:1/20:2)	740.526
Positive	TG 56:3; [M+NH4]+; TG(16:1/18:2/22:0)	738.5213
Positive	TG 56:3; [M+NH4]+; TG(18:0/18:0/20:3)	736.4996
Positive	TG 56:3; [M+NH4]+; TG(18:0/18:1/20:2)	736.4917
Positive	TG 56:3; [M+NH4]+; TG(18:0/18:2/20:1)	758.6064
Positive	TG 56:3; [M+NH4]+; TG(18:1/18:1/20:1)	756.5885
Positive	TG 56:3; [M+NH4]+; TG(18:1/18:2/20:0)	754.5388
Positive	TG 56:4; [M+Na]+; TG(16:0/18:0/22:4)	752.5195
Positive	TG 56:4; [M+Na]+; TG(16:0/18:1/22:3)	774.5382
Positive	TG 56:4; [M+Na]+; TG(16:0/20:1/20:3)	772.5283
Positive	TG 56:4; [M+Na]+; TG(18:0/18:0/20:4)	768.5533
Positive	TG 56:4; [M+Na]+; TG(18:0/18:1/20:3)	790.5385
Positive	TG 56:4; [M+Na]+; TG(18:0/18:2/20:2)	766.9763
Positive	TG 56:4; [M+Na]+; TG(18:1/18:1/20:2)	766.1339
Positive	TG 56:4; [M+Na]+; TG(18:1/18:2/20:1)	764.5803
Positive	TG 56:4; [M+NH4]+; TG(16:0/18:1/22:3)	764.1155
Positive	TG 56:4; [M+NH4]+; TG(16:0/20:1/20:3)	764.5845
Positive	TG 56:4; [M+NH4]+; TG(17:0/17:0/22:4)	762.501
Positive	TG 56:4; [M+NH4]+; TG(18:0/18:0/20:4)	786.6357
Positive	TG 56:4; [M+NH4]+; TG(18:0/18:1/20:3)	808.7037

Positive	TG 56:4; [M+NH4]+; TG(18:0/18:2/20:2)	808.6176
Positive	TG 56:4; [M+NH4]+; TG(18:1/18:2/20:1)	784.6857
Positive	TG 56:5; [M+Li]+; TG(16:0/18:0/22:5)	778.5429
Positive	TG 56:5; [M+Na]+; TG(16:0/18:0/22:5)	778.5005
Positive	TG 56:5; [M+Na]+; TG(16:0/18:1/22:4)	804.6453
Positive	TG 56:5; [M+Na]+; TG(18:0/18:1/20:4)	824.6149
Positive	TG 56:5; [M+Na]+; TG(18:0/18:2/20:3)	824.6166
Positive	TG 56:5; [M+Na]+; TG(18:1/18:1/20:3)	800.6142
Positive	TG 56:5; [M+Na]+; TG(18:1/18:2/20:2)	822.5955
Positive	TG 56:5; [M+NH4]+; TG(16:0/18:0/22:5)	792.5567
Positive	TG 56:5; [M+NH4]+; TG(16:0/18:1/22:4)	790.5403
Positive	TG 56:5; [M+NH4]+; TG(16:0/20:2/20:3)	786.5686
Positive	TG 56:5; [M+NH4]+; TG(17:0/17:0/22:5)	816.6377
Positive	TG 56:5; [M+NH4]+; TG(18:0/18:1/20:4)	814.6329
Positive	TG 56:5; [M+NH4]+; TG(18:1/18:1/20:3)	834.6387
Positive	TG 56:5; [M+NH4]+; TG(18:1/18:2/20:2)	834.6325
Positive	TG 56:5; [M+NH4]+; TG(18:1/18:3/20:1)	832.6105
Positive	TG 56:5; [M+NH4]+; TG(18:2/18:2/20:1)	832.6179
Positive	TG 56:6; [M+Li]+; TG(16:0/18:1/22:5)	852.6469
Positive	TG 56:6; [M+Li]+; TG(16:0/18:3/22:3)	828.6503
Positive	TG 56:6; [M+Li]+; TG(16:0/20:3/20:3)	828.6474
Positive	TG 56:6; [M+Li]+; TG(18:0/18:3/20:3)	828.6462
Positive	TG 56:6; [M+Na]+; TG(16:0/18:1/22:5)	850.6469
Positive	TG 56:6; [M+Na]+; TG(16:0/18:2/22:4)	850.6317
Positive	TG 56:6; [M+Na]+; TG(16:0/20:3/20:3)	850.6296
Positive	TG 56:6; [M+Na]+; TG(16:1/18:1/22:4)	826.6461
Positive	TG 56:6; [M+Na]+; TG(18:0/18:1/20:5)	848.6024
Positive	TG 56:6; [M+Na]+; TG(18:0/18:2/20:4)	856.719
Positive	TG 56:6; [M+Na]+; TG(18:1/18:1/20:4)	852.6857
Positive	TG 56:6; [M+Na]+; TG(18:1/18:2/20:3)	852.6561
Positive	TG 56:6; [M+NH4]+; TG(16:0/18:0/22:6)	874.6295
Positive	TG 56:6; [M+NH4]+; TG(16:0/18:1/22:5)	850.6373
Positive	TG 56:6; [M+NH4]+; TG(16:0/18:2/22:4)	848.6196
Positive	TG 56:6; [M+NH4]+; TG(16:0/20:3/20:3)	880.6948
Positive	TG 56:6; [M+NH4]+; TG(16:1/18:1/22:4)	544.3355
Positive	TG 56:6; [M+NH4]+; TG(18:0/18:2/20:4)	572.3705
Positive	TG 56:6; [M+NH4]+; TG(18:1/18:1/20:4)	600.3934
Positive	TG 56:6; [M+NH4]+; TG(18:1/18:2/20:3)	656.4566
Positive	TG 56:7; [M+Li]+; TG(18:0/18:3/20:4)	712.4948
Positive	TG 56:7; [M+Na]+; TG(16:0/18:1/22:6)	738.1693
Positive	TG 56:7; [M+Na]+; TG(16:0/18:2/22:5)	766.5337

Positive	TG 56:7; [M+Na]+; TG(16:0/20:3/20:4)	764.127
Positive	TG 56:7; [M+Na]+; TG(16:1/18:1/22:5)	781.7048
Positive	TG 56:7; [M+Na]+; TG(18:0/18:2/20:5)	796.5746
Positive	TG 56:7; [M+Na]+; TG(18:1/18:2/20:4)	794.5991
Positive	TG 56:7; [M+Na]+; TG(18:1/18:3/20:3)	788.5587
Positive	TG 56:7; [M+Na]+; TG(18:2/18:2/20:3)	811.0856
Positive	TG 56:7; [M+NH4]+; TG(16:0/18:1/22:6)	824.6493
Positive	TG 56:7; [M+NH4]+; TG(16:0/18:2/22:5)	822.6421
Positive	TG 56:7; [M+NH4]+; TG(16:0/20:3/20:4)	822.675
Positive	TG 56:7; [M+NH4]+; TG(16:1/18:1/22:5)	822.6841
Positive	TG 56:7; [M+NH4]+; TG(18:1/18:2/20:4)	816.593
Positive	TG 56:8; [M+Li]+; TG(18:0/18:3/20:5)	838.6319
Positive	TG 56:8; [M+Na]+; TG(16:0/20:4/20:4)	852.7303
Positive	TG 56:8; [M+Na]+; TG(16:1/18:1/22:6)	850.6239
Positive	TG 56:8; [M+Na]+; TG(16:1/18:2/22:5)	878.7003
Positive	TG 56:8; [M+Na]+; TG(18:1/18:2/20:5)	876.6884
Positive	TG 56:8; [M+Na]+; TG(18:1/18:3/20:4)	872.7188
Positive	TG 56:8; [M+Na]+; TG(18:2/18:2/20:4)	894.7334
Positive	TG 56:8; [M+NH4]+; TG(16:0/20:4/20:4)	906.7321
Positive	TG 56:8; [M+NH4]+; TG(16:1/18:1/22:6)	900.6853
Positive	TG 56:8; [M+NH4]+; TG(16:1/18:2/22:5)	648.4953
Positive	TG 56:8; [M+NH4]+; TG(18:1/18:2/20:5)	662.5134
Positive	TG 56:8; [M+NH4]+; TG(18:1/18:3/20:4)	696.4988
Positive	TG 56:8; [M+NH4]+; TG(18:2/18:2/20:4)	696.4946
Positive	TG 56:8; [M+NH4]+; TG(18:2/18:3/20:3)	726.5385
Positive	TG 56:9; [M+NH4]+; TG(16:0/18:3/22:6)	702.5452
Positive	TG 56:9; [M+NH4]+; TG(16:0/20:4/20:5)	724.5226
Positive	TG 56:9; [M+NH4]+; TG(16:1/18:2/22:6)	722.2287
Positive	TG 56:9; [M+NH4]+; TG(16:1/20:4/20:4)	698.5116
Positive	TG 56:9; [M+NH4]+; TG(18:1/18:3/20:5)	752.558
Positive	TG 56:9; [M+NH4]+; TG(18:2/18:2/20:5)	728.5958
Positive	TG 56:9; [M+NH4]+; TG(18:2/18:3/20:4)	728.2694
Positive	TG 57:0; [M+Na]+; TG(17:0/19:0/21:0)	750.1585
Positive	TG 57:1; [M+NH4]+; TG(18:0/18:1/21:0)	750.1687
Positive	TG 57:2; [M+NH4]+; TG(17:0/18:2/22:0)	727.5839
Positive	TG 57:2; [M+NH4]+; TG(18:0/18:2/21:0)	726.5428
Positive	TG 57:2; [M+NH4]+; TG(18:1/18:1/21:0)	724.5285
Positive	TG 57:2; [M+NH4]+; TG(18:1/19:0/20:1)	722.5108
Positive	TG 57:2; [M+NH4]+; TG(18:2/19:0/20:0)	758.6046
Positive	TG 57:3; [M+Li]+; TG(18:1/18:2/21:0)	780.5864
Positive	TG 57:3; [M+Na]+; TG(18:1/18:2/21:0)	780.5862

Positive	TG 57:3; [M+NH4]+; TG(17:0/18:0/22:3)	780.5886
Positive	TG 57:3; [M+NH4]+; TG(18:0/19:0/20:3)	778.5742
Positive	TG 57:3; [M+NH4]+; TG(18:1/18:2/21:0)	754.5743
Positive	TG 57:3; [M+NH4]+; TG(18:2/19:0/20:1)	776.5562
Positive	TG 57:4; [M+Li]+; TG(16:0/20:4/21:0)	752.5611
Positive	TG 57:4; [M+NH4]+; TG(17:0/18:0/22:4)	752.5597
Positive	TG 57:4; [M+NH4]+; TG(17:0/18:1/22:3)	774.5434
Positive	TG 57:4; [M+NH4]+; TG(18:1/19:0/20:3)	750.5438
Positive	TG 57:4; [M+NH4]+; TG(18:2/18:2/21:0)	750.541
Positive	TG 57:4; [M+NH4]+; TG(18:2/19:0/20:2)	748.527
Positive	TG 57:5; [M+NH4]+; TG(17:0/18:0/22:5)	808.62
Positive	TG 57:5; [M+NH4]+; TG(17:0/18:1/22:4)	808.6154
Positive	TG 57:5; [M+NH4]+; TG(17:0/20:1/20:4)	806.6032
Positive	TG 57:5; [M+NH4]+; TG(17:1/18:0/22:4)	782.6037
Positive	TG 57:6; [M+NH4]+; TG(17:0/18:1/22:5)	804.5819
Positive	TG 57:6; [M+NH4]+; TG(17:0/18:2/22:4)	780.5919
Positive	TG 57:6; [M+NH4]+; TG(17:1/18:1/22:4)	780.5928
Positive	TG 57:7; [M+NH4]+; TG(17:0/18:2/22:5)	802.573
Positive	TG 57:7; [M+NH4]+; TG(17:1/18:1/22:5)	802.5711
Positive	TG 57:8; [M+NH4]+; TG(17:0/18:2/22:6)	778.5717
Positive	TG 57:8; [M+NH4]+; TG(17:1/20:3/20:4)	776.5592
Positive	TG 57:9; [M+NH4]+; TG(17:1/18:2/22:6)	836.6519
Positive	TG 58:1; [M+NH4]+; TG(16:0/20:1/22:0)	834.6354
Positive	TG 58:10; [M+NH4]+; TG(16:0/20:4/22:6)	834.632
Positive	TG 58:10; [M+NH4]+; TG(18:2/18:2/22:6)	808.6238
Positive	TG 58:10; [M+NH4]+; TG(18:2/18:3/22:5)	808.6603
Positive	TG 58:10; [M+NH4]+; TG(18:2/20:4/20:4)	804.5914
Positive	TG 58:2; [M+Na]+; TG(18:1/18:1/22:0)	842.6879
Positive	TG 58:2; [M+NH4]+; TG(16:0/20:1/22:1)	790.1986
Positive	TG 58:2; [M+NH4]+; TG(16:0/20:2/22:0)	830.4912
Positive	TG 58:2; [M+NH4]+; TG(16:1/20:1/22:0)	862.626
Positive	TG 58:2; [M+NH4]+; TG(18:0/18:2/22:0)	543.3947
Positive	TG 58:2; [M+NH4]+; TG(18:1/18:1/22:0)	606.4297
Positive	TG 58:2; [M+NH4]+; TG(18:1/20:0/20:1)	635.4
Positive	TG 58:3; [M+Na]+; TG(18:1/18:1/22:1)	678.4765
Positive	TG 58:3; [M+Na]+; TG(18:1/18:2/22:0)	699.5334
Positive	TG 58:3; [M+NH4]+; TG(18:0/18:0/22:3)	673.4045
Positive	TG 58:3; [M+NH4]+; TG(18:0/18:1/22:2)	713.4994
Positive	TG 58:3; [M+NH4]+; TG(18:0/18:2/22:1)	687.4782
Positive	TG 58:3; [M+NH4]+; TG(18:0/20:0/20:3)	727.5742
Positive	TG 58:3; [M+NH4]+; TG(18:1/18:2/22:0)	741.5437

Positive	TG 58:3; [M+NH4]+; TG(18:1/20:1/20:1)	715.4687
Positive	TG 58:3; [M+NH4]+; TG(18:2/20:0/20:1)	732.457
Positive	TG 58:4; [M+Na]+; TG(18:0/18:0/22:4)	731.4976
Positive	TG 58:4; [M+Na]+; TG(18:0/18:1/22:3)	751.571
Positive	TG 58:4; [M+Na]+; TG(18:0/20:1/20:3)	748.491
Positive	TG 58:4; [M+Na]+; TG(18:1/18:2/22:1)	744.4817
Positive	TG 58:4; [M+NH4]+; TG(18:0/18:0/22:4)	743.6762
Positive	TG 58:4; [M+NH4]+; TG(18:0/18:1/22:3)	783.6283
Positive	TG 58:4; [M+NH4]+; TG(18:0/18:2/22:2)	758.5566
Positive	TG 58:4; [M+NH4]+; TG(18:0/20:0/20:4)	781.6474
Positive	TG 58:4; [M+NH4]+; TG(18:0/20:1/20:3)	758.512
Positive	TG 58:4; [M+NH4]+; TG(18:1/18:1/22:2)	753.4713
Positive	TG 58:4; [M+NH4]+; TG(18:1/18:2/22:1)	751.6272
Positive	TG 58:4; [M+NH4]+; TG(18:1/20:0/20:3)	775.6329
Positive	TG 58:4; [M+NH4]+; TG(18:1/20:1/20:2)	797.6565
Positive	TG 58:4; [M+NH4]+; TG(18:2/18:2/22:0)	773.6769
Positive	TG 58:5; [M+Li]+; TG(19:0/19:0/20:5)	795.6394
Positive	TG 58:5; [M+Na]+; TG(18:0/18:1/22:4)	771.7046
Positive	TG 58:5; [M+Na]+; TG(18:0/20:1/20:4)	767.4053
Positive	TG 58:5; [M+Na]+; TG(18:0/20:2/20:3)	789.6556
Positive	TG 58:5; [M+Na]+; TG(18:1/20:1/20:3)	788.5906
Positive	TG 58:5; [M+NH4]+; TG(16:0/20:1/22:4)	811.6663
Positive	TG 58:5; [M+NH4]+; TG(18:0/18:0/22:5)	786.5731
Positive	TG 58:5; [M+NH4]+; TG(18:0/18:1/22:4)	809.6536
Positive	TG 58:5; [M+NH4]+; TG(18:0/20:1/20:4)	807.6284
Positive	TG 58:5; [M+NH4]+; TG(18:0/20:2/20:3)	781.6402
Positive	TG 58:5; [M+NH4]+; TG(18:1/18:1/22:3)	779.1081
Positive	TG 58:5; [M+NH4]+; TG(18:1/18:2/22:2)	804.0091
Positive	TG 58:5; [M+NH4]+; TG(18:1/20:0/20:4)	825.6822
Positive	TG 58:5; [M+NH4]+; TG(18:2/20:1/20:2)	800.5741
Positive	TG 58:6; [M+Li]+; TG(16:0/20:3/22:3)	823.6701
Positive	TG 58:6; [M+Na]+; TG(18:0/18:1/22:5)	800.7247
Positive	TG 58:6; [M+Na]+; TG(18:0/18:2/22:4)	821.6546
Positive	TG 58:6; [M+Na]+; TG(18:0/20:2/20:4)	792.6197
Positive	TG 58:6; [M+Na]+; TG(18:1/20:1/20:4)	818.5786
Positive	TG 58:6; [M+NH4]+; TG(16:0/20:0/22:6)	839.6948
Positive	TG 58:6; [M+NH4]+; TG(18:0/18:0/22:6)	816.5911
Positive	TG 58:6; [M+NH4]+; TG(18:0/18:1/22:5)	837.6801
Positive	TG 58:6; [M+NH4]+; TG(18:0/18:2/22:4)	812.5855
Positive	TG 58:6; [M+NH4]+; TG(18:0/20:2/20:4)	835.6688
Positive	TG 58:6; [M+NH4]+; TG(18:0/20:3/20:3)	809.1058

Positive	TG 58:6; [M+NH4]+; TG(18:1/18:1/22:4)	807.6429
Positive	TG 58:6; [M+NH4]+; TG(18:1/18:2/22:3)	831.9468
Positive	TG 58:6; [M+NH4]+; TG(18:1/20:1/20:4)	853.7111
Positive	TG 58:7; [M+Li]+; TG(18:0/20:3/20:4)	830.4241
Positive	TG 58:7; [M+Na]+; TG(16:0/20:3/22:4)	851.6959
Positive	TG 58:7; [M+Na]+; TG(18:0/18:2/22:5)	827.6738
Positive	TG 58:7; [M+Na]+; TG(18:0/20:3/20:4)	849.6849
Positive	TG 58:7; [M+Na]+; TG(18:1/18:1/22:5)	823.4714
Positive	TG 58:7; [M+Na]+; TG(18:1/18:2/22:4)	820.6669
Positive	TG 58:7; [M+Na]+; TG(18:1/20:2/20:4)	846.542
Positive	TG 58:7; [M+Na]+; TG(18:2/20:1/20:4)	867.7314
Positive	TG 58:7; [M+NH4]+; TG(18:0/18:1/22:6)	843.5261
Positive	TG 58:7; [M+NH4]+; TG(18:0/18:2/22:5)	865.7139
Positive	TG 58:7; [M+NH4]+; TG(18:0/20:3/20:4)	841.6773
Positive	TG 58:7; [M+NH4]+; TG(18:1/18:1/22:5)	863.6984
Positive	TG 58:7; [M+NH4]+; TG(18:1/18:2/22:4)	858.7114
Positive	TG 58:7; [M+NH4]+; TG(18:1/20:2/20:4)	881.7131
Positive	TG 58:7; [M+NH4]+; TG(18:1/20:3/20:3)	856.6004
Positive	TG 58:7; [M+NH4]+; TG(18:2/20:1/20:4)	879.7313
Positive	TG 58:8; [M+Li]+; TG(18:1/18:2/22:5)	856.5824
Positive	TG 58:8; [M+Li]+; TG(18:1/20:3/20:4)	717.5983
Positive	TG 58:8; [M+Na]+; TG(16:0/20:3/22:5)	712.6429
Positive	TG 58:8; [M+Na]+; TG(16:0/20:4/22:4)	710.7023
Positive	TG 58:8; [M+Na]+; TG(18:0/18:2/22:6)	745.6294
Positive	TG 58:8; [M+Na]+; TG(18:0/20:4/20:4)	740.6751
Positive	TG 58:8; [M+Na]+; TG(18:1/18:1/22:6)	740.6776
Positive	TG 58:8; [M+Na]+; TG(18:1/18:2/22:5)	738.6587
Positive	TG 58:8; [M+Na]+; TG(18:1/20:3/20:4)	738.7488
Positive	TG 58:8; [M+Na]+; TG(18:2/20:2/20:4)	736.6455
Positive	TG 58:8; [M+NH4]+; TG(16:0/20:4/22:4)	771.6467
Positive	TG 58:8; [M+NH4]+; TG(18:0/18:2/22:6)	771.6466
Positive	TG 58:8; [M+NH4]+; TG(18:0/20:4/20:4)	766.6915
Positive	TG 58:8; [M+NH4]+; TG(18:1/18:1/22:6)	766.7759
Positive	TG 58:8; [M+NH4]+; TG(18:1/18:2/22:5)	769.6317
Positive	TG 58:8; [M+NH4]+; TG(18:1/20:3/20:4)	764.6805
Positive	TG 58:8; [M+NH4]+; TG(18:2/18:2/22:4)	801.7012
Positive	TG 58:8; [M+NH4]+; TG(18:2/20:2/20:4)	801.6935
Positive	TG 58:8; [M+NH4]+; TG(18:2/20:3/20:3)	796.7403
Positive	TG 58:9; [M+Li]+; TG(18:0/20:4/20:5)	796.7412
Positive	TG 58:9; [M+Na]+; TG(18:1/20:4/20:4)	799.6795
Positive	TG 58:9; [M+NH4]+; TG(16:0/20:3/22:6)	794.7239

Positive	TG 58:9; [M+NH4]+; TG(16:0/20:4/22:5)	797.6623
Positive	TG 58:9; [M+NH4]+; TG(18:1/18:2/22:6)	792.7077
Positive	TG 58:9; [M+NH4]+; TG(18:1/18:3/22:5)	795.6776
Positive	TG 58:9; [M+NH4]+; TG(18:1/20:4/20:4)	790.693
Positive	TG 58:9; [M+NH4]+; TG(18:2/18:2/22:5)	829.7266
Positive	TG 58:9; [M+NH4]+; TG(18:2/18:3/22:4)	824.77
Positive	TG 58:9; [M+NH4]+; TG(18:2/20:3/20:4)	811.677
Positive	TG 59:3; [M+Li]+; TG(18:0/20:3/21:0)	827.7087
Positive	TG 59:5; [M+NH4]+; TG(18:1/19:0/22:4)	827.7139
Positive	TG 60:1; [M+NH4]+; TG(18:0/20:1/22:0)	827.7102
Positive	TG 60:1; [M+NH4]+; TG(18:1/20:0/22:0)	822.7554
Positive	TG 60:10; [M+Li]+; TG(18:1/20:4/22:5)	822.755
Positive	TG 60:10; [M+NH4]+; TG(16:0/22:4/22:6)	822.7911
Positive	TG 60:10; [M+NH4]+; TG(18:0/20:4/22:6)	822.7595
Positive	TG 60:10; [M+NH4]+; TG(18:1/20:3/22:6)	825.6935
Positive	TG 60:10; [M+NH4]+; TG(18:1/20:4/22:5)	825.695
Positive	TG 60:10; [M+NH4]+; TG(18:2/20:3/22:5)	820.7372
Positive	TG 60:10; [M+NH4]+; TG(18:2/20:4/22:4)	820.7411
Positive	TG 60:11; [M+NH4]+; TG(16:0/22:5/22:6)	823.6785
Positive	TG 60:11; [M+NH4]+; TG(18:1/20:4/22:6)	818.7252
Positive	TG 60:11; [M+NH4]+; TG(18:2/20:3/22:6)	820.2072
Positive	TG 60:11; [M+NH4]+; TG(18:2/20:4/22:5)	843.7469
Positive	TG 60:11; [M+NH4]+; TG(20:3/20:4/20:4)	838.7834
Positive	TG 60:2; [M+NH4]+; TG(18:1/20:1/22:0)	841.7225
Positive	TG 60:3; [M+NH4]+; TG(18:1/20:1/22:1)	841.7277
Positive	TG 60:3; [M+NH4]+; TG(18:2/20:1/22:0)	836.7715
Positive	TG 60:4; [M+NH4]+; TG(18:1/20:2/22:1)	836.7349
Positive	TG 60:4; [M+NH4]+; TG(18:1/20:3/22:0)	839.7131
Positive	TG 60:5; [M+NH4]+; TG(16:0/22:1/22:4)	834.713
Positive	TG 60:5; [M+NH4]+; TG(18:0/20:1/22:4)	834.7547
Positive	TG 60:5; [M+NH4]+; TG(18:0/20:3/22:2)	834.721
Positive	TG 60:5; [M+NH4]+; TG(18:1/20:0/22:4)	837.6941
Positive	TG 60:5; [M+NH4]+; TG(18:1/20:1/22:3)	832.7419
Positive	TG 60:5; [M+NH4]+; TG(18:1/20:3/22:1)	832.8033
Positive	TG 60:5; [M+NH4]+; TG(18:2/20:2/22:1)	857.7572
Positive	TG 60:5; [M+NH4]+; TG(18:2/20:3/22:0)	852.7993
Positive	TG 60:6; [M+NH4]+; TG(18:0/20:1/22:5)	855.7468
Positive	TG 60:6; [M+NH4]+; TG(18:0/20:2/22:4)	855.7429
Positive	TG 60:6; [M+NH4]+; TG(18:0/20:3/22:3)	850.7872
Positive	TG 60:6; [M+NH4]+; TG(18:0/20:4/22:2)	849.9002
Positive	TG 60:6; [M+NH4]+; TG(18:1/20:1/22:4)	850.7267

Positive	TG 60:6; [M+NH4]+; TG(18:1/20:2/22:3)	850.6941
Positive	TG 60:6; [M+NH4]+; TG(18:1/20:4/22:1)	853.7259
Positive	TG 60:6; [M+NH4]+; TG(18:2/20:0/22:4)	853.6781
Positive	TG 60:6; [M+NH4]+; TG(18:2/20:4/22:0)	848.771
Positive	TG 60:6; [M+NH4]+; TG(20:0/20:2/20:4)	848.7695
Positive	TG 60:7; [M+NH4]+; TG(18:0/20:2/22:5)	848.7955
Positive	TG 60:7; [M+NH4]+; TG(18:0/20:3/22:4)	848.8046
Positive	TG 60:7; [M+NH4]+; TG(18:1/20:1/22:5)	851.7099
Positive	TG 60:7; [M+NH4]+; TG(18:1/20:2/22:4)	851.7113
Positive	TG 60:7; [M+NH4]+; TG(18:1/20:3/22:3)	851.7097
Positive	TG 60:7; [M+NH4]+; TG(18:1/20:4/22:2)	848.088
Positive	TG 60:7; [M+NH4]+; TG(18:2/20:1/22:4)	846.7543
Positive	TG 60:7; [M+NH4]+; TG(20:0/20:3/20:4)	846.7525
Positive	TG 60:7; [M+NH4]+; TG(20:1/20:2/20:4)	846.7883
Positive	TG 60:8; [M+Li]+; TG(18:1/20:4/22:3)	846.7848
Positive	TG 60:8; [M+NH4]+; TG(16:0/22:4/22:4)	849.6967
Positive	TG 60:8; [M+NH4]+; TG(18:0/20:2/22:6)	849.6949
Positive	TG 60:8; [M+NH4]+; TG(18:0/20:3/22:5)	844.7391
Positive	TG 60:8; [M+NH4]+; TG(18:0/20:4/22:4)	844.7388
Positive	TG 60:8; [M+NH4]+; TG(18:1/20:1/22:6)	847.6766
Positive	TG 60:8; [M+NH4]+; TG(18:2/20:2/22:4)	842.7229
Positive	TG 60:9; [M+NH4]+; TG(16:0/22:4/22:5)	871.7743
Positive	TG 60:9; [M+NH4]+; TG(18:0/20:3/22:6)	871.7704
Positive	TG 60:9; [M+NH4]+; TG(18:0/20:4/22:5)	866.8195
Positive	TG 60:9; [M+NH4]+; TG(18:1/20:3/22:5)	866.8642
Positive	TG 60:9; [M+NH4]+; TG(18:1/20:4/22:4)	869.7602
Positive	TG 60:9; [M+NH4]+; TG(18:2/20:1/22:6)	869.7607
Positive	TG 60:9; [M+NH4]+; TG(18:2/20:3/22:4)	869.7599
Positive	TG 61:0; [M+Na]+; TG(19:0/21:0/21:0)	864.8464
Positive	TG 62:2; [M+NH4]+; TG(18:1/22:0/22:1)	864.8029
Positive	TG 62:4; [M+NH4]+; TG(18:2/22:1/22:1)	864.8026
Positive	TG 62:5; [M+NH4]+; TG(18:0/22:1/22:4)	864.7992
Positive	TG 62:5; [M+NH4]+; TG(18:1/22:0/22:4)	867.7385
Positive	TG 62:6; [M+NH4]+; TG(18:0/22:2/22:4)	867.7411
Positive	TG 62:6; [M+NH4]+; TG(18:1/22:1/22:4)	867.7413
**Positive**	**18:1(d7) Lyso PC (Internal standard)**	**588.55789**
**Negative**	**18:1(d7) Lyso PE (Internal standard)**	**485.33785**
Negative	CL 62:0; [M-2H](2-); CL(16:0/14:0/16:0/16:0)	662.4803
Negative	CL 64:3; [M-2H](2-); CL(16:1/14:0/16:1/18:1)	672.4971
Negative	CL 66:2; [M-2H](2-); CL(14:0/16:1/18:0/18:1)	688.4941
Negative	CL 66:3; [M-2H](2-); CL(14:0/18:2/16:1/18:0)	686.4829

Negative	CL 66:4; [M-2H](2-); CL(14:0/18:1/16:1/18:2)	686.475
Negative	CL 66:4; [M-2H](2-); CL(14:0/18:1/18:2/16:1)	686.4765
Negative	CL 66:4; [M-2H](2-); CL(16:1/16:1/16:1/18:1)	686.5114
Negative	CL 68:2; [M-2H](2-); CL(16:0/18:1/16:1/18:0)	701.6476
Negative	CL 68:2; [M-2H](2-); CL(16:1/16:0/16:1/20:0)	700.5213
Negative	CL 68:3; [M-2H](2-); CL(14:0/18:1/18:0/18:2)	699.6027
Negative	CL 68:3; [M-2H](2-); CL(16:0/18:1/16:0/18:2)	701.6151
Negative	CL 68:3; [M-2H](2-); CL(16:0/18:1/16:1/18:1)	701.6187
Negative	CL 68:3; [M-2H](2-); CL(16:1/16:1/18:1/18:0)	700.6346
Negative	CL 68:3; [M-2H](2-); CL(18:1/16:0/18:2/16:0)	700.4896
Negative	CL 68:4; [M-2H](2-); CL(16:0/16:1/18:1/18:2)	700.6097
Negative	CL 68:4; [M-2H](2-); CL(16:1/16:0/18:2/18:1)	700.4858
Negative	CL 68:4; [M-2H](2-); CL(16:1/18:1/18:2/16:0)	700.4923
Negative	CL 70:0; [M-2H](2-); CL(18:0/16:0/18:0/18:0)	718.5397
Negative	CL 70:3; [M-2H](2-); CL(16:0/16:0/18:1/20:2)	714.507
Negative	CL 70:3; [M-2H](2-); CL(16:0/18:0/18:1/18:2)	714.6532
Negative	CL 70:3; [M-2H](2-); CL(16:0/18:1/18:0/18:2)	713.4899
Negative	CL 70:3; [M-2H](2-); CL(18:1/16:0/18:1/18:1)	714.504
Negative	CL 70:4; [M-2H](2-); CL(16:0/18:1/18:1/18:2)	713.4893
Negative	CL 70:4; [M-2H](2-); CL(16:1/18:1/18:1/18:1)	713.5001
Negative	CL 70:4; [M-2H](2-); CL(16:1/18:1/18:2/18:0)	714.6548
Negative	CL 70:4; [M-2H](2-); CL(18:0/16:1/18:1/18:2)	713.6257
Negative	CL 70:4; [M-2H](2-); CL(18:1/16:0/18:2/18:1)	713.4925
Negative	CL 70:4; [M-2H](2-); CL(18:1/16:1/18:1/18:1)	713.5011
Negative	CL 70:4; [M-2H](2-); CL(18:1/16:1/18:2/18:0)	713.6249
Negative	CL 70:5; [M-2H](2-); CL(16:1/18:2/18:1/18:1)	712.4915
Negative	CL 70:5; [M-2H](2-); CL(18:1/16:1/18:1/18:2)	712.489
Negative	CL 70:5; [M-2H](2-); CL(18:1/18:1/18:2/16:1)	712.4916
Negative	CL 70:5; [M-2H](2-); CL(18:1/18:2/18:2/16:0)	712.4867
Negative	CL 70:5; [M-2H](2-); CL(18:2/16:0/18:2/18:1)	713.4968
Negative	CL 70:6; [M-2H](2-); CL(16:0/18:1/18:2/18:3)	712.4935
Negative	CL 70:6; [M-2H](2-); CL(16:0/18:2/18:3/18:1)	712.6361
Negative	CL 70:6; [M-2H](2-); CL(16:0/18:3/18:1/18:2)	712.4966
Negative	CL 70:6; [M-2H](2-); CL(16:1/18:2/18:2/18:1)	712.6409
Negative	CL 72:0; [M-2H](2-); CL(16:0/20:0/18:0/18:0)	730.6129
Negative	CL 72:0; [M-2H](2-); CL(18:0/16:0/18:0/20:0)	732.5856
Negative	CL 72:1; [M-2H](2-); CL(18:0/18:0/18:0/18:1)	729.5586
Negative	CL 72:1; [M-2H](2-); CL(18:0/18:1/20:0/16:0)	731.5702
Negative	CL 72:10; [M-2H](2-); CL(16:1/20:4/18:2/18:3)	722.5125
Negative	CL 72:10; [M-2H](2-); CL(18:2/18:3/20:4/16:1)	720.4983
Negative	CL 72:2; [M-2H](2-); CL(16:0/18:0/18:1/20:1)	730.571

Negative	CL 72:3; [M-2H](2-); CL(16:0/18:1/18:2/20:0)	728.5271
Negative	CL 72:3; [M-2H](2-); CL(16:0/18:2/18:1/20:0)	728.5204
Negative	CL 72:3; [M-2H](2-); CL(18:0/18:1/18:0/18:2)	728.5739
Negative	CL 72:3; [M-2H](2-); CL(18:1/16:0/18:1/20:1)	728.584
Negative	CL 72:3; [M-2H](2-); CL(18:1/18:0/18:2/18:0)	728.5786
Negative	CL 72:5; [M-2H](2-); CL(16:0/18:2/18:1/20:2)	726.5894
Negative	CL 72:5; [M-2H](2-); CL(18:1/18:0/18:2/18:2)	726.6546
Negative	CL 72:6; [M-2H](2-); CL(16:0/18:1/18:2/20:3)	726.5453
Negative	CL 72:6; [M-2H](2-); CL(16:0/20:3/18:1/18:2)	726.5614
Negative	CL 72:6; [M-2H](2-); CL(16:1/18:1/18:2/20:2)	726.5564
Negative	CL 72:6; [M-2H](2-); CL(18:1/18:1/18:2/18:2)	725.4927
Negative	CL 72:6; [M-2H](2-); CL(18:1/18:2/18:1/18:2)	725.4943
Negative	lysoPE 15:1; [M-H]-; PE(15:1(9Z)/0:0)	436.2828
Negative	lysoPE 16:0; [M-H]-; PE(16:0/0:0)	452.2828
Negative	lysoPE 16:1; [M-H]-; PE(16:1(7Z)/0:0)	450.2951
Negative	lysoPE 17:1; [M-H]-; PE(17:1(9Z)/0:0)	464.3158
Negative	PC 17:0; [M+HCOO]-; GPCho(2:0/15:0)	568.532
Negative	PC 18:0; [M+HCOO]-; GPCho(2:0/16:0)	581.3689
Negative	PC 18:1; [M+HCOO]-; GPCho(2:0/16:1(7Z))	580.5316
Negative	PC 19:0; [M+HCOO]-; GPCho(2:0/17:0)	596.5256
Negative	PC 19:0; [M-CH3]-; GPCho(3:0/16:0)	536.5064
Negative	PC 20:0; [M+HCOO]-; GPCho(2:0/18:0)	610.5765
Negative	PC 21:0; [M+HCOO]-; GPCho(2:0/19:0)	623.4922
Negative	PC 22:0; [M+HCOO]-; GPCho(2:0/20:0)	638.6075
Negative	PC 22:1; [M+HCOO]-; GPCho(2:0/20:1(11E))	635.5227
Negative	PC 22:5; [M+HCOO]-; GPCho(2:0/20:5(5Z,8Z,11Z,14Z,17Z))	627.4763
Negative	PC 23:0; [M+HCOO]-; GPCho(2:0/21:0)	651.6059
Negative	PC 23:0; [M+HCOO]-; GPCho(7:0/16:0)	651.4771
Negative	PC 23:0; [M+HCOO]-; GPCho(8:0/15:0)	651.471
Negative	PC 24:0; [M+HCOO]-; GPCho(11:0/13:0)	665.5357
Negative	PC 24:0; [M+HCOO]-; GPCho(6:0/18:0)	665.5356
Negative	PC 24:0; [M+HCOO]-; GPCho(9:0/15:0)	666.6029
Negative	PC 24:4; [M+HCOO]-; GPCho(4:0/20:4(5E,8E,11E,14E))	658.2384
Negative	PC 24:6; [M+HCOO]-; GPCho(2:0/22:6(4Z,7Z,10Z,13Z,16Z,19Z))	654.5955
Negative	PC 25:0; [M+HCOO]-; GPCho(2:0/23:0)	680.6434
Negative	PC 26:1; [M+HCOO]-; GPCho(2:0/24:1(15Z))	692.5663
Negative	PC 26:4; [M+HCOO]-; GPCho(2:0/24:4(5Z,8Z,11Z,14Z))	686.2701
Negative	PC 26:6; [M+HCOO]-; GPCho(4:0/22:6(4Z,7Z,10Z,13Z,16Z,19Z))	681.5148
Negative	PC 28:0; [M+HCOO]-; GPCho(12:0/16:0)	721.5059
Negative	PC 28:0; [M+HCOO]-; GPCho(14:0/14:0)	722.5075
Negative	PC 28:0; [M+HCOO]-; GPCho(2:0/26:0)	722.6321

Negative	PC 28:0; [M+HCOO]-; GPCho(9:0/19:0)	722.6267
Negative	PC 28:0; [M-Ac-H]-; GPCho(12:0/16:0)	736.5092
Negative	PC 28:0; [M-Ac-H]-; GPCho(14:0/14:0)	736.5094
Negative	PC 29:1; [M-Ac-H]-; GPCho(14:0/15:1(9Z))	748.5481
Negative	PC 30:0; [M+HCOO]-; GPCho(14:0/16:0)	750.5539
Negative	PC 30:0; [M-Ac-H]-; GPCho(14:0/16:0)	764.5456
Negative	PC 30:0; [M-CH3]-; GPCho(14:0/16:0)	690.514
Negative	PC 30:1; [M-Ac-H]-; GPCho(14:0/16:1(7Z))	762.5538
Negative	PC 30:1; [M-CH3]-; GPCho(14:0/16:1(7Z))	688.4999
Negative	PC 30:2; [M-CH3]-; GPCho(14:1(9Z)/16:1(7Z))	686.4726
Negative	PC 30:2; [M-CH3]-; GPCho(15:1(9Z)/15:1(9Z))	686.272
Negative	PC 31:0; [M-Ac-H]-; GPCho(15:0/16:0)	778.5763
Negative	PC 31:1; [M+HCOO]-; GPCho(13:0/18:1(11E))	762.5987
Negative	PC 31:1; [M+HCOO]-; GPCho(15:1(9Z)/16:0)	762.5067
Negative	PC 31:1; [M-Ac-H]-; GPCho(13:0/18:1(11E))	776.5687
Negative	PC 31:3; [M-Ac-H]-; GPCho(11:0/20:3(5Z,8Z,11Z))	772.5626
Negative	PC 32:0; [M+HCOO]-; GPCho(14:0/18:0)	778.5737
Negative	PC 32:0; [M+HCOO]-; GPCho(15:0/17:0)	778.577
Negative	PC 32:0; [M+HCOO]-; GPCho(16:0/16:0)	779.5527
Negative	PC 32:0; [M+HCOO]-; GPCho(6:0/26:0)	778.574
Negative	PC 32:0; [M-Ac-H]-; GPCho(14:0/18:0)	792.6097
Negative	PC 32:0; [M-Ac-H]-; GPCho(16:0/16:0)	792.567
Negative	PC 32:1; [M-Ac-H]-; GPCho(14:0/18:1(11E))	790.5763
Negative	PC 32:1; [M-Ac-H]-; GPCho(16:0/16:1(7Z))	790.6329
Negative	PC 32:1; [M-CH3]-; GPCho(16:0/16:1(9Z))	716.5264
Negative	PC 32:1; [M-CH3]-; GPCho(16:1(7Z)/16:0)	716.5254
Negative	PC 32:2; [M-Ac-H]-; GPCho(14:0/18:2(2E,4E))	788.5459
Negative	PC 32:2; [M-Ac-H]-; GPCho(14:1(9Z)/18:1(11E))	788.523
Negative	PC 32:2; [M-Ac-H]-; GPCho(16:1(7Z)/16:1(7Z))	788.5407
Negative	PC 32:3; [M+HCOO]-; GPCho(14:1(9Z)/18:2(2E,4E))	771.634
Negative	PC 32:3; [M-Ac-H]-; GPCho(14:1(9Z)/18:2(2E,4E))	786.5294
Negative	PC 33:0; [M+HCOO]-; GPCho(16:0/17:0)	792.6132
Negative	PC 33:0; [M-Ac-H]-; GPCho(15:0/18:0)	806.5807
Negative	PC 33:0; [M-Ac-H]-; GPCho(16:0/17:0)	806.6078
Negative	PC 33:1; [M+HCOO]-; GPCho(15:0/18:1(11E))	790.5951
Negative	PC 33:1; [M+HCOO]-; GPCho(15:1(9Z)/18:0)	790.5966
Negative	PC 33:1; [M+HCOO]-; GPCho(16:0/17:1(9Z))	790.5916
Negative	PC 33:1; [M+HCOO]-; GPCho(16:1(7Z)/17:0)	790.597
Negative	PC 33:1; [M-Ac-H]-; GPCho(15:0/18:1(11E))	804.5949
Negative	PC 33:1; [M-Ac-H]-; GPCho(16:0/17:1(9Z))	804.5742
Negative	PC 33:1; [M-CH3]-; GPCho(15:0/18:1(11E))	730.5379

Negative	PC 33:2; [M-CH3]-; GPCho(15:0/18:2(2E,4E))	728.5206
Negative	PC 33:3; [M-Ac-H]-; GPCho(15:0/18:3(6Z,9Z,12Z))	800.5463
Negative	PC 34:0; [M-CH3]-; GPCho(16:0/18:0)	746.5712
Negative	PC 34:1; [M+HCOO]-; GPCho(16:0/18:1(11E))	805.5957
Negative	PC 34:1; [M-Ac-H]-; GPCho(16:0/18:1(11E))	818.5945
Negative	PC 34:1; [M-CH3]-; GPCho(16:0/18:1(11E))	744.5546
Negative	PC 34:2; [M+HCOO]-; GPCho(16:0/18:2(2E,4E))	803.5904
Negative	PC 34:2; [M-Ac-H]-; GPCho(16:0/18:2(2E,4E))	816.6347
Negative	PC 34:2; [M-Ac-H]-; GPCho(16:1(7Z)/18:1(11E))	816.5816
Negative	PC 34:2; [M-CH3]-; GPCho(16:1(7Z)/18:1(11E))	742.5394
Negative	PC 34:4; [M-Ac-H]-; GPCho(14:0/20:4(5E,8E,11E,14E))	812.5499
Negative	PC 34:4; [M-Ac-H]-; GPCho(16:1(7Z)/18:3(6Z,9Z,12Z))	812.5446
Negative	PC 34:5; [M-Ac-H]-; GPCho(14:0/20:5(5Z,8Z,11Z,14Z,17Z))	810.5368
Negative	PC 34:5; [M-Ac-H]-; GPCho(16:1(7Z)/18:4(6Z,9Z,12Z,15Z))	810.5279
Negative	PC 35:1; [M-Ac-H]-; GPCho(17:0/18:1(11E))	832.6136
Negative	PC 35:1; [M-Ac-H]-; GPCho(17:1(9Z)/18:0)	832.6279
Negative	PC 35:2; [M-Ac-H]-; GPCho(17:0/18:2(2E,4E))	830.6113
Negative	PC 35:4; [M-Ac-H]-; GPCho(15:0/20:4(5E,8E,11E,14E))	826.566
Negative	PC 35:4; [M-Ac-H]-; GPCho(17:1(9Z)/18:3(6Z,9Z,12Z))	826.5578
Negative	PC 35:5; [M+HCOO]-; GPCho(15:1(9Z)/20:4(5E,8E,11E,14E))	810.5958
Negative	PC 36:0; [M-CH3]-; GPCho(16:0/20:0)	774.5947
Negative	PC 36:0; [M-CH3]-; GPCho(20:0/16:0)	774.5996
Negative	PC 36:1; [M-Ac-H]-; GPCho(12:0/24:1(15Z))	846.6255
Negative	PC 36:1; [M-Ac-H]-; GPCho(16:0/20:1(11E))	846.6182
Negative	PC 36:1; [M-Ac-H]-; GPCho(18:0/18:1(11E))	846.6233
Negative	PC 36:1; [M-CH3]-; GPCho(18:1(11E)/18:0)	772.5491
Negative	PC 36:2; [M-CH3]-; GPCho(18:0/18:2(2E,4E))	770.5638
Negative	PC 36:2; [M-CH3]-; GPCho(18:1(11E)/18:1(11E))	770.5685
Negative	PC 36:2; [M-CH3]-; GPCho(18:2(2E,4E)/18:0)	770.5767
Negative	PC 36:3; [M+HCOO]-; GPCho(16:0/20:3(5Z,8Z,11Z))	828.576
Negative	PC 36:3; [M-Ac-H]-; GPCho(16:0/20:3(5Z,8Z,11Z))	842.5905
Negative	PC 36:3; [M-Ac-H]-; GPCho(18:1(11E)/18:2(2E,4E))	842.5938
Negative	PC 36:3; [M-CH3]-; GPCho(16:0/20:3(5Z,8Z,11Z))	768.5699
Negative	PC 36:3; [M-CH3]-; GPCho(18:2(2E,4E)/18:1(11E))	768.556
Negative	PC 36:5; [M+HCOO]-; GPCho(16:1(7Z)/20:4(5E,8E,11E,14E))	824.5482
Negative	PC 36:5; [M-Ac-H]-; GPCho(16:0/20:5(5Z,8Z,11Z,14Z,17Z))	838.5697
Negative	PC 36:5; [M-Ac-H]-; GPCho(16:1(7Z)/20:4(5E,8E,11E,14E))	838.5595
Negative	PC 36:5; [M-Ac-H]-; GPCho(18:2(2E,4E)/18:3(6Z,9Z,12Z))	838.56
Negative	PC 36:5; [M-CH3]-; GPCho(16:0/20:5(5Z,8Z,11Z,14Z,17Z))	764.526
Negative	PC 36:5; [M-CH3]-; GPCho(16:1(7Z)/20:4(5E,8E,11E,14E))	764.5293
Negative	PC 36:6; [M-Ac-H]-; GPCho(14:0/22:6(4Z,7Z,10Z,13Z,16Z,19Z))	836.5542

Negative	PC 36:6; [M-Ac-H]-; GPCho(16:1(7Z)/20:5(5Z,8Z,11Z,14Z,17Z))	836.5386
Negative	PC 36:6; [M-Ac-H]-; GPCho(18:3(6Z,9Z,12Z)/18:3(6Z,9Z,12Z))	836.542
Negative	PC 37:1; [M-Ac-H]-; GPCho(17:0/20:1(11E))	860.6377
Negative	PC 37:1; [M-Ac-H]-; GPCho(18:1(11E)/19:0)	860.6414
Negative	PC 37:2; [M-Ac-H]-; GPCho(17:0/20:2(11Z,14Z))	858.6164
Negative	PC 37:2; [M-Ac-H]-; GPCho(17:2(9Z,12Z)/20:0)	858.623
Negative	PC 37:2; [M-Ac-H]-; GPCho(18:2(2E,4E)/19:0)	858.6253
Negative	PC 37:4; [M-Ac-H]-; GPCho(17:0/20:4(5E,8E,11E,14E))	854.5982
Negative	PC 37:4; [M-Ac-H]-; GPCho(17:1(9Z)/20:3(5Z,8Z,11Z))	854.5898
Negative	PC 37:4; [M-CH3]-; GPCho(17:0/20:4(5E,8E,11E,14E))	780.5525
Negative	PC 37:5; [M+HCOO]-; GPCho(17:1(9Z)/20:4(5E,8E,11E,14E))	838.5983
Negative	PC 37:6; [M-Ac-H]-; GPCho(15:0/22:6(4Z,7Z,10Z,13Z,16Z,19Z))	850.5363
Negative	PC 37:6; [M-Ac-H]-; GPCho(17:2(9Z,12Z)/20:4(5E,8E,11E,14E))	850.5598
Negative	PC 38:1; [M-Ac-H]-; GPCho(16:0/22:1(13Z))	874.6557
Negative	PC 38:1; [M-Ac-H]-; GPCho(18:0/20:1(11E))	874.6574
Negative	PC 38:1; [M-CH3]-; GPCho(18:0/20:1(11E))	800.6124
Negative	PC 38:1; [M-CH3]-; GPCho(22:1(13Z)/16:0)	800.6139
Negative	PC 38:2; [M-Ac-H]-; GPCho(16:0/22:2(13Z,16Z))	871.7248
Negative	PC 38:2; [M-Ac-H]-; GPCho(18:0/20:2(11Z,14Z))	872.6437
Negative	PC 38:2; [M-Ac-H]-; GPCho(18:1(11E)/20:1(11E))	872.6327
Negative	PC 38:2; [M-Ac-H]-; GPCho(18:2(2E,4E)/20:0)	872.6329
Negative	PC 38:2; [M-CH3]-; GPCho(18:0/20:2(11Z,14Z))	799.5478
Negative	PC 38:2; [M-CH3]-; GPCho(18:1(11E)/20:1(11E))	798.5997
Negative	PC 38:3; [M-CH3]-; GPCho(18:0/20:3(5Z,8Z,11Z))	796.548
Negative	PC 38:4; [M+HCOO]-; GPCho(18:0/20:4(5E,8E,11E,14E))	854.5942
Negative	PC 38:4; [M-Ac-H]-; GPCho(18:0/20:4(5E,8E,11E,14E))	868.609
Negative	PC 38:4; [M-CH3]-; GPCho(16:0/22:4(7Z,10Z,13Z,16Z))	794.5326
Negative	PC 38:4; [M-CH3]-; GPCho(18:0/20:4(5E,8E,11E,14E))	794.5985
Negative	PC 38:4; [M-CH3]-; GPCho(18:1(11E)/20:3(5Z,8Z,11Z))	794.5715
Negative	PC 38:4; [M-CH3]-; GPCho(20:4(5E,8E,11E,14E)/18:0)	794.5313
Negative	PC 38:6; [M-CH3]-; GPCho(16:0/22:6(4Z,7Z,10Z,13Z,16Z,19Z))	790.5399
Negative	PC 38:6; [M-CH3]-; GPCho(18:2(2E,4E)/20:4(5E,8E,11E,14E))	790.5382
Negative	PC 38:7; [M-Ac-H]-; GPCho(18:3(6Z,9Z,12Z)/20:4(5E,8E,11E,14E))	862.5607
Negative	PC 39:6; [M-Ac-H]-; GPCho(17:0/22:6(4Z,7Z,10Z,13Z,16Z,19Z))	878.5911
Negative	PC 40:1; [M-CH3]-; GPCho(16:0/24:1(15Z))	828.6462
Negative	PC 40:4; [M-Ac-H]-; GPCho(20:0/20:4(5E,8E,11E,14E))	896.6406
Negative	PC 40:5; [M+HCOO]-; GPCho(18:3(6Z,9Z,12Z)/22:2(13Z,16Z))	880.6627
Negative	PC 40:5; [M+HCOO]-; GPCho(20:1(11E)/20:4(5E,8E,11E,14E))	880.6686
Negative	PC 40:5; [M+HCOO]-; GPCho(20:2(11Z,14Z)/20:3(5Z,8Z,11Z))	880.6831
Negative	PC 40:6; [M+HCOO]-; GPCho(18:0/22:6(4Z,7Z,10Z,13Z,16Z,19Z))	879.5682
Negative	PC 40:6; [M-Ac-H]-; GPCho(18:2(2E,4E)/22:4(7Z,10Z,13Z,16Z))	892.6103

Negative	PC 40:6; [M-CH3]-; GPCho(18:1(11E)/22:5(4Z,7Z,10Z,13Z,16Z))	819.5209
Negative	PC 40:7; [M-Ac-H]-; GPCho(18:1(11E)/22:6(4Z,7Z,10Z,13Z,16Z,19Z))	890.5885
Negative	PC 40:7; [M-Ac-H]-; GPCho(18:2(2E,4E)/22:5(4Z,7Z,10Z,13Z,16Z))	890.5956
Negative	PC 40:7; [M-CH3]-; GPCho(18:1(11E)/22:6(4Z,7Z,10Z,13Z,16Z,19Z))	816.5918
Negative	PC 40:8; [M-Ac-H]-; GPCho(18:2(2E,4E)/22:6(4Z,7Z,10Z,13Z,16Z,19Z))	888.5774
Negative	PC 41:0; [M-Ac-H]-; GPCho(16:0/25:0)	918.7222
Negative	PC 41:1; [M-Ac-H]-; GPCho(15:1(9Z)/26:0)	916.7437
Negative	PC 41:1; [M-Ac-H]-; GPCho(18:1(11E)/23:0)	916.7442
Negative	PC 41:6; [M-Ac-H]-; GPCho(19:0/22:6(4Z,7Z,10Z,13Z,16Z,19Z))	906.6077
Negative	PC 42:0; [M-Ac-H]-; GPCho(18:0/24:0)	932.7426
Negative	PC 42:1; [M-Ac-H]-; GPCho(18:1(11E)/24:0)	930.7191
Negative	PC 42:10; [M-Ac-H]-; GPCho(20:4(5E,8E,11E,14E)/22:6(4Z,7Z,10Z,13Z,16Z,19Z))	912.5771
Negative	PC 42:2; [M+HCOO]-; GPCho(16:0/26:2(5E,9Z))	914.7256
Negative	PC 42:2; [M+HCOO]-; GPCho(18:1(11E)/24:1(15Z))	914.7306
Negative	PC 42:6; [M-Ac-H]-; GPCho(18:2(2E,4E)/24:4(5Z,8Z,11Z,14Z))	920.6407
Negative	PC 42:6; [M-Ac-H]-; GPCho(20:2(11Z,14Z)/22:4(7Z,10Z,13Z,16Z))	920.6542
Negative	PC 42:6; [M-Ac-H]-; GPCho(20:4(5E,8E,11E,14E)/22:2(13Z,16Z))	920.6349
Negative	PC 42:7; [M-CH3]-; GPCho(20:3(5Z,8Z,11Z)/22:4(7Z,10Z,13Z,16Z))	845.5397
Negative	PC 42:7; [M-CH3]-; GPCho(22:4(7Z,10Z,13Z,16Z)/20:3(8Z,11Z,14Z))	845.535
Negative	PC 42:9; [M-Ac-H]-; GPCho(20:3(5Z,8Z,11Z)/22:6(4Z,7Z,10Z,13Z,16Z,19Z))	914.6039
Negative	PC 42:9; [M-Ac-H]-; GPCho(20:4(5E,8E,11E,14E)/22:5(4Z,7Z,10Z,13Z,16Z))	914.6035
Negative	PC 44:12; [M-Ac-H]-; GPCho(22:6(4Z,7Z,10Z,13Z,16Z,19Z)/22:6(4Z,7Z,10Z,13Z,16Z,19Z))	936.5775
Negative	PC 45:4; [M-Ac-H]-; GPCho(20:4(5E,8E,11E,14E)/25:0)	966.7917
Negative	CL 68:6; [M-2H](2-); CL(16:0/16:1/18:2/18:3)	696.496
Negative	CL 72:4; [M-2H](2-); CL(16:0/20:3/18:0/18:1)	726.5098
Negative	CL 72:6; [M-2H](2-); CL(18:1/16:0/18:1/20:4)	726.5087
Negative	CL 72:7; [M-2H](2-); CL(18:1/18:2/18:2/18:2)	724.4895
Negative	CL 72:8; [M-2H](2-); CL(16:1/20:4/18:2/18:1)	724.5256
Negative	CL 72:8; [M-2H](2-); CL(18:1/18:2/18:2/18:3)	722.5133
Negative	CL 72:8; [M-2H](2-); CL(18:2/18:1/18:2/18:3)	722.5171
Negative	CL 72:8; [M-2H](2-); CL(18:2/18:2/18:2/18:2)	723.4806
Negative	CL 72:9; [M-2H](2-); CL(16:0/20:4/18:3/18:2)	722.5158
Negative	CL 74:10; [M-2H](2-); CL(16:1/16:0/20:4/22:5)	736.4954
Negative	CL 74:10; [M-2H](2-); CL(20:4/16:0/20:4/18:2)	736.5462
Negative	CL 74:2; [M-2H](2-); CL(18:0/18:1/18:0/20:1)	744.6007
Negative	CL 74:4; [M-2H](2-); CL(18:0/18:1/18:2/20:1)	742.6389
Negative	CL 74:4; [M-2H](2-); CL(18:0/20:1/18:1/18:2)	742.5622
Negative	CL 74:6; [M-2H](2-); CL(18:1/18:1/20:2/18:2)	739.5134
Negative	CL 74:7; [M-2H](2-); CL(18:1/18:1/18:2/20:3)	738.5417
Negative	CL 74:7; [M-2H](2-); CL(18:1/18:2/18:1/20:3)	737.4955
Negative	CL 74:7; [M-2H](2-); CL(18:1/20:2/18:2/18:2)	737.4972

Negative	CL 74:7; [M-2H](2-); CL(18:2/18:2/20:2/18:1)	738.506
Negative	CL 74:8; [M-2H](2-); CL(18:1/18:2/18:2/20:3)	736.4907
Negative	CL 74:8; [M-2H](2-); CL(18:1/18:2/20:3/18:2)	736.4946
Negative	CL 74:8; [M-2H](2-); CL(18:2/18:1/18:2/20:3)	737.4983
Negative	CL 74:8; [M-2H](2-); CL(18:2/18:2/18:2/20:2)	737.4941
Negative	CL 74:8; [M-2H](2-); CL(18:2/18:2/20:2/18:2)	737.4967
Negative	CL 74:9; [M-2H](2-); CL(16:0/20:4/16:0/22:5)	736.4956
Negative	CL 74:9; [M-2H](2-); CL(18:2/16:1/20:3/20:3)	736.4884
Negative	CL 74:9; [M-2H](2-); CL(18:2/18:2/18:2/20:3)	736.4892
Negative	CL 74:9; [M-2H](2-); CL(18:2/20:3/20:3/16:1)	736.4886
Negative	CL 76:10; [M-2H](2-); CL(16:0/18:0/22:6/20:4)	748.5351
Negative	CL 76:10; [M-2H](2-); CL(16:0/20:4/18:0/22:6)	749.5368
Negative	CL 76:10; [M-2H](2-); CL(16:0/22:6/18:0/20:4)	749.5321
Negative	CL 76:10; [M-2H](2-); CL(16:0/22:6/20:4/18:0)	748.5299
Negative	CL 76:10; [M-2H](2-); CL(16:1/22:5/20:4/18:0)	748.555
Negative	CL 76:10; [M-2H](2-); CL(18:0/22:6/20:4/16:0)	749.5305
Negative	CL 76:10; [M-2H](2-); CL(18:2/18:0/18:2/22:6)	748.5491
Negative	CL 76:10; [M-2H](2-); CL(20:3/16:0/20:3/20:4)	749.5439
Negative	CL 76:11; [M-2H](2-); CL(14:0/18:0/22:5/22:6)	748.5259
Negative	CL 76:11; [M-2H](2-); CL(14:0/22:5/18:0/22:6)	748.5546
Negative	CL 76:11; [M-2H](2-); CL(16:1/18:0/20:4/22:6)	749.5586
Negative	CL 76:11; [M-2H](2-); CL(16:1/20:4/22:6/18:0)	749.5479
Negative	CL 76:2; [M-2H](2-); CL(16:0/20:1/22:0/18:1)	757.616
Negative	CL 76:2; [M-2H](2-); CL(18:0/18:1/20:0/20:1)	756.6089
Negative	CL 76:2; [M-2H](2-); CL(20:1/16:0/20:1/20:0)	756.5941
Negative	CL 76:3; [M-2H](2-); CL(16:0/20:1/20:1/20:1)	756.596
Negative	CL 76:4; [M-2H](2-); CL(18:1/20:0/18:1/20:2)	756.554
Negative	CL 76:4; [M-2H](2-); CL(18:1/22:0/18:1/18:2)	756.5524
Negative	CL 76:8; [M-2H](2-); CL(18:0/20:3/18:1/20:4)	750.5471
Negative	CL 76:8; [M-2H](2-); CL(18:1/18:2/20:1/20:4)	752.558
Negative	CL 76:8; [M-2H](2-); CL(18:1/20:2/18:1/20:4)	752.5949
Negative	CL 76:9; [M-2H](2-); CL(16:0/18:0/20:4/22:5)	749.5361
Negative	CL 76:9; [M-2H](2-); CL(16:0/18:0/22:5/20:4)	749.5297
Negative	CL 76:9; [M-2H](2-); CL(16:0/20:4/18:0/22:5)	749.537
Negative	CL 76:9; [M-2H](2-); CL(18:0/16:0/20:4/22:5)	749.536
Negative	CL 76:9; [M-2H](2-); CL(18:2/20:4/20:2/18:1)	750.54
Negative	CL 76:9; [M-2H](2-); CL(20:4/16:0/22:5/18:0)	749.527
Negative	CL 78:10; [M-2H](2-); CL(18:2/22:5/20:3/18:0)	764.5455
Negative	CL 78:11; [M-2H](2-); CL(16:0/22:5/22:6/18:0)	763.5708
Negative	CL 78:12; [M-2H](2-); CL(22:6/16:0/22:6/18:0)	762.5082
Negative	CL 78:2; [M-2H](2-); CL(20:0/18:1/20:0/20:1)	772.5895

Negative	CL 78:4; [M-2H](2-); CL(20:0/18:2/20:0/20:2)	770.5967
Negative	CL 78:6; [M-2H](2-); CL(18:0/20:1/20:2/20:3)	768.5564
Negative	CL 78:6; [M-2H](2-); CL(18:1/20:0/20:2/20:3)	768.5941
Negative	CL 78:8; [M-2H](2-); CL(20:2/18:1/20:2/20:3)	766.5618
Negative	CL 80:10; [M-2H](2-); CL(20:3/20:0/20:3/20:4)	778.5455
Negative	CL 80:12; [M-2H](2-); CL(18:0/18:1/22:5/22:6)	774.5437
Negative	CL 80:12; [M-2H](2-); CL(18:2/22:5/20:2/20:3)	776.5556
Negative	CL 80:13; [M-2H](2-); CL(18:0/18:2/22:5/22:6)	774.542
Negative	CL 80:14; [M-2H](2-); CL(18:0/22:6/20:4/20:4)	772.5345
Negative	CL 80:14; [M-2H](2-); CL(18:1/22:5/20:4/20:4)	772.5576
Negative	CL 80:14; [M-2H](2-); CL(20:4/18:0/20:4/22:6)	772.5296
Negative	CL 80:14; [M-2H](2-); CL(20:4/18:1/20:4/22:5)	772.544
Negative	CL 80:9; [M-2H](2-); CL(18:0/20:0/20:4/22:5)	778.5515
Negative	CL 82:12; [M-2H](2-); CL(20:1/18:0/22:5/22:6)	790.5397
Negative	CL 82:14; [M-2H](2-); CL(18:1/20:1/22:6/22:6)	786.6361
Negative	CL 82:16; [M-2H](2-); CL(22:6/18:0/22:6/20:4)	786.5403
Negative	CL 84:1; [M-2H](2-); CL(22:0/22:0/22:0/18:1)	815.5773
Negative	CL 84:14; [M-2H](2-); CL(22:5/20:0/22:5/20:4)	802.5753
Negative	CL 84:15; [M-2H](2-); CL(20:4/20:1/22:5/22:5)	800.5759
Negative	CL 84:18; [M-2H](2-); CL(20:4/22:5/20:4/22:5)	798.5407
Negative	DGDG 20:1; [M-H]-; DGDG(2:0/18:1(11E))	720.3666
Negative	lysoPE 18:0; [M-H]-; PE(18:0/0:0)	480.3081
Negative	lysoPE 18:1; [M-H]-; PE(18:1(11E)/0:0)	477.1981
Negative	lysoPE 20:4; [M-H]-; PE(20:4(5E,8E,11E,14E)/0:0)	500.2785
Negative	lysoPE 22:4; [M-H]-; PE(22:4(7Z,10Z,13Z,16Z)/0:0)	528.3079
Negative	MGDG 28:2; [M-H]-; MGDG(2:0/26:2(5E,9Z))	670.5885
Negative	MGDG 34:1; [M-H]-; MGDG(16:0/18:1(11E))	756.5873
Negative	MGDG 36:1; [M-H]-; MGDG(16:0/20:1(11E))	784.6204
Negative	MGDG 36:2; [M-H]-; MGDG(18:0/18:2(2E,4E))	782.5331
Negative	MGDG 36:4; [M-H]-; MGDG(16:0/20:4(5E,8E,11E,14E))	778.5572
Negative	MGDG 38:3; [M-H]-; MGDG(18:1(11E)/20:2(11Z,14Z))	808.6243
Negative	MGDG 38:5; [M-H]-; MGDG(18:1(11E)/20:4(5E,8E,11E,14E))	802.5706
Negative	MGDG 38:6; [M-H]-; MGDG(18:2(2E,4E)/20:4(5E,8E,11E,14E))	800.5527
Negative	MGDG 40:3; [M-H]-; MGDG(20:1(11E)/20:2(11Z,14Z))	836.6549
Negative	N-(hexadecanoyl)-sphing-4-enine; [M-H]-; Cer(d18:1(4E)/16:0)	536.5187
Negative	PA 16:0; [M-H]-; GPA(4:0/12:0)	423.4188
Negative	PA 19:1; [M-H]-; GPA(2:0/17:1(9Z))	464.311
Negative	PA 19:2; [M-H]-; GPA(2:0/17:2(9Z,12Z))	462.2967
Negative	PA 26:3; [M-H]-; GPA(8:0/18:3(6Z,9Z,12Z))	558.4651
Negative	PA 35:2; [M-H]-; GPA(17:1(9Z)/18:1(11E))	686.5144
Negative	PA 38:2; [M-H]-; GPA(18:1(11E)/20:1(11E))	728.5619

Negative	PA 40:1; [M-H]-; GPA(20:0/20:1(11E))	756.6083
Negative	PA 40:5; [M-H]-; GPA(18:0/22:5(4Z,7Z,10Z,13Z,16Z))	748.5604
Negative	PA 40:6; [M-H]-; GPA(18:0/22:6(4Z,7Z,10Z,13Z,16Z,19Z))	746.5122
Negative	PA 42:2; [M-H]-; GPA(20:1(11E)/22:1(13Z))	782.6085
Negative	PA 42:8; [M-H]-; GPA(20:2(11Z,14Z)/22:6(4Z,7Z,10Z,13Z,16Z,19Z))	770.5104
Negative	PA 44:1; [M-H]-; GPA(18:1(11E)/26:0)	812.6171
Negative	PA 44:11; [M-H]-; GPA(22:5(4Z,7Z,10Z,13Z,16Z)/22:6(4Z,7Z,10Z,13Z,16Z,19Z))	792.55
Negative	PA 44:2; [M-H]-; GPA(20:1(11E)/24:1(15Z))	812.6594
Negative	PA 46:4; [M-H]-; GPA(20:2(11Z,14Z)/26:2(5E,9Z))	836.6498
Negative	PC 32:1; [M+HCOO]-; GPCho(15:1(9Z)/17:0)	776.5263
Negative	PC 33:2; [M-Ac-H]-; GPCho(15:0/18:2(2E,4E))	802.5568
Negative	PC 33:2; [M-Ac-H]-; GPCho(16:1(7Z)/17:1(9Z))	802.5624
Negative	PC 34:0; [M-Ac-H]-; GPCho(17:0/17:0)	820.6432
Negative	PC 34:3; [M+HCOO]-; GPCho(16:1(7Z)/18:2(2E,4E))	800.5637
Negative	PC 34:3; [M-Ac-H]-; GPCho(16:0/18:3(6Z,9Z,12Z))	814.5576
Negative	PC 34:3; [M-Ac-H]-; GPCho(16:1(7Z)/18:2(2E,4E))	814.561
Negative	PC 34:5; [M-CH3]-; GPCho(14:0/20:5(5Z,8Z,11Z,14Z,17Z))	736.4947
Negative	PC 35:3; [M+HCOO]-; GPCho(17:2(9Z,12Z)/18:1(11E))	814.558
Negative	PC 35:3; [M-Ac-H]-; GPCho(17:1(9Z)/18:2(2E,4E))	828.5813
Negative	PC 35:3; [M-Ac-H]-; GPCho(17:2(9Z,12Z)/18:1(11E))	828.6092
Negative	PC 36:2; [M+HCOO]-; GPCho(16:1(7Z)/20:1(11E))	830.6216
Negative	PC 36:2; [M+HCOO]-; GPCho(18:0/18:2(2E,4E))	831.6127
Negative	PC 36:4; [M+HCOO]-; GPCho(18:2(2E,4E)/18:2(2E,4E))	826.5652
Negative	PC 38:3; [M-CH3]-; GPCho(20:2(11Z,14Z)/18:1(11E))	796.5883
Negative	PC 38:6; [M+HCOO]-; GPCho(16:0/22:6(4Z,7Z,10Z,13Z,16Z,19Z))	850.566
Negative	PC 39:1; [M-Ac-H]-; GPCho(18:1(11E)/21:0)	888.7073
Negative	PC 39:4; [M-Ac-H]-; GPCho(19:0/20:4(5E,8E,11E,14E))	882.663
Negative	PC 39:5; [M-Ac-H]-; GPCho(17:1(9Z)/22:4(7Z,10Z,13Z,16Z))	880.6434
Negative	PC 40:1; [M-Ac-H]-; GPCho(18:1(11E)/22:0)	902.6997
Negative	PC 40:4; [M-Ac-H]-; GPCho(20:1(11E)/20:3(5Z,8Z,11Z))	896.6333
Negative	PC 40:5; [M-Ac-H]-; GPCho(18:1(11E)/22:4(7Z,10Z,13Z,16Z))	894.6292
Negative	PC 40:5; [M-Ac-H]-; GPCho(20:2(11Z,14Z)/20:3(5Z,8Z,11Z))	894.6236
Negative	PC 40:5; [M-CH3]-; GPCho(18:1(11E)/22:4(7Z,10Z,13Z,16Z))	821.5333
Negative	PC 40:5; [M-CH3]-; GPCho(22:4(7Z,10Z,13Z,16Z)/18:1(11E))	821.5305
Negative	PC 40:7; [M-Ac-H]-; GPCho(20:3(5Z,8Z,11Z)/20:4(5E,8E,11E,14E))	890.6028
Negative	PC 42:8; [M-CH3]-; GPCho(22:5(4Z,7Z,10Z,13Z,16Z)/20:3(5Z,8Z,11Z))	843.523
Negative	PE 30:0; [M-H]-; GPEtn(14:0/16:0)	662.4769
Negative	PE 31:1; [M-H]-; GPEtn(15:0/16:1(7Z))	674.5144
Negative	PE 32:0; [M-H]-; GPEtn(16:0/16:0)	690.5023
Negative	PE 32:1; [M-H]-; GPEtn(14:0/18:1(11E))	688.4937
Negative	PE 32:1; [M-H]-; GPEtn(16:0/16:1(7Z))	688.4931

Negative	PE 32:2; [M-H]-; GPEtn(14:0/18:2(2E,4E))	686.4773
Negative	PE 32:2; [M-H]-; GPEtn(14:1(9Z)/18:1(11E))	686.4766
Negative	PE 32:2; [M-H]-; GPEtn(16:1(7Z)/16:1(7Z))	686.4798
Negative	PE 33:1; [M-H]-; GPEtn(15:0/18:1(11E))	702.5098
Negative	PE 33:1; [M-H]-; GPEtn(16:1(7Z)/17:0)	702.5071
Negative	PE 33:2; [M-H]-; GPEtn(15:0/18:2(2E,4E))	700.4932
Negative	PE 33:2; [M-H]-; GPEtn(16:1(7Z)/17:1(9Z))	700.493
Negative	PE 34:0; [M-H]-; GPEtn(16:0/18:0)	718.5404
Negative	PE 34:0; [M-H]-; GPEtn(17:0/17:0)	718.5348
Negative	PE 34:1; [M-H]-; GPEtn(16:0/18:1(11E))	716.5232
Negative	PE 34:2; [M-H]-; GPEtn(16:0/18:2(2E,4E))	714.5066
Negative	PE 34:2; [M-H]-; GPEtn(16:1(7Z)/18:1(11E))	714.5106
Negative	PE 34:3; [M-H]-; GPEtn(16:0/18:3(6Z,9Z,12Z))	712.4932
Negative	PE 35:1; [M-H]-; GPEtn(17:0/18:1(11E))	730.5381
Negative	PE 35:2; [M-H]-; GPEtn(17:0/18:2(2E,4E))	728.5237
Negative	PE 35:2; [M-H]-; GPEtn(17:1(9Z)/18:1(11E))	728.5887
Negative	PE 35:3; [M-H]-; GPEtn(17:1(9Z)/18:2(2E,4E))	726.5082
Negative	PE 35:4; [M-H]-; GPEtn(15:0/20:4(5E,8E,11E,14E))	724.4924
Negative	PE 36:0; [M-H]-; GPEtn(16:0/20:0)	746.5729
Negative	PE 36:1; [M-H]-; GPEtn(18:0/18:1(11E))	744.5582
Negative	PE 36:2; [M-H]-; GPEtn(18:0/18:2(2E,4E))	742.5402
Negative	PE 36:2; [M-H]-; GPEtn(18:1(11E)/18:1(11E))	742.541
Negative	PE 36:3; [M-H]-; GPEtn(16:0/20:3(5Z,8Z,11Z))	740.6008
Negative	PE 36:3; [M-H]-; GPEtn(18:1(11E)/18:2(2E,4E))	740.5262
Negative	PE 36:4; [M-H]-; GPEtn(18:1(11E)/18:3(6Z,9Z,12Z))	738.507
Negative	PE 36:4; [M-H]-; GPEtn(18:2(2E,4E)/18:2(2E,4E))	738.5104
Negative	PE 36:5; [M-H]-; GPEtn(16:0/20:5(5Z,8Z,11Z,14Z,17Z))	736.493
Negative	PE 36:5; [M-H]-; GPEtn(16:1(7Z)/20:4(5E,8E,11E,14E))	736.4913
Negative	PE 36:5; [M-H]-; GPEtn(18:2(2E,4E)/18:3(6Z,9Z,12Z))	736.4952
Negative	PE 37:2; [M-H]-; GPEtn(18:2(2E,4E)/19:0)	756.5509
Negative	PE 37:4; [M-H]-; GPEtn(17:0/20:4(5E,8E,11E,14E))	752.5258
Negative	PE 37:5; [M-H]-; GPEtn(17:1(9Z)/20:4(5E,8E,11E,14E))	750.5123
Negative	PE 38:1; [M-H]-; GPEtn(18:0/20:1(11E))	772.5843
Negative	PE 38:1; [M-H]-; GPEtn(18:1(11E)/20:0)	772.5862
Negative	PE 38:2; [M-H]-; GPEtn(18:0/20:2(11Z,14Z))	770.5722
Negative	PE 38:2; [M-H]-; GPEtn(18:2(2E,4E)/20:0)	770.5702
Negative	PE 38:3; [M-H]-; GPEtn(18:1(11E)/20:2(11Z,14Z))	768.5549
Negative	PE 38:3; [M-H]-; GPEtn(18:2(2E,4E)/20:1(11E))	768.5561
Negative	PE 38:4; [M-H]-; GPEtn(16:0/22:4(7Z,10Z,13Z,16Z))	766.5424
Negative	PE 38:4; [M-H]-; GPEtn(18:0/20:4(5E,8E,11E,14E))	766.5415
Negative	PE 38:4; [M-H]-; GPEtn(18:1(11E)/20:3(5Z,8Z,11Z))	766.5395

Negative	PE 38:4; [M-H]-; GPEtn(18:2(2E,4E)/20:2(11Z,14Z))	766.5376
Negative	PE 38:5; [M-H]-; GPEtn(18:0/20:5(5Z,8Z,11Z,14Z,17Z))	764.5246
Negative	PE 38:5; [M-H]-; GPEtn(18:1(11E)/20:4(5E,8E,11E,14E))	764.5243
Negative	PE 38:5; [M-H]-; GPEtn(18:2(2E,4E)/20:3(5Z,8Z,11Z))	765.5565
Negative	PE 38:6; [M-H]-; GPEtn(16:0/22:6(4Z,7Z,10Z,13Z,16Z,19Z))	762.577
Negative	PE 39:1; [M-H]-; GPEtn(18:1(11E)/21:0)	786.5998
Negative	PE 39:4; [M-H]-; GPEtn(17:0/22:4(7Z,10Z,13Z,16Z))	780.5519
Negative	PE 39:5; [M-H]-; GPEtn(17:0/22:5(4Z,7Z,10Z,13Z,16Z))	778.5447
Negative	PE 40:1; [M-H]-; GPEtn(16:0/24:1(15Z))	800.6147
Negative	PE 40:1; [M-H]-; GPEtn(18:0/22:1(13Z))	800.623
Negative	PE 40:1; [M-H]-; GPEtn(18:1(11E)/22:0)	800.6201
Negative	PE 40:1; [M-H]-; GPEtn(20:0/20:1(11E))	800.6152
Negative	PE 40:2; [M-H]-; GPEtn(18:0/22:2(13Z,16Z))	798.529
Negative	PE 40:2; [M-H]-; GPEtn(18:1(11E)/22:1(13Z))	798.6009
Negative	PE 40:2; [M-H]-; GPEtn(18:2(2E,4E)/22:0)	798.6038
Negative	PE 40:3; [M-H]-; GPEtn(18:1(11E)/22:2(13Z,16Z))	796.5865
Negative	PE 40:3; [M-H]-; GPEtn(18:2(2E,4E)/22:1(13Z))	796.5836
Negative	PE 40:3; [M-H]-; GPEtn(20:0/20:3(5Z,8Z,11Z))	796.586
Negative	PE 40:4; [M-H]-; GPEtn(16:0/24:4(5Z,8Z,11Z,14Z))	794.491
Negative	PE 40:4; [M-H]-; GPEtn(18:0/22:4(7Z,10Z,13Z,16Z))	794.571
Negative	PE 40:4; [M-H]-; GPEtn(20:0/20:4(5E,8E,11E,14E))	794.5692
Negative	PE 40:4; [M-H]-; GPEtn(20:1(11E)/20:3(5Z,8Z,11Z))	794.5998
Negative	PE 40:5; [M-H]-; GPEtn(18:0/22:5(4Z,7Z,10Z,13Z,16Z))	792.5545
Negative	PE 40:5; [M-H]-; GPEtn(18:1(11E)/22:4(7Z,10Z,13Z,16Z))	792.5563
Negative	PE 40:5; [M-H]-; GPEtn(20:1(11E)/20:4(5E,8E,11E,14E))	792.5534
Negative	PE 40:6; [M-H]-; GPEtn(18:1(11E)/22:5(4Z,7Z,10Z,13Z,16Z))	790.539
Negative	PE 40:6; [M-H]-; GPEtn(20:2(11Z,14Z)/20:4(5E,8E,11E,14E))	790.5432
Negative	PE 40:7; [M-H]-; GPEtn(18:2(2E,4E)/22:5(4Z,7Z,10Z,13Z,16Z))	788.5211
Negative	PE 40:8; [M-H]-; GPEtn(18:2(2E,4E)/22:6(4Z,7Z,10Z,13Z,16Z,19Z))	786.5356
Negative	PE 41:2; [M-H]-; GPEtn(18:2(2E,4E)/23:0)	812.618
Negative	PE 42:0; [M-H]-; GPEtn(18:0/24:0)	830.6082
Negative	PE 42:1; [M-H]-; GPEtn(16:0/26:1(5Z))	828.6468
Negative	PE 42:1; [M-H]-; GPEtn(18:1(11E)/24:0)	828.6482
Negative	PE 42:2; [M-H]-; GPEtn(18:1(11E)/24:1(15Z))	826.6372
Negative	PE 42:2; [M-H]-; GPEtn(18:2(2E,4E)/24:0)	826.6352
Negative	PE 42:3; [M-H]-; GPEtn(18:2(2E,4E)/24:1(15Z))	824.6166
Negative	PE 42:3; [M-H]-; GPEtn(18:3(6Z,9Z,12Z)/24:0)	824.6185
Negative	PE 42:3; [M-H]-; GPEtn(20:3(5Z,8Z,11Z)/22:0)	824.6214
Negative	PE 42:4; [M-H]-; GPEtn(18:0/24:4(5Z,8Z,11Z,14Z))	822.6024
Negative	PE 42:4; [M-H]-; GPEtn(20:3(5Z,8Z,11Z)/22:1(13Z))	822.6176
Negative	PE 42:4; [M-H]-; GPEtn(20:4(5E,8E,11E,14E)/22:0)	822.6052

Negative	PE 42:5; [M-H]-; GPEtn(20:0/22:5(4Z,7Z,10Z,13Z,16Z))	820.6367
Negative	PE 42:5; [M-H]-; GPEtn(20:1(11E)/22:4(7Z,10Z,13Z,16Z))	820.5914
Negative	PE 42:8; [M-H]-; GPEtn(20:2(11Z,14Z)/22:6(4Z,7Z,10Z,13Z,16Z,19Z))	814.5436
Negative	PE 44:1; [M-H]-; GPEtn(20:1(11E)/24:0)	856.6864
Negative	PE 44:12; [M-H]-; GPEtn(22:6(4Z,7Z,10Z,13Z,16Z,19Z)/22:6(4Z,7Z,10Z,13Z,16Z,19Z))	834.5256
Negative	PE 44:4; [M-H]-; GPEtn(20:4(5E,8E,11E,14E)/24:0)	850.6369
Negative	PG 32:0; [M-H]-; GPGro(16:0/16:0)	721.5022
Negative	PG 33:0; [M-H]-; GPGro(16:0/17:0)	735.6261
Negative	PG 34:0; [M-H]-; GPGro(16:0/18:0)	749.5346
Negative	PG 34:1; [M-H]-; GPGro(16:0/18:1(11E))	747.5204
Negative	PG 34:2; [M-H]-; GPGro(16:0/18:2(2E,4E))	745.5023
Negative	PG 34:2; [M-H]-; GPGro(16:1(7Z)/18:1(11E))	745.4993
Negative	PG 35:1; [M-H]-; GPGro(17:0/18:1(11E))	761.5366
Negative	PG 36:0; [M-H]-; GPGro(18:0/18:0)	777.5701
Negative	PG 36:1; [M-H]-; GPGro(18:0/18:1(11E))	775.5924
Negative	PG 36:2; [M-H]-; GPGro(16:0/20:2(11Z,14Z))	773.5346
Negative	PG 36:2; [M-H]-; GPGro(18:1(11E)/18:1(11E))	773.537
Negative	PG 36:3; [M-H]-; GPGro(16:0/20:3(5Z,8Z,11Z))	771.5233
Negative	PG 36:4; [M-H]-; GPGro(16:0/20:4(5E,8E,11E,14E))	769.5042
Negative	PG 36:4; [M-H]-; GPGro(18:2(2E,4E)/18:2(2E,4E))	769.5075
Negative	PG 38:2; [M-H]-; GPGro(18:0/20:2(11Z,14Z))	801.6173
Negative	PG 38:3; [M-H]-; GPGro(18:0/20:3(5Z,8Z,11Z))	799.5477
Negative	PG 38:3; [M-H]-; GPGro(18:1(11E)/20:2(11Z,14Z))	799.552
Negative	PG 38:4; [M-H]-; GPGro(18:0/20:4(5E,8E,11E,14E))	797.5395
Negative	PG 38:5; [M-H]-; GPGro(18:1(11E)/20:4(5E,8E,11E,14E))	795.5178
Negative	PG 38:5; [M-H]-; GPGro(18:2(2E,4E)/20:3(5Z,8Z,11Z))	795.5195
Negative	PG 38:6; [M-H]-; GPGro(16:0/22:6(4Z,7Z,10Z,13Z,16Z,19Z))	793.5035
Negative	PG 38:6; [M-H]-; GPGro(18:2(2E,4E)/20:4(5E,8E,11E,14E))	793.5039
Negative	PG 40:3; [M-H]-; GPGro(18:1(11E)/22:2(13Z,16Z))	827.5861
Negative	PG 40:4; [M-H]-; GPGro(18:0/22:4(7Z,10Z,13Z,16Z))	825.5653
Negative	PG 40:4; [M-H]-; GPGro(20:1(11E)/20:3(5Z,8Z,11Z))	825.5628
Negative	PG 40:5; [M-H]-; GPGro(18:1(11E)/22:4(7Z,10Z,13Z,16Z))	823.5525
Negative	PG 40:6; [M-H]-; GPGro(18:0/22:6(4Z,7Z,10Z,13Z,16Z,19Z))	821.5378
Negative	PG 40:6; [M-H]-; GPGro(18:1(11E)/22:5(4Z,7Z,10Z,13Z,16Z))	821.537
Negative	PG 40:6; [M-H]-; GPGro(18:2(2E,4E)/22:4(7Z,10Z,13Z,16Z))	821.5645
Negative	PG 40:7; [M-H]-; GPGro(18:1(11E)/22:6(4Z,7Z,10Z,13Z,16Z,19Z))	819.66
Negative	PG 40:7; [M-H]-; GPGro(20:3(5Z,8Z,11Z)/20:4(5E,8E,11E,14E))	819.5189
Negative	PG 42:6; [M-H]-; GPGro(20:1(11E)/22:5(4Z,7Z,10Z,13Z,16Z))	849.566
Negative	PG 42:6; [M-H]-; GPGro(20:2(11Z,14Z)/22:4(7Z,10Z,13Z,16Z))	849.5703
Negative	PG 42:9; [M-H]-; GPGro(20:3(5Z,8Z,11Z)/22:6(4Z,7Z,10Z,13Z,16Z,19Z))	843.5187
Negative	PG 42:9; [M-H]-; GPGro(20:4(5E,8E,11E,14E)/22:5(4Z,7Z,10Z,13Z,16Z))	843.5203

Negative	PI 32:0; [M-H]-; GPIns(16:0/16:0)	809.5235
Negative	PI 34:0; [M-H]-; GPIns(16:0/18:0)	837.5539
Negative	PI 34:1; [M-H]-; GPIns(16:0/18:1(11E))	835.532
Negative	PI 34:1; [M-H]-; GPIns(16:1(7Z)/18:0)	835.5649
Negative	PI 34:2; [M-H]-; GPIns(16:1(7Z)/18:1(11E))	833.5264
Negative	PI 35:1; [M-H]-; GPIns(17:0/18:1(11E))	849.5531
Negative	PI 36:1; [M-H]-; GPIns(18:0/18:1(11E))	863.5634
Negative	PI 36:2; [M-H]-; GPIns(18:1(11E)/18:1(11E))	861.5547
Negative	PI 38:2; [M-H]-; GPIns(18:0/20:2(11Z,14Z))	889.5788
Negative	PI 38:3; [M-H]-; GPIns(18:0/20:3(5Z,8Z,11Z))	887.5686
Negative	PI 38:4; [M-H]-; GPIns(16:0/22:4(7Z,10Z,13Z,16Z))	885.5553
Negative	PI 38:4; [M-H]-; GPIns(18:0/20:4(5E,8E,11E,14E))	885.556
Negative	PI 38:4; [M-H]-; GPIns(18:1(11E)/20:3(5Z,8Z,11Z))	885.5729
Negative	PI 38:6; [M-H]-; GPIns(16:0/22:6(4Z,7Z,10Z,13Z,16Z,19Z))	881.5209
Negative	PI 38:6; [M-H]-; GPIns(18:2(2E,4E)/20:4(5E,8E,11E,14E))	881.5192
Negative	PI 40:4; [M-H]-; GPIns(18:0/22:4(7Z,10Z,13Z,16Z))	913.5858
Negative	PI 40:4; [M-H]-; GPIns(20:0/20:4(5E,8E,11E,14E))	913.5823
Negative	PI 40:5; [M-H]-; GPIns(18:0/22:5(4Z,7Z,10Z,13Z,16Z))	911.5678
Negative	plasmenyl-PE 32:1; [M-H]-; PE(P-16:0/16:1(7Z))	672.4991
Negative	plasmenyl-PE 33:0; [M-H]-; PE(P-16:0/17:0)	688.2674
Negative	plasmenyl-PE 33:1; [M-H]-; PE(P-16:0/17:1(9Z))	686.5149
Negative	plasmenyl-PE 33:1; [M-H]-; PE(P-18:0/15:1(9Z))	686.2684
Negative	plasmenyl-PE 34:1; [M-H]-; PE(P-16:0/18:1(11E))	700.5325
Negative	plasmenyl-PE 34:1; [M-H]-; PE(P-18:0/16:1(7Z))	700.4941
Negative	plasmenyl-PE 34:2; [M-H]-; PE(P-16:0/18:2(2E,4E))	698.5154
Negative	plasmenyl-PE 35:0; [M-H]-; PE(P-18:0/17:0)	716.5584
Negative	plasmenyl-PE 36:0; [M-H]-; PE(P-18:0/18:0)	730.6046
Negative	plasmenyl-PE 36:0; [M-H]-; PE(P-20:0/16:0)	730.5758
Negative	plasmenyl-PE 36:1; [M-H]-; PE(P-18:0/18:1(11E))	728.52
Negative	plasmenyl-PE 36:3; [M-H]-; PE(P-16:0/20:3(5Z,8Z,11Z))	724.5259
Negative	plasmenyl-PE 36:3; [M-H]-; PE(P-18:0/18:3(6Z,9Z,12Z))	724.5261
Negative	plasmenyl-PE 36:3; [M-H]-; PE(P-18:0/18:3(9Z,12Z,15Z))	724.5283
Negative	plasmenyl-PE 36:4; [M-H]-; PE(P-16:0/20:4(5E,8E,11E,14E))	722.5147
Negative	plasmenyl-PE 36:5; [M-H]-; PE(P-16:0/20:5(5Z,8Z,11Z,14Z,17Z))	720.5009
Negative	plasmenyl-PE 38:1; [M-H]-; PE(P-16:0/22:1(13Z))	756.6168
Negative	plasmenyl-PE 38:1; [M-H]-; PE(P-18:0/20:1(11E))	756.5904
Negative	plasmenyl-PE 38:1; [M-H]-; PE(P-20:0/18:1(11E))	756.6242
Negative	plasmenyl-PE 38:4; [M-H]-; PE(P-16:0/22:4(7Z,10Z,13Z,16Z))	750.5467
Negative	plasmenyl-PE 38:4; [M-H]-; PE(P-18:0/20:4(5E,8E,11E,14E))	750.5463
Negative	plasmenyl-PE 38:5; [M-H]-; PE(P-16:0/22:5(4Z,7Z,10Z,13Z,16Z))	748.5282
Negative	plasmenyl-PE 38:5; [M-H]-; PE(P-18:0/20:5(5Z,8Z,11Z,14Z,17Z))	748.591

Negative	plasmenyl-PE 38:6; [M-H]-; PE(P-16:0/22:6(4Z,7Z,10Z,13Z,16Z,19Z))	746.5143
Negative	plasmenyl-PE 40:1; [M-H]-; PE(P-18:0/22:1(13Z))	784.6219
Negative	plasmenyl-PE 40:2; [M-H]-; PE(P-18:0/22:2(13Z,16Z))	782.609
Negative	plasmenyl-PE 40:4; [M-H]-; PE(P-16:0/24:4(5Z,8Z,11Z,14Z))	778.5744
Negative	plasmenyl-PE 40:4; [M-H]-; PE(P-20:0/20:4(5E,8E,11E,14E))	778.5765
Negative	plasmenyl-PE 40:5; [M-H]-; PE(P-18:0/22:5(4Z,7Z,10Z,13Z,16Z))	776.5598
Negative	plasmenyl-PE 42:1; [M-H]-; PE(P-18:0/24:1(15Z))	812.6592
Negative	plasmenyl-PE 42:4; [M-H]-; PE(P-18:0/24:4(5Z,8Z,11Z,14Z))	806.6059
Negative	plasmenyl-PE 42:4; [M-H]-; PE(P-20:0/22:4(7Z,10Z,13Z,16Z))	806.6109
Negative	plasmenyl-PE 42:5; [M-H]-; PE(P-20:0/22:5(4Z,7Z,10Z,13Z,16Z))	804.5943
Negative	plasmenyl-PE 42:6; [M-H]-; PE(P-20:0/22:6(4Z,7Z,10Z,13Z,16Z,19Z))	802.5783
Negative	PS 31:0; [M-H]-; GPSer(15:0/16:0)	720.5092
Negative	PS 32:0; [M-H]-; GPSer(14:0/18:0)	734.5349
Negative	PS 33:1; [M-H]-; GPSer(15:0/18:1(11E))	746.5406
Negative	PS 33:2; [M-H]-; GPSer(15:1(9Z)/18:1(11E))	744.6048
Negative	PS 34:0; [M-H]-; GPSer(16:0/18:0)	762.5222
Negative	PS 34:1; [M-H]-; GPSer(16:0/18:1(11E))	760.5137
Negative	PS 34:1; [M-H]-; GPSer(16:1(7Z)/18:0)	760.515
Negative	PS 34:2; [M-H]-; GPSer(16:1(7Z)/18:1(11E))	758.5333
Negative	PS 35:2; [M-H]-; GPSer(17:0/18:2(2E,4E))	772.5421
Negative	PS 35:2; [M-H]-; GPSer(17:1(9Z)/18:1(11E))	772.5904
Negative	PS 36:1; [M-H]-; GPSer(18:0/18:1(11E))	788.6239
Negative	PS 36:2; [M-H]-; GPSer(18:0/18:2(2E,4E))	786.5311
Negative	PS 36:2; [M-H]-; GPSer(18:1(11E)/18:1(11E))	786.5305
Negative	PS 36:3; [M-H]-; GPSer(16:0/20:3(5Z,8Z,11Z))	784.5141
Negative	PS 36:3; [M-H]-; GPSer(18:0/18:3(6Z,9Z,12Z))	784.5128
Negative	PS 36:3; [M-H]-; GPSer(18:1(11E)/18:2(2E,4E))	784.5157
Negative	PS 37:2; [M-H]-; GPSer(18:2(2E,4E)/19:0)	800.5883
Negative	PS 37:3; [M-H]-; GPSer(17:0/20:3(5Z,8Z,11Z))	798.5325
Negative	PS 37:5; [M-H]-; GPSer(17:1(9Z)/20:4(5E,8E,11E,14E))	794.5344
Negative	PS 38:1; [M-H]-; GPSer(18:0/20:1(11E))	816.5719
Negative	PS 38:1; [M-H]-; GPSer(18:1(11E)/20:0)	816.5787
Negative	PS 38:2; [M-H]-; GPSer(18:0/20:2(11Z,14Z))	814.5618
Negative	PS 38:2; [M-H]-; GPSer(18:1(11E)/20:1(11E))	814.5611
Negative	PS 38:2; [M-H]-; GPSer(18:2(2E,4E)/20:0)	814.5557
Negative	PS 38:3; [M-H]-; GPSer(18:1(11E)/20:2(11Z,14Z))	812.6028
Negative	PS 38:3; [M-H]-; GPSer(18:2(2E,4E)/20:1(11E))	812.5414
Negative	PS 38:4; [M-H]-; GPSer(16:0/22:4(7Z,10Z,13Z,16Z))	810.5296
Negative	PS 38:4; [M-H]-; GPSer(18:0/20:4(5E,8E,11E,14E))	810.5334
Negative	PS 38:4; [M-H]-; GPSer(18:1(11E)/20:3(5Z,8Z,11Z))	810.5314
Negative	PS 38:5; [M-H]-; GPSer(18:1(11E)/20:4(5E,8E,11E,14E))	808.5144

Negative	PS 39:4; [M-H]-; GPSer(19:0/20:4(5E,8E,11E,14E))	824.5783
Negative	PS 39:5; [M-H]-; GPSer(17:0/22:5(4Z,7Z,10Z,13Z,16Z))	822.5394
Negative	PS 40:1; [M-H]-; GPSer(16:1(7Z)/24:0)	844.6104
Negative	PS 40:1; [M-H]-; GPSer(18:1(11E)/22:0)	844.6089
Negative	PS 40:2; [M-H]-; GPSer(18:2(2E,4E)/22:0)	842.59
Negative	PS 40:3; [M-H]-; GPSer(18:1(11E)/22:2(13Z,16Z))	840.5802
Negative	PS 40:3; [M-H]-; GPSer(18:2(2E,4E)/22:1(13Z))	840.5755
Negative	PS 40:3; [M-H]-; GPSer(20:0/20:3(5Z,8Z,11Z))	840.5875
Negative	PS 40:4; [M-H]-; GPSer(18:0/22:4(7Z,10Z,13Z,16Z))	838.5618
Negative	PS 40:5; [M-H]-; GPSer(18:1(11E)/22:4(7Z,10Z,13Z,16Z))	836.5637
Negative	PS 40:6; [M-H]-; GPSer(18:0/22:6(4Z,7Z,10Z,13Z,16Z,19Z))	834.5313
Negative	PS 40:6; [M-H]-; GPSer(18:1(11E)/22:5(4Z,7Z,10Z,13Z,16Z))	834.5169
Negative	PS 40:7; [M-H]-; GPSer(18:1(11E)/22:6(4Z,7Z,10Z,13Z,16Z,19Z))	832.5145
Negative	PS 41:1; [M-H]-; GPSer(18:1(11E)/23:0)	858.6252
Negative	PS 42:2; [M-H]-; GPSer(18:1(11E)/24:1(15Z))	870.6239
Negative	PS 42:2; [M-H]-; GPSer(18:2(2E,4E)/24:0)	870.6279
Negative	PS 42:3; [M-H]-; GPSer(18:2(2E,4E)/24:1(15Z))	868.6084
Negative	PS 42:3; [M-H]-; GPSer(20:3(5Z,8Z,11Z)/22:0)	868.6097
Negative	PS 42:4; [M-H]-; GPSer(18:0/24:4(5Z,8Z,11Z,14Z))	866.5886
Negative	PS 42:4; [M-H]-; GPSer(20:3(5Z,8Z,11Z)/22:1(13Z))	866.5889
Negative	PS 42:4; [M-H]-; GPSer(20:4(5E,8E,11E,14E)/22:0)	866.595
Negative	PS 42:5; [M-H]-; GPSer(18:1(11E)/24:4(5Z,8Z,11Z,14Z))	864.6749
Negative	PS 42:5; [M-H]-; GPSer(20:0/22:5(4Z,7Z,10Z,13Z,16Z))	864.5739
Negative	PS 42:5; [M-H]-; GPSer(20:4(5E,8E,11E,14E)/22:1(13Z))	864.5892
Negative	PS 44:12; [M-H]-; GPSer(22:6(4Z,7Z,10Z,13Z,16Z,19Z)/22:6(4Z,7Z,10Z,13Z,16Z,19Z))	878.5072
Negative	PS 44:3; [M-H]-; GPSer(20:3(5Z,8Z,11Z)/24:0)	896.6399
Negative	PS 44:4; [M-H]-; GPSer(20:4(5E,8E,11E,14E)/24:0)	894.6263
Negative	PS 44:5; [M-H]-; GPSer(20:4(5E,8E,11E,14E)/24:1(15Z))	892.6155
Negative	PS 44:6; [M-H]-; GPSer(22:0/22:6(4Z,7Z,10Z,13Z,16Z,19Z))	890.5987
Negative	PS 46:5; [M-H]-; GPSer(22:5(4Z,7Z,10Z,13Z,16Z)/24:0)	920.6399

**Supplementary Table S2.**	
**Race/ethnicity**	African American (Bladder Cancer n=12)
	European American (Bladder Cancer n=14)
**Stage**	pTa/pTis (n=1)
	pT1 (n=1)
	pT2 (n=1)
	pT3 (n=7)
	pT4 (n=4)
	Unknown (n=12)
**Gender**	Male (n= 15); Female (n=11)
**Smoking Status**	Current (n= 13); Former(n=5); Never (n=6); Unknown(n=2)

Supplementary Table S3:		
**Lipids**	**P-value_ttest**	**BH.P-value_ttest**
**CE(22:5); [M+NH4]+; 22:5 Cholesteryl ester**	0.001870612	0.117427191
**Unknown 68:2; [M-2H](2-); Unknown(16:0/18:1/16:1/18:0)**	0.005756968	0.163990521
**Unknown 70:3; [M-2H](2-); Unknown(16:0/18:0/18:1/18:2)**	0.005217288	0.155121165
**Unknown 70:4; [M-2H](2-); Unknown(16:1/18:1/18:2/18:0)**	0.001904225	0.117427191
**Unknown 72:5; [M-2H](2-); Unknown(18:1/18:0/18:2/18:2)**	0.008633848	0.189149436
**Unknown 84:1; [M-2H](2-); Unknown(22:0/22:0/22:0/18:1)**	0.007790179	0.18014788
**DG 32:1; [M+NH4]+; DG(14:0/18:1/0:0)**	0.003677427	0.150283111
**DG 32:1; [M+NH4]+; DG(16:0/16:1/0:0)**	0.003490523	0.150283111
**DG 34:2; [M+NH4]+; DG(16:0/18:2/0:0)**	0.00151822	0.113942359
**DG 34:2; [M+NH4]+; DG(16:1/18:1/0:0)**	0.002281658	0.135677184
**DGDG 20:1; [M-H]-; DGDG(2:0/18:1(11E))**	0.005911565	0.163990521
**lysoPC 20:3; [M+H]+; PC(20:3(5Z,8Z,11Z)/0:0)**	5.28951E-05	0.018733831
**lysoPC 22:1; [M+H]+; PC(22:1(13Z)/0:0)**	0.000173144	0.018733831
**lysoPC 22:2; [M+H]+; PC(22:2(13Z,16Z)/0:0)**	3.84731E-05	0.018733831
**lysoPC 26:1; [M+H]+; PC(26:1(5Z)/0:0)**	5.75057E-05	0.018733831
**N-(hexacosanoyl)-sphinganine; [M+H]+; Cer(d18:0/26:0)**	0.001573979	0.113942359
**PC 22:5; [M+HCOO]-; GPCho(2:0/20:5(5Z,8Z,11Z,14Z,17Z))**	0.000116392	0.018733831
**PC 28:0; [M-Ac-H]-; GPCho(14:0/14:0)**	0.006008061	0.163990521
**PC 31:6; [M+H]+; GPCho(9:0/22:6(4Z,7Z,10Z,13Z,16Z,19Z))**	9.61316E-05	0.018733831
**PC 32:0; [M+Na]+; GPCho(10:0/22:0)**	0.004294834	0.150283111
**PC 32:0; [M+Na]+; GPCho(11:0/21:0)**	0.004220369	0.150283111
**PC 32:0; [M+Na]+; GPCho(14:0/18:0)**	0.004198211	0.150283111
**PC 32:0; [M+Na]+; GPCho(15:0/17:0)**	0.004153284	0.150283111
**PC 32:0; [M+Na]+; GPCho(16:0/16:0)**	0.004124618	0.150283111
**PC 32:0; [M+Na]+; GPCho(19:0/13:0)**	0.004453794	0.150283111
**PC 32:0; [M+Na]+; GPCho(24:0/8:0)**	0.004141113	0.150283111
**PC 32:0; [M+Na]+; GPCho(6:0/26:0)**	0.004061015	0.150283111
**PC 32:0; [M+Na]+; GPCho(7:0/25:0)**	0.00411562	0.150283111
**PC 32:0; [M+Na]+; GPCho(8:0/24:0)**	0.003902275	0.150283111
**PC 32:1; [M+Na]+; GPCho(12:0/20:1(11Z))**	0.004153382	0.150283111
**PC 32:1; [M+Na]+; GPCho(16:0/16:1(7Z))**	0.003820828	0.150283111
**PC 32:2; [M+Na]+; GPCho(14:0/18:2(2E,4E))**	0.000180025	0.018733831
**PC 32:2; [M+Na]+; GPCho(16:1(7Z)/16:1(7Z))**	0.000139723	0.018733831
**PC 34:0; [M+H]+; GPCho(17:0/17:0)**	0.012345484	0.244705121
**PC 34:3; [M+H]+; GPCho(16:0/18:3(6Z,9Z,12Z))**	0.004214381	0.150283111
**PC 34:3; [M+H]+; GPCho(16:1(7Z)/18:2(2E,4E))**	0.004743267	0.150283111
**PC 34:3; [M+Na]+; GPCho(16:1(7Z)/18:2(2E,4E))**	0.004997502	0.151288013
**PC 34:3; [M-Ac-H]-; GPCho(16:1(7Z)/18:2(2E,4E))**	0.006178056	0.165095771
**PC 34:4; [M+H]+; GPCho(10:0/24:4(5Z,8Z,11Z,14Z))**	0.004121789	0.150283111
**PC 34:5; [M+H]+; GPCho(14:0/20:5(5Z,8Z,11Z,14Z,17Z))**	0.000170688	0.018733831
**PC 34:5; [M+H]+; GPCho(16:1(9Z)/18:4(6Z,9Z,12Z,15Z))**	0.000148995	0.018733831
**PC 35:1; [M+Na]+; GPCho(17:0/18:1(11E))**	0.000135654	0.018733831
**PC 35:3; [M+HCOO]-; GPCho(17:2(9Z,12Z)/18:1(11E))**	0.006601025	0.166525846
**PC 36:4; [M+Na]+; GPCho(12:0/24:4(5Z,8Z,11Z,14Z))**	0.008420747	0.186940583
**PC 36:4; [M+Na]+; GPCho(16:0/20:4(5E,8E,11E,14E))**	0.008767587	0.189584843
**PC 36:4; [M+Na]+; GPCho(18:2(2E,4E)/18:2(2E,4E))**	0.009567557	0.204230538
**PC 36:6; [M+H]+; GPCho(18:3(6Z,9Z,12Z)/18:3(6Z,9Z,12Z))**	0.012025954	0.241243543
**PC 36:8; [M+Na]+; GPCho(18:4(6Z,9Z,12Z,15Z)/18:4(6Z,9Z,12Z,1**	0.000105501	0.018733831
**PC 37:0; [M+H]+; GPCho(12:0/25:0)**	0.011336913	0.230194638
**PC 37:4; [M+H]+; GPCho(13:0/24:4(5Z,8Z,11Z,14Z))**	0.000197022	0.019296552
**PC 37:4; [M+H]+; GPCho(17:0/20:4(5E,8E,11E,14E))**	0.000118766	0.018733831
**PC 37:4; [M+H]+; GPCho(18:4(6Z,9Z,12Z,15Z)/19:0)**	0.000161295	0.018733831
**PC 38:7; [M+H]+; GPCho(18:2(2E,4E)/20:5(5Z,8Z,11Z,14Z,17Z))**	0.00691166	0.17175991

**PC 38:7; [M+H]+; GPCho(18:3(6Z,9Z,12Z)/20:4(5E,8E,11E,14E))**	0.007677337	0.180038964
**PC 39:1; [M-Ac-H]-; GPCho(18:1(11E)/21:0)**	0.012573397	0.246290659
**PC 40:1; [M-Ac-H]-; GPCho(18:1(11E)/22:0)**	0.002570761	0.142677249
**PC 40:5; [M+HCOO]-; GPCho(18:3(6Z,9Z,12Z)/22:2(13Z,16Z))**	0.001309591	0.113942359
**PC 40:5; [M+HCOO]-; GPCho(20:1(11E)/20:4(5E,8E,11E,14E))**	0.000590016	0.054576496
**PC 40:5; [M+HCOO]-; GPCho(20:2(11Z,14Z)/20:3(5Z,8Z,11Z))**	0.002485684	0.142677249
**PC 41:0; [M-Ac-H]-; GPCho(16:0/25:0)**	0.001380426	0.113942359
**PC 41:1; [M-Ac-H]-; GPCho(15:1(9Z)/26:0)**	0.001825743	0.117427191
**PC 41:1; [M-Ac-H]-; GPCho(18:1(11E)/23:0)**	0.001703324	0.117427191
**PC 42:0; [M-Ac-H]-; GPCho(18:0/24:0)**	0.001473135	0.113942359
**PC 42:1; [M-Ac-H]-; GPCho(18:1(11E)/24:0)**	0.007213731	0.174070475
**PC 42:5; [M+H]+; GPCho(20:1(11E)/22:4(7Z,10Z,13Z,16Z))**	0.00761659	0.180038964
**PC 42:6; [M-Ac-H]-; GPCho(20:2(11Z,14Z)/22:4(7Z,10Z,13Z,16Z))**	0.011233732	0.230194638
**PC 42:6; [M-Ac-H]-; GPCho(20:4(5E,8E,11E,14E)/22:2(13Z,16Z))**	0.012768836	0.247210612
**PE 34:2; [M+H]+; GPEtn(16:0/18:2(2E,4E))**	0.005941527	0.163990521
**PE 34:2; [M+H]+; GPEtn(16:1(7Z)/18:1(11E))**	0.004531868	0.150283111
**PE 35:2; [M+H]+; GPEtn(13:0/22:2(13Z,16Z))**	0.004866702	0.150283111
**PE 35:2; [M+H]+; GPEtn(17:0/18:2(2E,4E))**	0.004687222	0.150283111
**PE 35:2; [M+H]+; GPEtn(17:1(9Z)/18:1(11E))**	0.004874047	0.150283111
**PE 35:2; [M+H]+; GPEtn(9:0/26:2(5E,9Z))**	0.00483428	0.150283111
**PE 37:4; [M+H]+; GPEtn(17:0/20:4(5E,8E,11E,14E))**	0.004174319	0.150283111
**PE 37:5; [M+H]+; GPEtn(17:1(9Z)/20:4(5E,8E,11E,14E))**	0.000136271	0.018733831
**PG 40:6; [M-H]-; GPGro(18:2(2E,4E)/22:4(7Z,10Z,13Z,16Z))**	0.006492204	0.166300296
**PI 34:1; [M-H]-; GPIns(16:0/18:1(11E))**	0.004535112	0.150283111
**PS 36:3; [M-H]-; GPSer(16:0/20:3(5Z,8Z,11Z))**	0.006477545	0.166300296
**PS 36:3; [M-H]-; GPSer(18:0/18:3(6Z,9Z,12Z))**	0.007204929	0.174070475
**PS 36:3; [M-H]-; GPSer(18:1(11E)/18:2(2E,4E))**	0.008161446	0.183632525
**PS 38:2; [M-H]-; GPSer(18:0/20:2(11Z,14Z))**	0.00592106	0.163990521
**PS 38:2; [M-H]-; GPSer(18:1(11E)/20:1(11E))**	0.006246867	0.165095771
**PS 38:2; [M-H]-; GPSer(18:2(2E,4E)/20:0)**	0.007972605	0.18184092
**PS 42:5; [M-H]-; GPSer(18:1(11E)/24:4(5Z,8Z,11Z,14Z))**	0.010078835	0.212421013
**SM 32:0; [M]+; SM(d14:0/18:0)**	4.74597E-05	0.018733831
**SM 44:2; [M+Na]+; SM(d18:1(4E)/26:1(17Z))**	0.011029976	0.229561386
